# Cascade specific endogenous Fe^3+^ interference and *in situ* catalysis for tumor therapy with stemness suppression

**DOI:** 10.1093/nsr/nwae434

**Published:** 2024-11-29

**Authors:** Jiajie Chen, Yitong Wang, Jian Huang, Zhibo Yang, Huicong Niu, Xiaolian Su, Jimin Huang, Hongshi Ma, Yufang Zhu, Chengtie Wu, Jianlin Shi

**Affiliations:** State Key Laboratory of High Performance Ceramics and Superfine Microstructure, Shanghai Institute of Ceramics, Chinese Academy of Sciences, Shanghai 200050, China; Center of Materials Science and Optoelectronics Engineering, University of Chinese Academy of Sciences, Beijing 100049, China; Department of Radiology, Shanghai Tenth People's Hospital, School of Medicine, Tongji University, Shanghai 200072, China; Materials Genome Institute, Shanghai University, Shanghai 200444, China; State Key Laboratory of High Performance Ceramics and Superfine Microstructure, Shanghai Institute of Ceramics, Chinese Academy of Sciences, Shanghai 200050, China; Center of Materials Science and Optoelectronics Engineering, University of Chinese Academy of Sciences, Beijing 100049, China; Department of Neurology, Minhang Hospital, Fudan University, Shanghai 200032, China; Department of Radiology, Shanghai Tenth People's Hospital, School of Medicine, Tongji University, Shanghai 200072, China; State Key Laboratory of High Performance Ceramics and Superfine Microstructure, Shanghai Institute of Ceramics, Chinese Academy of Sciences, Shanghai 200050, China; Center of Materials Science and Optoelectronics Engineering, University of Chinese Academy of Sciences, Beijing 100049, China; State Key Laboratory of High Performance Ceramics and Superfine Microstructure, Shanghai Institute of Ceramics, Chinese Academy of Sciences, Shanghai 200050, China; Center of Materials Science and Optoelectronics Engineering, University of Chinese Academy of Sciences, Beijing 100049, China; State Key Laboratory of High Performance Ceramics and Superfine Microstructure, Shanghai Institute of Ceramics, Chinese Academy of Sciences, Shanghai 200050, China; Center of Materials Science and Optoelectronics Engineering, University of Chinese Academy of Sciences, Beijing 100049, China; State Key Laboratory of High Performance Ceramics and Superfine Microstructure, Shanghai Institute of Ceramics, Chinese Academy of Sciences, Shanghai 200050, China; Center of Materials Science and Optoelectronics Engineering, University of Chinese Academy of Sciences, Beijing 100049, China; State Key Laboratory of High Performance Ceramics and Superfine Microstructure, Shanghai Institute of Ceramics, Chinese Academy of Sciences, Shanghai 200050, China; Center of Materials Science and Optoelectronics Engineering, University of Chinese Academy of Sciences, Beijing 100049, China; Shanghai Frontiers Science Center of Nanocatalytic Medicine, Shanghai Tenth People's Hospital, School of Medicine, Tongji University, Shanghai 200331, China

**Keywords:** cancer stem-like cells, stemness inhibition, nanomedicine, endogenous Fe^3+^ interference, catalytic therapy

## Abstract

Cancer stem-like cells (CSCs), featuring high tumorigenicity and invasiveness, are one of the critical factors leading to the failure of clinical cancer treatment such as metastasis and recurrence. However, current strategies suffer from the low stemness-inhibiting efficacy on CSCs by conventional molecular agents and the poor lethal effects against bulk tumor cells. Here we engineer a coordination nanomedicine by 2,5-dihydroxyterephthalic acid (DHT) complexing zinc ions (Zn^2+^) as a double-effect nanodisrupter of tumor iron (Fe) and redox homeostasis for catalysis-boosted tumor therapy with stemness inhibition. Taking advantage of the much higher binding force of DHT toward Fe^3+^, this nanomedicine can specifically chelate endogenous Fe^3+^ into its nanostructure and release Zn^2+^, and the *in situ* formed hexacoordinated Fe-DHT conformation is of much enhanced reducibility in order to promote reactive oxygen species (ROS) production in tumors. The nanomedicine-mediated Fe depletion and ROS generation collectively induce CSC differentiation *via* downregulating the Wnt signaling and inducing forkhead box O3 (FoxO3) activation, respectively. Notably, the combined tumor-selective ROS generation and Zn^2+^-induced antioxidation dysfunction potently trigger intratumoral oxidative damage leading to both cellular apoptosis and ferroptosis. This nanomedicine, capable of synchronously treating CSCs and bulk tumor cells, has been demonstrated to effectively inhibit the growth, postoperative recurrence and metastasis of orthotopic triple-negative breast tumors *in vivo*, offering an encouraging candidate of cancer therapeutic agents for treating CSCs-enriched malignancy.

## Teaser

A coordination nanomedicine was developed to simultaneously treat cancer stem-like cells (CSCs) and bulk tumor cells by interfering with tumor Fe and redox homeostasis, thereby effectively inhibiting tumor growth, recurrence and metastasis.

## INTRODUCTION

Metastasis, recurrence, and chemotherapeutic resistance have been the primary obstacles to the success of malignancy therapy [1,2]. Accumulating evidence substantiates that the existence of a small subset of tumor cells with stem cell characteristics (i.e. cancer stem-like cells (CSCs)), which possess self-renewal, phenotypic plasticity, high tumorigenicity and invasiveness, etc., is the critical factor accounting for these hurdles to clinically efficacious cancer treatment [3–5]. As distinct from other anti-CSC strategies, differentiation therapy [6], proposed as a considerably promising approach to deplete CSC populations, is typically enabled by employing molecular agents (e.g. all-*trans* retinoic acid (ATRA), vitamin D3, histone deacetylase inhibitor (HDACi)) to disrupt CSC plasticity and induce the differentiation of CSCs into more mature tumor cells (i.e. non-CSCs) with less stemness and aggressiveness [7–9]. However, current differentiation therapy with these molecular agents suffers from unsatisfactory differentiation-inducing efficacy due to poor delivery and targeting, potentially failing to eradicate the CSC pool. Taking advantage of nanotechnology, diverse nanomaterials have been engineered as delivery vehicles to improve the bioavailability and specificity of these small molecules for augmenting treatment [10]. Alternatively, a handful of advanced nanomaterials (e.g. metallofullerenol nanoparticles [11], graphene oxide [12], chemically modified gold nanoparticles [13]) themselves have also shown their ability to differentiate CSCs. Nevertheless, it is worth noting that single differentiation therapy is limited by lacking powerful tumor-specific lethal action, resulting in massive bulk tumor cells remaining that can convert to CSCs *via* epithelial-to-mesenchymal transition (EMT) [14,15]. Although several combinations of different drugs/therapeutics based on elaborate nanoplatforms have been reported for anti-CSCs and tumor elimination [16–18], complicated preparation and operation as well as inevitable side effects would be a great challenge for their practical application. There is, accordingly, an urgent need for new feasible and safe nanoformulations capable of concurrently effectively differentiating CSCs and specifically killing bulk tumor cells.

Iron (Fe) is known to be associated with tumorigenesis and cancer progression [19]. Recent studies indicate the importance of Fe metabolism in CSCs [20,21]. CSCs exhibit an enhanced dependence on Fe compared to non-CSC counterparts, resulting from its vital role in CSC maintenance and self-renewal activation [22]. Based on this, an Fe depletion strategy with available Fe chelators has recently been demonstrated to suppress CSC-related signaling pathways and stemness expressions [21,23,24], but is still restricted by the inherent shortcomings of small molecules and the unsatisfactory cytotoxic effect on tumor cells. In particular, Fe is related to cellular redox homeostasis, due to its ability to participate in redox reactions (typically Fenton reactions) for reactive oxygen species (ROS) generation. Despite the pro-tumor mechanisms of ROS, overproduction of them within tumor cells induces detrimental oxidative stress and can cause cell death *via* apoptosis and/or non-apoptotic pathways, such as newly identified Fe-dependent ferroptosis that proves to be more offensive toward tumor cells [25–27]. Typically, CSCs possess a high oxidation resistance, capable of sustaining the low intracellular ROS levels which are of benefit for their traits [28]. Growing evidence has revealed that elevation of ROS boosts CSC differentiation [13,29,30]. From this perspective, concurrent manipulation of intracellular Fe metabolism and ROS generation may represent an attractive therapeutic strategy for synergistically eliminating CSCs and bulk tumor cells, but remains highly challenging due to the lack of effective therapeutics to drive such a therapeutic pathway.

**Figure 1.**
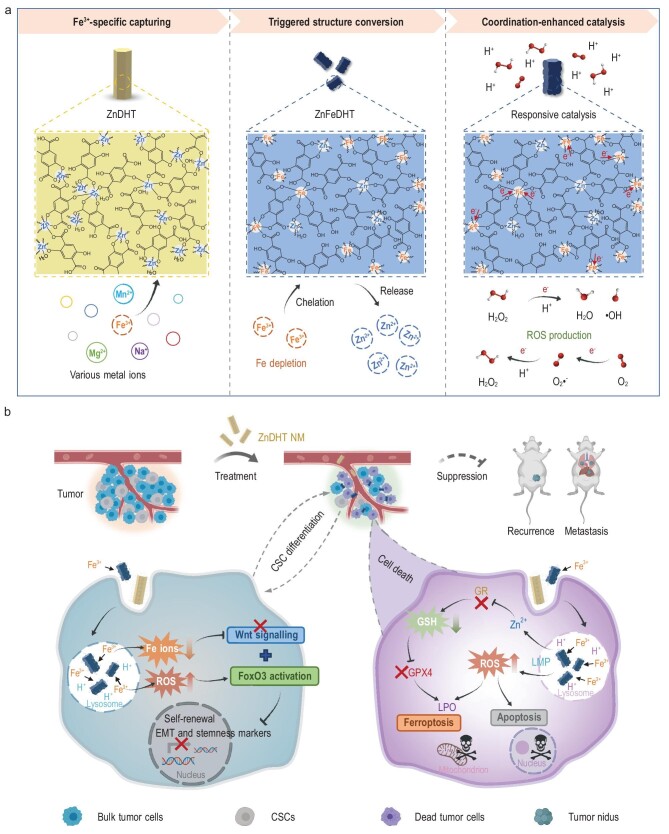
Schematic illustrations of the Fe^3+^-triggered cascade functioning of ZnDHT and its mediated anti-cancer mechanisms. (a) The constructed ZnDHT can specifically capture Fe^3+^ owing to the preferential Fe^3+^ binding force of DHT, leading to the breakage of partial Zn-O bonds and the formation of a hexacoordinated Fe-DHT structure in the ZnFeDHT, enabling environmental Fe depletion and the release of Zn^2+^. Then, the highly reductive Fe centers in the Fe-DHT structure of ZnFeDHT catalyze ORRs and Fenton reactions under the acidic condition for ROS (O_2_•^−^ and •OH) production. (b) The ZnDHT NM chelates tumor extracellular and intracellular Fe ions and accumulates in the lysosomes, resulting in the Fe depletion, ROS generation, and Zn^2+^ release, which could not only inhibit the EMT and CSC stemness, but also cause tumor cell oxidative damage, resultantly activating both apoptosis and ferroptosis of tumor cells. Based on the strong capacity to simultaneously treat CSCs and bulk tumor cells, the nanomedicine could suppress the tumor growth, recurrence and metastasis *in vivo*.

Herein, a novel and potent anti-cancer strategy based on a coordination nanomedicine featuring cascade specific Fe^3+^ capturing and intratumoral oxidative damage induction is proposed (Fig. 1). Benefiting from its two symmetric sets of oxygen donors stemming from both phenolic and carboxylic functional groups, 2,5-dihydroxyterephthalic acid (DHT) acts as an attractive organic molecule that makes the construction of diverse and versatile coordination polymers possible [31]. Here we constructed a uniform coordination nanoparticle (ZnDHT) by complexing non-toxic zinc ions (Zn^2+^) using DHT, which can specifically and continuously capture environmental Fe^3+^ to form a more stable hexacoordinated Fe-DHT structure by strong Fe-O bonding, accompanied by the breaking of partial Zn-O bonds and the release of Zn^2+^ (Fig. 1a). Based on theoretical simulation analysis, this Fe^3+^-selective chelation of ZnDHT was demonstrated to be originated from the much higher binding force of DHT toward Fe^3+^ than other common metal ions such as Zn^2+^. Interestingly, in the newly assembled composite coordination nanostructure (termed as ZnFeDHT), the strong metal-ligand exchange coupling between Fe centers and DHT ligands leads to a remarkable electronic delocalization, making the Fe-coordinated DHT a qualified electron donator promoting ligand-to-metal reduction. As a result, the active Fe sites (Fe*^δ^*^+^, 2 < *δ* < 3) of strong reducibility can not only effectively catalyze Fenton reactions in the hydrogen peroxide (H_2_O_2_)-existing acidic environment to produce highly reactive hydroxyl radicals (•OH), but also, more importantly, promote electron transfer from DHT ligands to surrounding oxygen molecules (O_2_) for oxygen reduction reactions (ORRs) producing superoxide anions (O_2_•^−^) and subsequent H_2_O_2_ fueling of Fenton reactions, ultimately boosting ROS generation.

Given the characteristics discovered, we then focused on the potential cancer therapy of biocompatible chitosan (CS)-modified ZnDHT nanomedicine (denoted as ZnDHT NM) (Fig. 1b). This nanomedicine was demonstrated to chelate endogenous Fe ions and accumulate in the lysosomes, thereby inducing pronounced ROS generation within tumor cells. Synchronously, ZnDHT NM-mediated Fe depletion blocked the Wnt signaling pathway to inhibit the EMT and CSC stemness, which was facilitated by as-triggered ROS elevation *via* forkhead box O3 (FoxO3) activation, resulting in the elimination of CSC populations in a much stronger way than by conventional Fe chelating agent, deferoxamine mesylate (DFOM), and chemotherapeutic drug, doxorubicin (DOX). Additionally, by combining the tumor-selective ROS production and leaked Zn^2+^-induced glutathione reductase (GR) inactivation, ZnDHT NM enabled remarkable redox dyshomeostasis within tumor cells, triggering cellular oxidative damage and activating an impressive apoptosis-ferroptosis synergistic effect to kill tumor cells without noticeable cytotoxicity to normal cells. Therefore, ZnDHT NM is able to simultaneously treat CSCs and bulk tumor cells. Further *in vivo* mouse models manifested that such a simple nanomedicine effectively suppressed the growth of orthotopic triple-negative breast cancer and prevented its postsurgical recurrence and metastasis.

## RESULTS AND DISCUSSION

### Fe^3+^-selective capturing and structure transformation of ZnDHT

**Figure 2.**
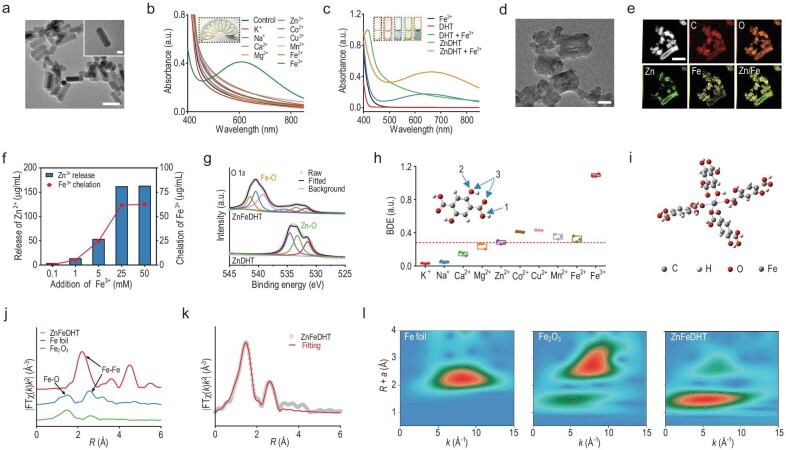
Characterizations of ZnDHT and its Fe^3+^-specific chelation performance. (a) Transmission electron microscopy (TEM) images of ZnDHT. Scale bar, 200 nm, 50 nm (inset). (b, c) UV-Vis absorption spectra of ZnDHT after being dispersed in solutions containing different kinds of metal ions (b), and DHT and ZnDHT after being dispersed in the solution with or without addition of Fe^3+^ (c). Inset: the photographs of these tested solutions after reactions. (d, e) TEM image (d), high angle annular dark-field (HAADF) image and element mappings (e) of ZnFeDHT prepared by reacting ZnDHT (1 mg/mL) with Fe^3+^ (5 mM). Scale bar in (d), 50 nm. Scale bar in (e), 200 nm. (f) The Zn^2+^ release and Fe^3+^ chelation behaviors of ZnDHT in the solution containing different concentrations of Fe^3+^. (g) O 1*s* XPS spectra of ZnDHT and ZnFeDHT. (h) The calculated BDEs between DHT and different metal ions in the three coordinated modes (**M**-O carboxylate, **M**-O phenate, and O-**M**-O bridge-type). Red dashed line indicates the average BDE between DHT and Zn^2+^ in these different coordinated modes. (i) The optimized hexacoordinated Fe-DHT conformation based on DFT calculations. (j) Fe K-edge EXAFS spectra of ZnFeDHT, Fe foil, and Fe_2_O_3_. (k) Corresponding EXAFS fitting curve of ZnFeDHT in *R* space. (l) EXAFS wavelet transforms of ZnFeDHT, Fe foil, and Fe_2_O_3_.

Significantly different from typical non-uniform microscale Zn^2+^-DHT coordination materials [32], here the ZnDHT with a uniform and well-defined morphology was prepared using zinc acetate (Zn(CH_3_COO)_2_) and DHT as starting materials through a one-step solvothermal reaction employing H_2_O as a size modulator [33], resulting in rod-like particles with the diameters from nanoscale to micron (Fig. S1). The preferred nanosized ZnDHT (∼70 nm in width and ∼195 nm in length) was selected for the further studies conducted in this work (Fig. 2a, and Fig. S2a and b). These solvothermal-synthesized coordination nanoparticles exhibit a well-crystallized structure (Fig. S2c and d). Additionally, energy dispersive spectroscopy (EDS) element mapping and Fourier transform infrared (FTIR) analyses evidence the uniform distribution of C, O, and Zn elements on the nanoparticle (Fig. S2e and f) and the formation of ZnDHT through coordinating Zn^2+^ with both phenate and carboxylate oxygens in DHT molecules (Fig. S3a). ZnDHT shows a similar ultraviolet-visible (UV-Vis) absorption spectrum with a certain degree of redshift compared to DHT (Fig. S3b). ZnDHT could be uniformly dispersed in water and had a well-defined size distribution of around 203.0 nm in average hydrodynamic diameter (Fig. S3c and d).

We then estimated the responses of ZnDHT to various nutritional metal ions (for instance, K^+^, Na^+^, Ca^2+^, Mg^2+^, Zn^2+^, Co^2+^, Cu^2+^, Mn^2+^, Fe^2+^, and Fe^3+^). To our surprise and encouragingly, the color and UV-Vis absorption spectra of the ZnDHT suspension (faint yellow) added with other metal ions did not change significantly except for the Fe^3+^-ZnDHT mixture, which displayed a deep blue color and a characteristic absorption peak at 662 nm (Fig. 2b, and Fig. S4a and b). Similar to ZnDHT, the color change and UV-Vis characteristic absorption were also observed as mixing DHT molecules with Fe^3+^ (Fig. 2c), indicating a certain interaction between them. Moreover, different Fe salts with the same concentration induced a comparative color change of the mixtures, illustrating that the reaction of ZnDHT with Fe^3+^ is not affected by other anions (Fig. S4c). After Fe^3+^ intrusion, integral ZnDHT exhibited an obvious collapse accompanied by a transition from crystalline to amorphous state, while other metal ions caused no or negligible impact on its crystallinity (Fig. 2d and Fig. S5). As shown in the EDS element mappings, Fe atoms exist in the framework of nanoparticles (forming ZnFeDHT) (Fig. 2e). In the wake of the added Fe^3+^ increase, more Fe atoms presented on the ZnFeDHT but the intrinsic Zn atoms continued to diminish and release into the solution until the complete collapse of the initial structure (Fig. 2f and Fig. S6). X-ray photoelectron spectroscopy (XPS), FTIR, and Raman measurements jointly confirm the newly formed Fe-O bonds between the phenate and carboxylate oxygens of DHT ligands and Fe atoms in the ZnFeDHT resulting from the Fe chelation by DHT (Fig. 2g and Figs S7–S9).

To disclose the Fe^3+^-selective chelation mechanism of ZnDHT, we performed density functional theory (DFT) calculations on the DHT molecule and its coordination with different metal ions. The higher bond dissociation energy (BDE) means a stronger binding force. The BDEs between DHT and Fe^3+^ in three possible coordinated modes (Fe-carboxylate oxygen, Fe-phenate oxygen, and bridge-type coordination) were calculated to be 1.079, 1.080, and 1.110 a.u., respectively, much higher than that between DHT and other different metal ions (including monovalent, divalent, and trivalent states) (Fig. 2h, Fig. S10 and Table S1). Specifically, the binding force of DHT to Fe^3+^ is largely stronger than to Zn^2+^. The mixture of Fe^3+^ and DHT tends to form a thermodynamically stable hexacoordinated Fe-DHT conformation (i.e. one Fe^3+^ coordinates with the carboxylate and phenate oxygens of four DHT molecules through six Fe-O bonds), as evidenced by structural optimization based on energy minimization from DFT calculations (Fig. 2i). According to Fe K-edge extended X-ray absorption fine structure (EXAFS) and wavelet transform analysis, the Fe atoms in the ZnFeDHT are dominantly coordinated with oxygen atoms in a coordination number of 5.9, which is consistent with the theoretical calculations (Fig. 2j–l, Fig. S11, and Table S2). Furthermore, we calculated the BDEs between DHT and different trivalent metal ions (e.g. Mo^3+^, Al^3+^, Cr^3+^, Ga^3+^, and Fe^3+^) in a similar hexacoordinated mode, also proving that DHT has the highest binding force toward Fe^3+^ (Table S3). These results suggest that the complexing of DHT and Fe^3+^ is energy-favorable, and the relatively stronger binding force between Fe^3+^ and DHT leads to breakage of the original Zn-O bonds of ZnDHT, the formation of a stable hexacoordinated Fe-DHT structure in the ZnFeDHT, and the release of Zn^2+^.

The Fe^3+^ chelation behaviors of DHT and ZnDHT were compared based on the UV-Vis characteristic absorption of the formed a blue Fe-DHT complex. Instead of the instantaneous chelation by DHT, ZnDHT exhibits a slow and sustained chelation effect toward Fe^3+^ with a higher efficiency (Fig. S12). In addition, ZnDHT as a Fe^3+^ capturing agent displays good pH stability, and its Fe^3+^-specific chelation is irreversible in simulated physiological environments (Fig. S13), benefiting Fe depletion in tumor cells and tissues.

### Potentiating ROS production by Fe^3+^-activated ZnDHT

**Figure 3.**
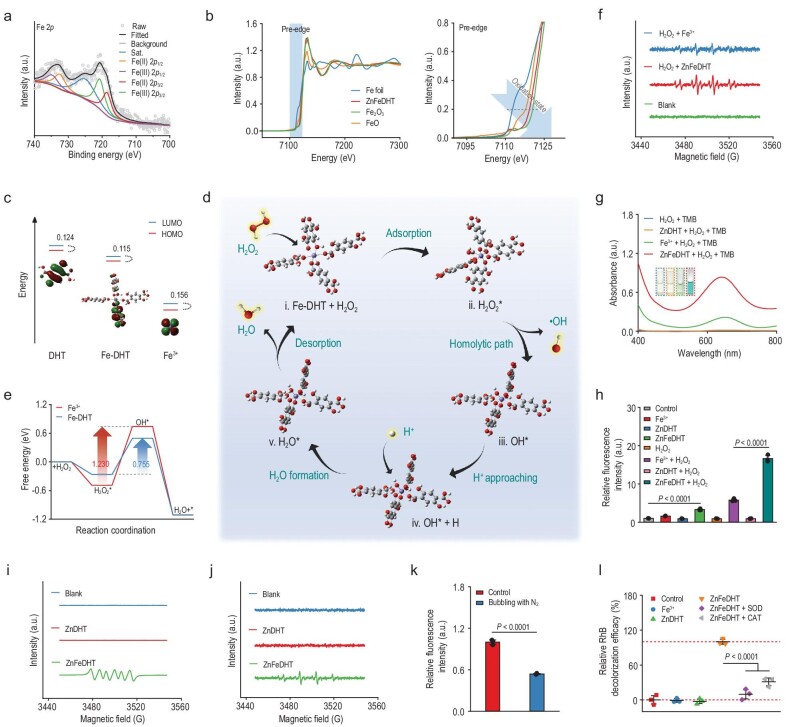
Redox potential and ROS-generating performance of formed ZnFeDHT and corresponding theoretical calculations. (a) Fe 2*p* XPS spectrum of ZnFeDHT. (b) Fe K-edge XANES and extracted pre-edge spectra of ZnFeDHT, Fe foil, Fe_2_O_3_, and FeO. (c) The HOMO/LUMO energy levels of DHT, Fe^3+^, and Fe-DHT complex. The values in the figure represent the corresponding energy gaps (a.u.), and the insert images represent the calculated HOMO orbitals of different species. (d) Proposed catalytic mechanism of the optimized hexacoordinated Fe-DHT conformation in the Fenton reaction toward producing •OH. (e) Corresponding free energy diagrams for catalytic Fenton reactions on the Fe^3+^ and Fe-DHT. Arrows indicate the corresponding energy barriers. (f) ESR spectra evaluating •OH generation in the H_2_O_2_-containing buffer solution (pH 5.2) containing free Fe^3+^ or fresh ZnFeDHT. (g) UV-Vis absorption spectra of the H_2_O_2_-contained buffer solutions (pH 5.2) with TMB as a colorimetric indicator in the presence of ZnDHT, Fe^3+^, or fresh ZnFeDHT. Inset: photographs of these tested solutions after reactions. (h) Relative fluorescence intensities of the buffer solutions (pH 5.2) under different settings and with OPD as a fluorescent indicator (*n* = 3). (i, j) ESR spectra evaluating O_2_•^−^ (i) and •OH (j) generation in the solutions containing ZnDHT or fresh ZnFeDHT. (k) Relative fluorescence intensities of the buffer solutions (pH 5.2) with OPD as a fluorescent indicator in the presence of fresh ZnFeDHT with or without N_2_ bubbling (*n* = 3). (l) RhB decolorization in the buffer solution (pH 5.2) with different settings (*n* = 3). Statistical significance was determined by one-way ANOVA with Tukey's *post-hoc* test (h, l) or two-tailed Student's *t*-test (k).

As a hydroquinone derivative, DHT has been reported for efficient Fe^3+^ reduction [34]. Accordingly, the redox status of Fe centers of the Fe-DHT structure in ZnFeDHT was investigated by Fe 2*p* XPS spectroscopy, where the pristine characteristic peaks were split into several sub-peaks assigned to Fe(II) 2*p*1/2, Fe(III) 2*p*3/2, Fe(II) 2*p*3/2, Fe(III) 2*p*3/2, and satellite signal (Fig. 3a). Furthermore, the Fe K-edge of X-ray absorption near-edge structure (XANES) in the ZnFeDHT was proved to be located in between those of FeO and Fe_2_O_3_ reference samples, implying that the chemical valence state of Fe is in between +2 and +3 (Fig. 3b), indicating that the Fe centers have certain redox potentials of divalent Fe under the coordination field effect by DHT ligands. Cyclic voltammetry (CV) measurement of Fe-DHT complex also demonstrates Fe sites in the coordinated structure with enhanced reducibility, as evidenced by the significantly weakened peak in the original reduction process (Fig. S14). On the basis of the DFT calculations, the spin density of the hexacoordinated Fe-DHT conformation is mainly localized on the Fe center (Fig. S15). The strong metal-ligand exchange coupling between Fe centers and DHT ligands results in an obvious electronic delocalization in the Fe-DHT complex to facilitate a ligand-to-metal reduction, leading to the Fe centers being in a high-spin state and displaying strong reducibility [35]. The calculated atomic charge distributions indicate the occurrence of partial electron transfer from the DHT ligand to the coordinated Fe atom after coordinating DHT with Fe^3+^ (Table S4). According to the highest occupied molecular orbital (HOMO)/lowest unoccupied molecular orbital (LUMO) energy levels of different species (Fig. 3c, and Tables S5 and S6), the much higher HOMO makes DHT ligands an electron donator which advantages the reduction reaction on Fe centers, whereas the active Fe sites as an electron transporter promote ligand oxidation in the coordination structure. On the other hand, the energy gap between HOMO and LUMO of Fe-DHT (0.115 a.u.) is also narrowed compared to that of Fe^3+^ (0.156 a.u.) and DHT (0.124 a.u.), suggesting the hexacoordinated Fe-DHT structure efficaciously improves chemical reactivity according to the frontier orbital theory.

Fenton chemistry has recently attracted much attention for its great potential in tumor catalytic therapy [36], while its efficiency is undermined by the inefficient conversion of Fe^3+^ to Fe^2+^ during Fenton reactions (1–3) [37]:


(1) \begin{eqnarray*}
{\mathrm{F}}{{{\mathrm{e}}}^{2 + }} &+&\, {{{\mathrm{H}}}_2}{{{\mathrm{O}}}_2} \to {\mathrm{F}}{{{\mathrm{e}}}^{3 + }} + {\mathrm{O}}{{{\mathrm{H}}}^ - }\\
&&\quad \quad + \bullet {\mathrm{OH}}({{k}_1} = 55\,\,{{{\mathrm{M}}}^{ - 1}}{{{\mathrm{s}}}^{ - 1}}),
\end{eqnarray*}

(2) \begin{eqnarray*}
{\mathrm{F}}{{{\mathrm{e}}}^{3 + }} &+& {{{\mathrm{H}}}_2}{{{\mathrm{O}}}_2} \to {\mathrm{F}}{{{\mathrm{e}}}^{2 + }} + \bullet {\mathrm{OOH}}\\
&+& {{{\mathrm{H}}}^ + }({{k}_2} = 7.13 \times {{10}^{ - 6}}\,\,{{{\mathrm{M}}}^{ - 1}}{{{\mathrm{s}}}^{ - 1}}),
\end{eqnarray*}

(3) \begin{eqnarray*}
{\mathrm{F}}{{{\mathrm{e}}}^{3 + }} &+& \bullet {\mathrm{OOH}} \to {\mathrm{F}}{{{\mathrm{e}}}^{2 + }} + {{{\mathrm{O}}}_2}. \\
&&+\, {{{\mathrm{H}}}^ + }({{k}_3} = 2\times {{10}^4}\,{{{\mathrm{M}}}^{ - 1}}{{{\mathrm{s}}}^{ - 1}}).\end{eqnarray*}

The potentiated reductive transformation of Fe^3+^ in the Fe-DHT structure stemming from the DHT coordination inspired us to examine the catalytic activity of ZnFeDHT on the production of highly reactive •OH from H_2_O_2_  *via* Fenton reactions. Electron spin resonance (ESR) spectroscopy shows stronger •OH signals in the ZnFeDHT group than in the Fe^3+^ group (the same Fe content tested here) with the addition of H_2_O_2_ (Fig. 3f). Both typical 3,3',5,5'-tetramethylbenzidine (TMB) colorimetric analysis and methylene blue (MB) bleaching assay attested that the catalytic activity of ZnFeDHT on converting H_2_O_2_ to •OH is obviously superior to that of free Fe^3+^, while negligible •OH generation was found in the H_2_O_2_-containing ZnDHT group (Fig. 3g and Fig. S16). The •OH generation catalyzed by ZnFeDHT shows a distinct acidic pH-dependency (Fig. S17), presumably attributable to the acidity-promoted Fenton reactions. During the catalytic process in the H_2_O_2_-containing acidic buffer, the Fe ions were proven to be hardly detached from the nanoparticles (Fig. S18), implying the integrity of the Fe-DHT coordination structure after redox reactions. Thus, the Fe-DHT conformation of ligand-to-metal reduction potential could accelerate the Fe(III)/Fe(II) redox cycling within the ZnFeDHT during the Fenton reaction process, thereby boosting the catalysis of •OH generation.

Mechanistically, based on the optimized hexacoordinated Fe-DHT conformation as a model, a plausible catalytic mechanism was proposed by DFT calculations (Fig. 3d), and corresponding Gibbs free energies for these transition states are shown in Fig. 3e. Thereinto, forming free •OH is the rate-limiting step, where the energy barrier for Fe-DHT (0.755 eV) is much lower than that for Fe^3+^ (1.230 eV). From a thermodynamic perspective, the Fe-DHT structure is more conducive to •OH production. Moreover, a protonated hydrogen atom is required to bind with the adsorbed hydroxyl group for H_2_O formation with substantially reduced Gibbs free energy, demonstrating again the thermodynamically favorable Fenton catalytic reaction in an acidic environment [38].

To further assess the ROS-generating performance of ZnFeDHT, we carried out ROS detection based on a fluorescent indicator, *o*-phenylenediamine (OPD). Besides the markedly stronger ROS signals detected for the ZnFeDHT than for the Fe^3+^ under H_2_O_2_ supply, surprisingly, the ZnFeDHT without the assistance of H_2_O_2_ also induced ROS generation in an acidity- and nanoparticle concentration-dependent manner (Fig. 3h and Fig. S19). More specifically, the ESR results confirmed both O_2_•^−^ and •OH generation induced by ZnFeDHT in the absence of H_2_O_2_, while no radical signals were found in the ESR spectra of the ZnDHT group (Fig. 3i and j), illustrating that Fe species in the ZnFeDHT is decisive for inducing ROS generation. The detailed ROS-generating process of ZnFeDHT was then investigated. ZnFeDHT-induced ROS production was visibly inhibited by depleting dissolved O_2_ in the test solution (Fig. 3k). In the •OH-mediated rhodamine B (RhB) decolorization assay, the RhB-decolorizing efficacy of ZnFeDHT was greatly compromised after superoxide dismutase (SOD) or catalase (CAT) addition (Fig. 3l). These results indicate that O_2_ is an indispensable and favored reactant for O_2_•^−^ and H_2_O_2_ production, which act as the essential intermediates for ultimate •OH formation by catalytic Fenton reactions in this ROS-generating process. Combined with the redox potentials of Fe-DHT coordination structure, and the reported hydroquinone oxidation (involving two sequential 1e^−^ transfer steps to generate a semiquinone radical intermediate and the subsequent *o*-quinone product) [39,40], we propose the following pathway of ORRs on the Fe centers along with DHT oxidation. Cozzolino *et al.* first reported the DHT ligand oxidation to the quinone DHT in a coordination complex without damaging its structural integrity, and the electron transfer involved in the metal nodes [41]. Accordingly, based on our previous investigation on the oxygen-reduction function of Fe-polyphenol complex [42], the ORRs mechanism of Fe-DHT complex for forming O_2_•^−^ and subsequent H_2_O_2_ could be reasonably proposed as follows (4–8):


(4) \begin{eqnarray*}
{\mathrm{F}}{{{\mathrm{e}}}^{3 + }} + {\mathrm{DH}}{{{\mathrm{T}}}^{4 - }} \to {{\left[ {{\mathrm{F}}{{{\mathrm{e}}}^{{\mathrm{III}}}}\!-\!{\mathrm{DHT}}} \right]}^ - },\end{eqnarray*}

(5) \begin{eqnarray*}
{{\left[ {{\mathrm{F}}{{{\mathrm{e}}}^{{\mathrm{III}}}}\!-\!{\mathrm{DHT}}} \right]}^ - } \leftrightarrow {{\left[ {{\mathrm{F}}{{{\mathrm{e}}}^{{\mathrm{II}}}}\!-\!{\mathrm{sqDHT}} \bullet } \right]}^ - },\end{eqnarray*}

(6) \begin{eqnarray*}
&&{{\left[ {{\mathrm{F}}{{{\mathrm{e}}}^{{\mathrm{II}}}}\!-\!{\mathrm{sqDHT}} \bullet } \right]}^ - } + {{{\mathrm{O}}}_2} \\
&&\quad\quad\quad\to \left[ {{\mathrm{F}}{{{\mathrm{e}}}^{{\mathrm{III}}}}\!-\!{\mathrm{sqDHT}} \bullet } \right] + {{{\mathrm{O}}}_2}{{ \bullet }^ - },\end{eqnarray*}

(7) \begin{eqnarray*}
\left[ {{\mathrm{F}}{{{\mathrm{e}}}^{{\mathrm{III}}}}\!-\!{\mathrm{sqDHT}} \bullet } \right] \leftrightarrow \left[ {{\mathrm{F}}{{{\mathrm{e}}}^{{\mathrm{II}}}}\!-\!{\mathrm{qDHT}}} \right],\end{eqnarray*}

(8) \begin{eqnarray*}
&&\left[ {{\mathrm{F}}{{{\mathrm{e}}}^{{\mathrm{II}}}}\!-\!{\mathrm{qDHT}}} \right] + {{{\mathrm{O}}}_2}{{ \bullet }^ - } + 2{{{\mathrm{H}}}^ + } \\
&&\quad\quad\quad\to {{\left[ {{\mathrm{F}}{{{\mathrm{e}}}^{{\mathrm{III}}}}\!-\!{\mathrm{qDHT}}} \right]}^ + }+ {{{\mathrm{H}}}_2}{{{\mathrm{O}}}_2}.\end{eqnarray*}

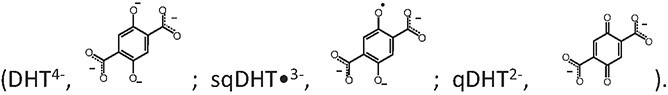


FTIR and Raman analyses reveal oxidation of the DHT ligand and the formation of quinone in the ZnFeDHT, as evidenced by the detected signals of C=O stretching of benzoquinone (Figs S8 and S9) [41,43]. DFT calculations also suggest the thermodynamic feasibility of these two cascade chemical reactions from the negative formation energies (Fig. S20).

Collectively, the cooperative effect of the Fe-DHT coordination structure-facilitated Fenton reactions and the ORRs-enabled H_2_O_2_ supplementation on elevating the highly reactive •OH generation would be beneficial for effective catalytic therapy against tumors, within which the concentration of overexpressed endogenous H_2_O_2_ is too low to initiate large-scale Fenton reactions [44]. The acidity-responsive nature of these reactions suggests that the Fe^3+^-activated ZnDHT (i.e. ZnFeDHT) is catalytically active for ROS production specifically in the acidic environment of tumors, favoring safe therapeutic application [45]. Alternatively, as a Fe chelating agent for clinical use, DFOM displays a stronger Fe chelating ability than ZnDHT due to its advantageous chemical structure, but unfortunately tends to form an inert coordination complex without significant redox-catalytic activity for ROS generation (Figs S21 and S22, and Tables S5 and S7). Hence, the ZnDHT featuring both Fe^3+^-capturing and the concomitant ROS-producing activities is a highly promising nanoformulation for meeting therapeutic requirements.

### *In vitro* regulation of stemness-associated characteristics of CSCs

**Figure 4.**
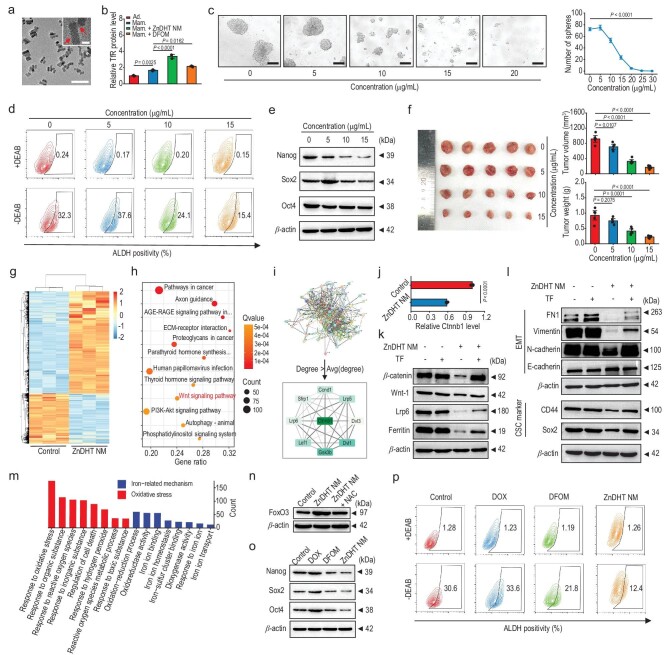
CSC stemness-inhibiting capacity of ZnDHT NM. (a) TEM images of ZnDHT NM. Red arrows indicate the decorated CS layer on the surface of nanoparticles. Scale bar, 500 nm, 50 nm (inset). (b) ELISA analysis of TfR protein expressions in the different treated cells (*n* = 3). (c) Tumorsphere formation of 4T1 mammosphere cells cultivated in the serum-free culture medium with treatment of ZnDHT NM for 4 days. The counted numbers of tumorspheres (diameter >50 μm) (*n* = 4). Scale bar, 100 μm. (d) Flow cytometric analysis of the proportion of ALDH^high^ cells sorted from mammosphere cells upon treatment with ZnDHT NM. *N,N*-diethylaminobenzaldehyde (DEAB) acts as an ALDH inhibitor. (e) Western blot analysis of the Nanog, Sox2, and Oct4 protein expressions in mammosphere cells. (f) Images, volumes, and weights of the tumors of mice on day 21 post inoculation of 4T1 mammosphere cells upon treatment with ZnDHT NM (*n* = 5). (g) The heatmap of DE mRNAs (*n* = 3). (h) KEGG enrichment analysis of the top 12 downregulated pathways. (i) PPI network for the DE mRNAs associated with the Wnt signaling pathway, and the extracted PPI network (degree > average degree; the network nodes with darker colors represent higher degrees; the edges with darker colors represent higher combined scores). (j) RT-qPCR analysis of the relative *Ctnnb1* gene expression (*n* = 3). (k, l) Western blot analysis of the expressions of the Wnt signaling target proteins (k), the cellular EMT and CSC markers (l) upon treatment with ZnDHT NM and/or TF. (m) GO enrichment analysis of the biological processes associated with Fe-related mechanisms and oxidative stress. (n) Western blot analysis of the FoxO3 protein expression in ZnDHT NM-treated cells with or without the addition of NAC. (o, p) Western blot analysis of the Nanog, Sox2, and Oct4 protein expressions (o) and flow cytometric analysis of the ALDH^high^ cell proportion (p) in 4T1 mammosphere cells upon different treatments. Statistical significance was determined by one-way ANOVA with Tukey's *post-hoc* test (b, c, f) or two-tailed Student's *t*-test (j).

**Figure 5.**
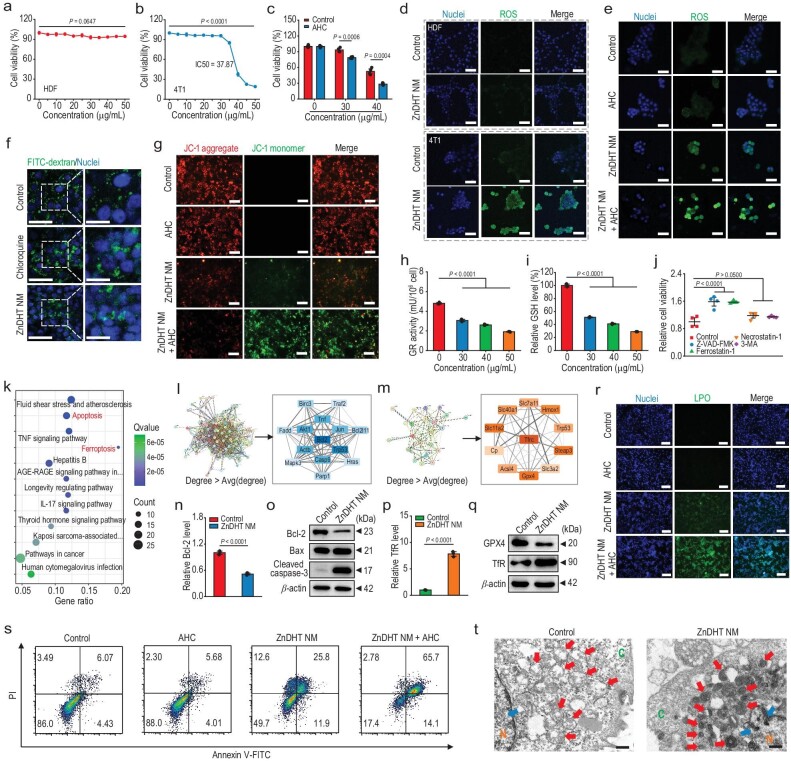
*In vitro* tumor cell killing effects of ZnDHT NM. (a, b) Cell viabilities of the ZnDHT NM-treated HDF cells (a) and 4T1 cells (b) (*n* = 4). IC50 represents the half maximal inhibitory concentration. (c) Cell viabilities of the ZnDHT NM-treated 4T1 cells under neutral culture medium (pH 7.4) or the AHC medium (pH 6.0; H_2_O_2_, 100 μM) (*n* = 4). (d, e) 2′,7′-Dichlorofluorescin diacetate (DCFH-DA)–based fluorescent detection of ROS produced in the ZnDHT NM-treated HDF cells and 4T1 cells (d) or in 4T1 cells after different treatments (e). Scale bar, 50 μm. (f) Fluorescence images presenting the subcellular localization of bulky FITC-dextran in 4T1 cells treated with chloroquine (as positive control) or ZnDHT NM. The diffusive fluorescence distribution indicates the occurrence of intracellular LMP. Scale bar, 50 μm, 25 μm. (g) Fluorescence images of JC-1 staining of 4T1 cells. Scale bar, 50 μm. (h, i) Cellular GR activity (h) and GSH level (i) evaluation of the ZnDHT NM-treated 4T1 cells (*n* = 3). (j) Relative cell viabilities of the ZnDHT NM-treated 4T1 cells with the additions of different death inhibitors (*n* = 4). (k) KEGG enrichment analysis of the top 12 pathways associated with oxidative stress. (l, m) PPI networks for the DE mRNAs associated with apoptosis (l) and ferroptosis (m) pathways, and the corresponding extracted PPI network (degree > average degree). (n, p) RT-qPCR analysis of the relative *Bcl-2* (n) and *TfR* (i.e, *Tfrc*) (p) gene expressions in the ZnDHT NM-treated 4T1 cells (*n* = 3). (o, q) Western blot analysis of the apoptosis- (o) and ferroptosis-related (q) proteins in 4T1 cells with or without ZnDHT NM treatment. (r, s) Fluorescent images of BODIPY™ 581/591 C11 staining (r) and flow cytometric analysis of Annexin V-FITC/PI staining (s) of 4T1 cells with different treatments. Scale bar in (r), 50 μm. (t) Cell morphological TEM images of 4T1 cells. Red and blue arrows, respectively, indicate the positions of mitochondria and the chromatin of the nucleus (N, nucleus; C, cytoplasm). Scale bar, 500 nm. Statistical significance was determined by one-way ANOVA with Tukey's *post-hoc* test (a, b, h, i, j) or two-tailed Student's *t*-test (c, n, p).

Before the *in vitro* cellular experiments, bare ZnDHT was further modified with highly bio-friendly and water-soluble quaternary ammonium chitosan (CS) for biocompatibility and colloidal stability refinement (ZnDHT NM) (Fig. 4a and Fig. S23) [46,47]. The decoration of CS was demonstrated to negligibly affect its original structure and functions of ZnDHT but significantly improves the nanoparticle dispersion stability (Figs S23–S25). ZnDHT NM presents high stability serum proteins (Fig. S25). Utilizing the highly invasive triple-negative breast cancer (4T1) as a cell model, ZnDHT NM was evidenced to be increasingly internalized by the tumor cells over time, and mainly located in the lysosomes (Figs S26 and S27). During the process, ZnDHT NM not only chelated the Fe ions in the medium, but also consumed the intracellular Fe ions, indicating the capability of ZnDHT NM in depleting both extracellular and intracellular Fe ions in the tumor microenvironment (Figs S28 and S29). Moreover, the nanostructure-conversion of ZnDHT NM into ZnFeDHT NM (Fig. S30) and the markedly induced ROS elevation (Fig. 5d) were observed within the tumor cells, indicative of the Fe chelating and catalytic functioning of nanoparticles at the cellular level.

The anti-CSCs activity of ZnDHT NM was evaluated using 3D tumorsphere assays [48]. Elevated expressions of the breast CSC surface markers (CD44 and CD133) and stemness-related transcription factors (c-Myc, Sox2, Oct4, and Nanog) were identified in the 4T1 mammosphere cells relative to the adherent cells (Fig. S31), manifesting the efficient enrichment of CSC subpopulations in the tumorspheres [15,49]. These mammospheres were further utilized for experimental purposes. The mammosphere cells showed higher transferrin receptor (TfR) expression than the adherent cells, due to the enhanced Fe dependence of CSCs [20], and as expected, ZnDHT NM treatment further elevated the TfR level of mammosphere cells, corresponding to the intracellular Fe-absent characteristic (Fig. 4b) [23]. We carried out tumorsphere-forming experiments to estimate the inhibition effects on the CSC self-renewal potential. Treatment with ZnDHT NM noticeably reduced the size and number of formed tumorspheres in a dose-dependent manner (Fig. 4c). In addition, cytotoxicity and live/dead cell staining assays confirm a potent repression effect of ZnDHT NM against mammosphere cells (Fig. S32). A marked decline in the proportion of the aldehyde dehydrogenase (ALDH, another typical CSC biomarker [50]) highly expressed cell population sorted from mammosphere cells was determined after incubation with ZnDHT NM (Fig. 4d and Fig. S33a). ZnDHT NM also suppressed the CSC stemness as evidenced by a significant downregulation in the expression of Nanog, Sox2, and Oct4 genes and proteins (Fig. 4e and Fig. S33b–d). Furthermore, in the *in vivo* tumorigenesis inhibition estimation, the ZnDHT NM-treated mammosphere cells exhibited relatively lower tumorigenicity than that without nanomedicine treatment, as proved by the confined tumor formation in the mice for 21-d post inoculation (Fig. 4f and Fig. S34). Therefore, ZnDHT NM can effectively weaken the breast CSC traits and confine the tumor-initiating CSC pool.

Further, whole RNA-seq was performed to uncover the potential tumor cell regulatory mechanisms of ZnDHT NM (Figs S35 and S36). We identified the substantial differentially expressed (DE) mRNAs by treating with ZnDHT NM (Fig. 4g). According to the *Kyoto Encyclopedia of Genes and Genomes* (KEGG) enrichment analysis, the Wnt signaling pathway is among the top downregulated pathways in ZnDHT NM-treated cells (Fig. 4h), and the corresponding Protein-Protein Interaction (PPI) network based on STRING database underlines *β*-catenin (*Ctnnb1*) as a key target by therapeutic ZnDHT NM (Fig. 4i). The downregulation of *Ctnnb1* expression was also validated by RT-qPCR (Fig. 4j). Previous investigations have claimed that the Wnt/*β*-catenin signaling pathway as a vital regulator participates in EMT, CSC maintenance, tumor migration and metastasis, and can be positively modulated by Fe [51–53]. Fe chelation can therefore inhibit the Wnt signaling by destabilizing *β*-catenin [53]. Accordingly, we introduced transferrin (TF, Holo) which can release the delivered Fe^3+^ within cells as a cellular Fe supplement to reveal the concrete impacts of ZnDHT NM-induced Fe depletion on suppressing Wnt signaling. Western blot assays show that ZnDHT NM treatment has effectively blocked the Wnt signaling targets, while TF addition caused a noticeable retracement of such regulatory effects (Fig. 4k). Furthermore, simultaneous incubations with both ZnDHT NM and TF have proven the much weakened intrinsic inhibitory effects of ZnDHT NM on the EMT (downregulation of mesenchymal markers (Fibronectin-1 (FN1), Vimentin, and N-cadherin) and upregulation of epithelial marker (E-cadherin)) and CSC activities (reduced expression of CD44 and Sox2 proteins) (Fig. 4l). These evidences suggest that ZnDHT NM could induce CSC differentiation through Fe depletion-mediated Wnt signaling repression. In addition, gene ontology (GO) enrichment analysis indicates that, besides the Fe-related mechanisms, ZnDHT NM treatment also led to a significant variation in the biological processes in response to oxidative stress (Fig. 4m), which could be attributed to the induced ROS generation within tumor cells. The Western blot results show that ZnDHT NM upregulated the expression of FoxO3 protein by ROS production (Fig. 4n), which has been recognized as a critical manipulator in ROS promoting stem cell differentiation [29,54–56]. From this perspective, ZnDHT NM could activate FoxO3 through inducing intracellular ROS generation to promote CSC differentiation. We further observed that the addition of *N*-acetyl-L-cysteine (NAC, a ROS scavenger) impaired the capacities of ZnDHT NM in blocking the EMT and promoting phenotype differentiation (Fig. S37), offering further ROS-regulating evidence of ZnDHT NM in facilitating the differentiation of CSCs and reducing the CSC fraction.

We also inspected the anti-CSCs outcomes of DOX and DFOM, and found that both treatments with DOX and DFOM caused the dose-dependent suppressive effects on tumorsphere formation and the viability of 4T1 mammosphere cells (Fig. S38). Nevertheless, DOX partially scaled up the ALDH^high^ cell population and promoted the expressions of Sox2, Oct4, and Nanog genes and proteins (Fig. 4o and p, and Fig. S39), due to the high drug resistance of CSCs resulting in its non-CSCs–preferred cytotoxicity as well as its effect of increasing stemness-related performances [16,57,58]. As predicted, DFOM capable of Fe depletion decreased the percentage of ALDH^high^ cells and suppressed the CSC stemness as well. In contrast to DFOM, ZnDHT NM possesses a higher anti-CSC effectiveness, presumably attributing to its dual functions of Fe interference and catalytic ROS generation which collaboratively inhibit the CSC stemness. Additionally, the ZnDHT NM-treated tumor cells presented the most significantly deteriorated EMT, migration, and invasion capacities (Fig. S40).

### *In vitro* killing effect against tumor cells

The cytotoxicity of ZnDHT NM was estimated on different cell lines under general adherent cultures. ZnDHT NM caused a dose-dependent toxic effect on both 4T1 breast tumor cells and HCT-116 colon tumor cells, while no marked decline on cellular viability was observed for the treated normal cells (HDF, HEK-293T, NIH 3T3, HUVEC, and lymphocyte) (Fig. 5a and b, and Fig. S41). Moreover, under the acidified H_2_O_2_ condition (AHC) that simulates the tumor microenvironment [38], the viabilities of 4T1 and HCT-116 tumor cells treated with ZnDHT NM were lower than that cultured under neutral pH condition without H_2_O_2_ addition (Fig. 5c and Fig. S41). Live/dead cell staining assays provide consistent results with the above cytotoxicity tests (Fig. S42), convincingly revealing the anti-cancer specificity of ZnDHT NM that benefits biosafe therapy. DFOM and DOX displayed discrepant antiproliferative activities against the cells from each other, with the former being less toxic to both tumor and normal cells than the latter, whereas neither show highly significant tumor cell-selective inhibition effects (Fig. S43).

Based on the above findings, we hypothesize that the tumor-specific therapeutic outcome of ZnDHT NM stems from its mediated ROS generation specifically in the tumor microenvironment. To this end, we examined intracellular ROS production by utilizing a fluorescent ROS probe. The largely elevated intracellular ROS level was detected for the ZnDHT NM-treated 4T1 tumor cells but no noticeable ROS could be found generating in the normal cells after the same treatments (Fig. 5d, and Fig. S44a, b, and d). Also, AHC simulation was found to exacerbate ZnDHT NM-induced ROS generation within the tumor cells (Fig. 5e, and Fig. S44c and e). It has been reported that ROS production-mediated cell death is closely related to intracellular lysosomal membrane permeabilization (LMP) and mitochondria dysfunction [59]. The 4T1 cells treated with ZnDHT NM displayed obvious LMP similar to the chloroquine treatment (Fig. 5f) [23]. Meanwhile, the mitochondrial integrity evaluation demonstrates that ZnDHT NM caused a significant mitochondria membrane damage-associated potential alteration in the 4T1 cells and the AHC simulation further enhanced the mitochondria damage effect of ZnDHT NM (Fig. 5g and Fig. S45). On the other hand, the increased Zn^2+^ concentration in a dose- and time-dependent manner was detected in the tumor cells after incubation with ZnDHT NM (Fig. S46), as a result of the Fe^3+^-triggered structure transformation of ZnDHT NM and resultant release of Zn^2+^. It has been reported that Zn^2+^ can disturb the antioxidant defense of tumor cells, e.g. inactivating the GR in catalyzing the conversion of oxidized glutathione (GSSH) into reduced glutathione (GSH) (Fig. S47) [60,61]. ZnDHT NM was confirmed to inhibit the GR activity of 4T1 cells as well as reduce the intracellular GSH level (Fig. 5h and i), which would augment the ROS-caused cellular oxidative damage. Furthermore, ROS scavenger addition in tumor cells substantially prevented them from ZnDHT NM-induced death (Fig. S48a and b). Regardless of its robust Fe-depleting activity, DFOM also prevented ZnDHT NM from killing most of the treated tumor cells by significantly inhibiting the intracellular ROS production mediated by ZnDHT NM (Fig. S48c and d), implying that the major lethal effect of ZnDHT NM came from Fe-induced cellular ROS production, i.e. oxidative stress, rather than the simple deprivation of Fe alone. Together, ZnDHT NM can selectively kill tumor cells primarily resulting from its specific tumor microenvironment-dependent catalytic activity for ROS production.

Given that both ROS production and GSH reduction can induce distinct tumor cell death pathways [62,63], we then explored the specific death pathways in the ZnDHT NM-treated 4T1 cells. ZnDHT NM-induced cell death could be potently compromised by the apoptosis and ferroptosis inhibitors (Z-VAD-FMK and ferrostatin-1) but hardly intercepted by the necrosis and autophagy inhibitors (necrostatin-1 and 3-methyladenine) (Fig. 5j), indicative of the apoptosis and ferroptosis responsible for as-induced cell death. KEGG enrichment analysis of the DE mRNAs that responded to oxidative stress also reveals that ZnDHT NM could simultaneously activate the apoptosis and ferroptosis pathways in tumor cells (Fig. 5k). Moreover, PPI network investigations identify that Bcl-2 and TfR are the hub targets of ZnDHT NM in regulating tumor cell apoptosis and ferroptosis, respectively (Fig. 5l and m). Further, the remarkable downregulation of Bcl-2 expression and upregulation of Bax and cleaved caspase-3 expression were determined in the ZnDHT NM-treated tumor cells (Fig. 5n and o, and Fig. S49), further evidencing the activation of the apoptotic pathway. A typical Annexin V-FITC/propidium iodide (PI) staining experiment presents that ZnDHT NM induced the apoptosis of tumor cells with both dose-dependence and simulated tumor microenvironment-responsive ability (Fig. 5s and Fig. S50). Additionally, consumption of GSH can disable intracellular glutathione peroxidase 4 (GPX4), and combine with ROS generation to lead to irreversible lipid peroxidation (LPO), as a hallmark of ferroptosis [27]. As expected, ZnDHT NM treatment obviously promoted the occurrence of cellular LPO, which was further consolidated under the AHC (Fig. 5r and Fig. S51). The expression variations of the specific genes (*TfR, Chac1*, and *Ptgs2*) and proteins (GPX4 and TfR) were determined in the ZnDHT NM-treated 4T1 cells, corresponding to the features of ferroptosis activation (Fig. 5p and q, and Fig. S52) [64]. Moreover, the observable characteristics of apoptosis and ferroptosis, such as evident mitochondria shrinkage and chromatin condensation, were found in the tumor cells after incubation with ZnDHT NM (Fig. 5t). Collectively, these results illustrate that ZnDHT NM capable of ROS-generating and GSH-depleting capabilities would activate both apoptosis and ferroptosis to effectively cause the death of bulk tumor cells.

### Suppression of tumor growth, recurrence, and metastasis *in vivo*

First, the biosafety of ZnDHT NM was investigated in detail in healthy mice post systemic administration of ZnDHT NM. Neither body weight loss nor organ lesion and inflammation were found in these treated mice (Fig. S53). Compared to the control group, no meaningful variations of the liver and kidney function indexes and blood-related parameters were observed in the ZnDHT NM-administered mice (Fig. S54a–e). In addition, ZnDHT NM exhibited a negligible hemolytic effect (Fig. S54f).

**Figure 6.**
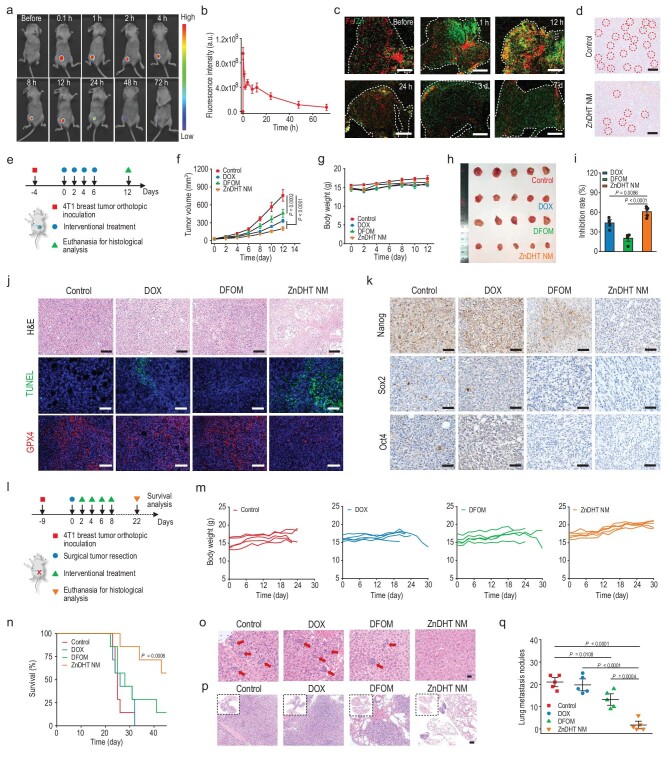
*In vivo* therapeutic efficacy evaluation on inhibiting orthotopic tumor growth and postoperative tumor recurrence and metastasis. (a, b) *In vivo* animal fluorescence images (a) and corresponding intensities (b) of the tumor-bearing mice post interventional treatment of Cy5-labeled ZnDHT NM (*n* = 3). (c) X-ray fluorescence (XRF) elemental imaging of Zn/Fe in the tumors after interventional treatment of ZnDHT NM. Scale bar, 1 mm. (d) Prussian blue staining of the tumors with or without ZnDHT NM interventional treatment. The blue spots with red circles represent iron staining in the tumors. Scale bar, 100 μm. (e) Illustration of experimental protocols for interventional treatments on orthotopic 4T1 breast tumor-bearing mice. (f, g) Tumor growth curves (f) and body weight variations (g) of the mice during treatments (*n* = 5). (h) Photographs of the obtained tumors after treatments. (i) Inhibitory rates of different treatments (*n* = 5). (j, k) Representative images of H&E staining, TUNEL assay, GPX4 immunofluorescence staining (j), and immunohistochemical protein analysis (k) of the obtained tumors after treatments. Scale bar in H&E and GPX4 staining, 100 μm; scale bar in TUNEL assay, 50 μm. Scale bar in (k), 50 μm. (l) Illustration of experimental protocols for interventional treatment in mice after surgical orthotopic 4T1 breast tumor resection. (m, n) Individual body weight variation (*n* = 5) (m) and survival curves (*n* = 7) (n) of the surgically treated 4T1 tumor-bearing mice post different interventional treatments, which were recorded in the parallel experiments. (o, p) Representative images of H&E staining of the livers (o) and lungs (p) obtained on day 22 post interventional treatments. Red arrows in (o) represent metastatic tumors in the livers. The inset images in (p) show the entire lung tissues. Scale bar in (o), 20 μm; scale bar in (p), 100 μm. (q) The counted numbers of lung metastasis nodules on day 22 post interventional treatments (*n* = 5). Statistical significance was determined by two-way ANOVA with Bonferroni's *post-hoc* test (f), one-way ANOVA with Tukey's *post-hoc* test (i, q), or log-rank (Mantel–Cox) test (n).

We next examined the *in vivo* anti-tumor potential of ZnDHT NM by establishing the orthotopic triple-negative 4T1 breast tumor mouse model due to its high malignancy, invasiveness, and rich CSC populations. *In vivo* fluorescence imaging results show that ZnDHT NM can effectively accumulate in the tumor lesions 24-h post interventional treatment, followed by subsequent gradual metabolization by the body (Fig. 6a and b, and Fig. S55). Compared to DOX and DFOM, the short-term (12 h) treatment of ZnDHT NM led to a most marked ROS elevation in the tumor tissue (Fig. S56). During 1-week treatment period, ZnDHT NM reduced the Fe levels of tumors substantially, as corroborated by the Zn accumulation and Fe exhaustion observed in the tumor tissues of ZnDHT NM-treated mice (Fig 6c and d). Further, the tumor growths of the mice after different interventional treatments were monitored for 12 d (Fig 6e). ZnDHT NM exhibited a noteworthy tumor-suppressing effect better than the DOX treatment at a clinically relevant dose, while DFOM only caused a moderate inhibition on the tumor growth (Fig. 6f, h, and i, and Fig. S57). All of these treatments did not cause visible body weight loss in the treated mice (Fig. 6g). Histological analysis presents extensive cell death in both the DOX and ZnDHT NM groups (Fig. 6j). However, only the ZnDHT NM-treated mice were manifested to show the coexistence of efficient cell apoptosis and ferroptosis in the tumor tissues as verified by the TUNEL assay and GPX4 immunofluorescence staining (Fig. 6j). To assess the *in vivo* stemness-inhibiting effects, we implemented the immunohistochemical and immunofluorescent analyses of CSC-associated protein expressions in the tumor tissues obtained from the different treated mice. More noticeable reduction in the Nanog, Sox2, Oct4, and CD44 expressions were found after administrating ZnDHT NM compared to the DFOM treatment, while DOX did not present any effect on suppressing CSCs (Fig. 6k and Fig. S58), in accordance with the *in vitro* anti-CSC outcome examinations. Surprisingly, metastatic tumors were detectable in the livers and lungs of mice after the 4T1 breast tumor orthotopic inoculation for 12 d, yet the ZnDHT NM treatment showed effectively suppressed liver and lung metastasis of the tumor (Fig. S59). These findings confirm that ZnDHT NM could synchronously kill bulk tumor cells and CSCs, and inhibit primary tumor growth and its distant metastasis, owing to its marked capacities of Fe interference and associated induction of oxidative damage.

To demonstrate the actual clinical application potential, we also estimated whether such a nanomedicine with the CSC-inhibiting function could potently inhibit postoperative tumor recurrence and metastasis on the surgically treated luciferase-labeled 4T1 tumor-bearing mice (Fig. 6l and Fig. S63a). The *in vivo* bioluminescence monitoring and histological analysis results indicate that both interventional and systemic treatments with ZnDHT NM could suppress the local regrowth and distant metastasis of tumors significantly compared to the control group, whereas the DOX and DFOM administrations were much less effective in inhibiting tumor regrowth and metastasis after surgical tumor resection (Fig. 6o–q and Figs S60–S65). Thereinto, the ZnDHT NM-treated group shows the lowest liver and lung metastasis rates compared to other treated groups. Moreover, according to the immunofluorescence co-staining of CD44/Sox2 stemness-associated proteins in the liver and lung tissues that can indicate metastatic tumor cells with high tumorigenicity and aggressiveness, it was found that ZnDHT NM treatment obviously shrank the liver and lung metastatic foci and inhibited tumorigenic and invasive potential (Figs S62 and S65). The therapeutic efficacy of interventional treatment was higher than that of systemic treatment, due to the fact that the former approach enables localized delivery of the therapeutic agent. From the long-term monitoring results, it can be seen that ZnDHT NM treatment has efficaciously alleviated the tumor-induced body weight losses on the surgical mice and prominently prolonged their survival (Fig. 6m and n, and Fig. S66). However, no significant survival extension was observed for the surgically treated tumor-bearing mice with the DOX or DFOM administration compared to the control group. From these data, ZnDHT NM possesses the encouraging capacity to inhibit the postoperative recurrence and metastasis of highly malignant triple-negative breast cancer. Such a simple and biocompatible nanoformulation with encouraging therapeutic effectiveness is believed to be highly promising for clinical cancer treatments.

## CONCLUSIONS

In summary, we have developed a coordination nanomedicine by DHT complexing Zn^2+^, which can be activated by the tumor microenvironmental Fe^3+^ for initiating the intractable CSCs-rooted tumor therapy. The Fe^3+^-selective capturing and *in situ* formed redox-active Fe-DHT structure from ZnDHT NM enable the depletion of endogenous Fe^3+^, the release of inherent Zn^2+^ to reduce endogenous GSH, and catalyze remarkable tumor-specific ROS production in an almost simultaneous manner. ZnDHT NM has been demonstrated to efficaciously suppress the EMT and CSC stemness by blocking the Wnt signaling pathway and activating FoxO3, and more importantly, is able to selectively kill tumor cells through inducing both apoptosis and ferroptosis. Ultimately, such a designed nanomedicine capable of concurrently extinguishing CSCs and bulk tumor cells demonstrates efficiently inhibited tumor growth, recurrence, and metastasis in the difficult-to-treat CSCs-enriched breast tumor models. Actually, receiving ZnDHT NM treatment *in vivo* still could not fully eliminate the tumors and completely avoid suffering from recurrence and metastasis, which could be attributed to the insufficient CSC/tumor cell-targeting delivery capacity of ZnDHT NM without modification, the insufficiency to induce explosive tumor oxidative damage owing to the limited available Fe content in tumors, the intricate endogenous mechanisms (e.g. Fe depletion probably promoting tumor angiogenesis), etc. Thus, corresponding improvements can be made in the future to further boost treatment efficiency, such as surface targeting modification, improving ROS-generating capacity, etc. Nevertheless, this study presents an innovative perspective of establishing biosafe nanomedicines to evoke effective therapeutic mechanisms against CSCs and bulk tumor cells by making use of endogenous substances, which is highly encouraging for cancer nanomedicine design and future tumor therapeutics.

## MATERIALS AND METHODS

Detailed materials and methods are available in the supplementary data files.

## ETHICAL STATEMENTS

This work was performed in accordance with the recommendations in the *Guide for the Care and Use of Laboratory Animals* and relevant Chinese laws and regulations. All the animal assays were approved by the Institutional Animal Care and Use Committees of Shanghai Tenth People's Hospital (approval number: SHDSYY-2022-6290).

## Supplementary Material

nwae434_Supplemental_File
