# Supplementary material for: Cascade specific endogenous Fe3+ interference and in situ catalysis for tumor therapy with stemness suppression
Source: Natl Sci Rev. 2024 Nov 29;12(2):nwae434. doi: 10.1093/nsr/nwae434 (PMC11833684; doi:10.1093/nsr/nwae434)
Supplement: nwae434_Supplemental_File [file nwae434_supplemental_file.pdf]

Supplementary information

Cascade specific endogenous Fe^{3+} interference and *in situ* catalysis for tumor therapy with stemness suppression

Jiajie Chen,[1,2,†] Yitong Wang,[3,†] Jian Huang,[6,†] Zhibo Yang,[1,2] Huicong Niu,[4] Xiaolian Su,[3] Jimin Huang,[1,2] Hongshi Ma,[1,2] Yufang Zhu,[1,2,*] Chengtie Wu,[1,2,*] and Jianlin Shi[1,2,5,*]

[1]State Key Laboratory of High Performance Ceramics and Superfine Microstructure, Shanghai Institute of Ceramics, Chinese Academy of Sciences, Shanghai 200050, China;

[2]Center of Materials Science and Optoelectronics Engineering, University of Chinese Academy of Sciences, Beijing 100049, China;

[3]Department of Radiology, Shanghai Tenth People's Hospital, School of Medicine, Tongji University, Shanghai 200072, China;

[4]Department of Neurology, Minhang Hospital, Fudan University, Shanghai 200032, China;

[5]Shanghai Frontiers Science Center of Nanocatalytic Medicine, Shanghai Tenth People's Hospital, School of Medicine, Tongji University, Shanghai 200331, China;

[6]Materials Genome Institute, Shanghai University, Shanghai 200444, China

***Corresponding authors.**

E-mails: zjf2412@163.com; chengtiewu@mail.sic.ac.cn; jlshi@mail.sic.ac.cn

[†]Equally contributed to this work.

Supplementary Materials and Methods

Chemicals and reagents

2,5-dihydroxyterephthalic acid (DHT), zinc acetate dihydrate ($Zn(CH_3COO)_2·2H_2O$), doxorubicin (DOX), and chloroquine phosphate were purchased from Aladdin Biochemical Technology Co., Ltd., Shanghai, China. Deferoxamine mesylate (DFOM) was provided by Macklin Biochemical Technology Co., Ltd., Shanghai, China. Quaternary ammonium chitosan (CS) was offered by Lvshen Bioengineering Co., Ltd., Nantong, China. 3,3',5,5'-tetramethylbenzidine (TMB) was bought from J&K Scientific (Beijing, China). Sodium acetate (NaAc), methylene blue (MB), rhodamine B (RhB), *N*,*N*-dimethylformamide (DMF), methanol (CH_3OH), ethanol (C_2H_5OH), dimethyl sulfoxide (DMSO), hydrochloric acid (HCl), and 30% hydrogen peroxide (H_2O_2) aqueous solution were purchased from Sinopharm Group Chemical Reagent Co., Ltd., China. Ferric chloride hexahydrate ($FeCl_3·6H_2O$), ferric nitrate nonahydrate ($Fe(NO_3)_3·9H_2O$), ferric sulfate hydrate ($Fe_2(SO_4)_3·xH_2O$), sodium sulfate (Na_2SO_4), *o*-phenylenediamine (OPD), *N*-acetyl-L-cysteine (NAC), fluorescein isothiocyanate (FITC)-dextran (FD10S, 10 kDa), reduced glutathione (GSH), and ferrostatin-1 were obtained from Sigma-Aldrich (Shanghai, China). Bovine serum albumin (BSA), superoxide dismutase (SOD), catalase (CAT), LysoTracker Green, 2’, 7’-dichlorofluorescin diacetate (DCFH-DA), Z-VAD-FMK, necrostatin-1, glutathione reductase (GR) assay kit with 5, 5’-dithio-bis(2-nitrobenzoic acid) (DTNB), GSH assay kit, Annexin V-FITC/propidium iodide (PI) apoptosis detection kit, and dihydroethidium (DHE) were provided by Beyotime Institute of Biotechnology (China). Mitochondrial membrane potential assay kit with JC-1 was purchased from Beijing Solarbio Science & Technology Co., Ltd. (China). Mouse transferrin receptor (TfR) ELISA kit was obtained from Shanghai Chutai Biotechnology Co., Ltd. Transferrin (TF, Holo) and 3-methyladenine were purchased from Yeasen Biotechnology Co., Ltd., Shanghai, China. Bicinchoninic acid (BCA) protein assay kit and BODIPY™ 581/591 C11 was provided by Thermo Fisher Scientific Technology Co., Ltd., USA. ROX reference dye and all the DNA strands (the sequences were listed in **Supplementary**

Table 8) were offered by Sangon Biotech Co., Ltd., Shanghai, China. All the antibodies used in this study were listed in **Supplementary Table 9**. Water purified with a Smart-Q15 laboratory water purification system was utilized throughout the work.

Characterization

Transmission electron microscopy (TEM) images, selected area electron diffraction (SAED) pattern, high-angle annular dark-field (HAADF) images, corresponding energy-dispersive spectroscopy (EDS) profiles, and element mappings were obtained from JEM-2100F electron microscope (JEOL, Japan). Scanning electron microscopy (SEM) images and corresponding EDS profiles were acquired on SU9000 electron microscope (Hitachi, Japan). The hydrodynamic sizes and zeta potentials of nanoparticles were determined utilizing Zetasizer Nano ZS90 (Malvern Instruments, UK). X-ray diffraction (XRD) patterns were measured on Ultima IV X-ray diffractometer (Rigaku, Japan). Fourier transform infrared (FTIR) spectra were recorded by Nicolet Is10 spectrometer (Thermo Scientific, USA). UV−vis absorption spectra were collected by an ultraviolet spectrophotometer (UV-2550, Shimadzu Corporation, Japan). The specific UV−vis absorption value and fluorescence intensity were collected by a microplate reader (Epoch, BIO-TEK, USA). X-ray photoelectron spectroscopy (XPS) spectra were obtained from K-Alpha instrument (Thermo Scientific, USA), and analyzed in XPSPEAK software (version 4.0). Raman spectra were acquired on in Via spectrometer (Renishaw, UK). The concentrations of different metal elements in solution were determined by an inductively coupled plasma mass spectrometry (ICP-MS, Agilent 7850, USA). Electron spin resonance (ESR) spectra were recorded on EMXplus-6/1 spectrometer (Bruker, Germany). The cell morphological TEM images were obtained from HT7800 electron microscope (Hitachi, Japan). Confocal laser scanning microscopy (CLSM) images were obtained on TCS SP8 confocal fluorescence microscope (Leica, Germany). Cells and tissue sections were observed under DM-i8 invert microscope (Leica, Germany). Flow cytometric assay was implemented utilizing SORP LSRFortessa flow cytometer (BD Biosciences, USA), and analyzed in FlowJo software (version 10.0.7). Western blotting results were

visualized with a Tanon 4800 imaging system (China).

Preparation of ZnDHT

ZnDHT was synthesized by a facile solvothermal approach. In brief, DHT (0.15 g) and $Zn(CH_3COO)_2{\cdot}2H_2O$ (0.25 g) were dissolved in *N*,*N*-dimethylformamide (DMF, 7.5 ml) containing H_2O (0.15 ml), respectively. After sufficient ultrasonic dissolution, the DHT solution was quickly reacted with the $Zn(CH_3COO)_2$ solution under magnetic stirring for 15 min. The yellow suspension was then transferred into a Teflon-lined stainless autoclave. After further reaction at 80 °C for 22 h, the products were obtained by high-speed centrifugation and then washed with DMF and ethanol. Finally, ZnDHT was stored in ethanol for further usage. Similarly, the ZnDHT with different diameters were prepared by adjusting the H_2O volume content (H_2O/DMF = 0, 0.02, 0.04, 0.08, and 0.16) in the reaction solvent.

Analysis of Fe^{3+}-specific capturing capacity

To check the response of ZnDHT toward different nutritional metal ions, the ion aqueous solutions (for example, KCl, NaCl, $CaCl_2$, $MgCl_2$, $ZnCl_2$, $Co(NO_3)_2$, $CuCl_2$, $MnCl_2$, $FeSO_4$, and $FeCl_3$; Sigma-Aldrich) at the concentration of 400 μM were first prepared, and then treated with the ZnDHT (80 μg/mL). In addition, various iron salts ($FeCl_3$, $Fe(NO_3)_3$, and $Fe_2(SO_4)_3$) and TF (Holo) with same concentration were also prepared for comparisons. Finally, the UV−vis absorption spectra of the mixed liquids were recorded after treatment for 1 h. The precipitates were obtained by centrifugation for further structural analysis. In addition, the $FeCl_3$ solutions with the different concentrations (0.1, 1, 5, 25, and 50 mM) were treated with the ZnDHT (1 mg/mL, after which the supernatants obtained by centrifugation were used for metal element quantitative analysis while the precipitates were collected for further morphological observation and structural analysis. To compare the Fe^{3+} chelation behavior of DHT molecule and ZnDHT, the $FeCl_3$ solution (400 μM) was treated with the ZnDHT (80 μg/mL) or the DHT (196.39 μM, the same dosage as in ZnDHT) in ethanol before recording the characteristic UV−vis absorption at different time

intervals.

X-ray absorption fine structure (XAFS) measurement and analysis

XAFS test, including extended X-ray absorption fine structure (EXAFS) and X-ray absorption near-edge structure (XANES), was carried out to characterize the coordination structure and chemical speciation of Fe element in the ZnFeDHT. The Fe K-edge measurement was performed with Si (111) crystal monochromators at the BL11B beamlines at the Shanghai Synchrotron Radiation Facility (SSRF) (China). Before the measurement at the beamline, samples were pressed into thin sheets with 1 cm in diameter and sealed using Kapton tape film. The XAFS spectra were recorded at room temperature using a 4-channel Silicon Drift Detector (SDD) Bruker 5040. The Fe K-edge EXAFS spectra were recorded in transmission mode. Negligible changes in the line-shape and peak position of Fe K-edge XANES spectra were observed between two scans taken for a specific sample. The obtained XAFS data was processed in Athena software (version 0.9.26) for background, pre-edge line and post-edge line calibrations. Moreover, Fourier transformed fitting was conducted to acquire the quantitative structural parameters in Artemis software (version 0.9.26) [1,2]. Furthermore, for wavelet transform analysis, the $\chi(k)$ exported from Athena was imported into the Hama Fortran code. The parameters were listed as follow: R range, 1.0 - 6.0 Å; k range, 0 - 10.0 $Å^{-1}$; k weight, 3; and Morlet function with $\kappa = 15$, $\sigma = 1$ was used as the mother wavelet to offer the overall distribution. The authors would like to thank Shiyanjia Lab (www.shiyanjia.com) for the support of XAFS test.

Electrochemical measurements

A three-electrode cell with a glassy carbon electrode as the working electrode, an Ag/AgCl electrode (saturated with KCl) as the reference electrode, and a platinum electrode as the counter electrode, was utilized for conducting cyclic voltammetry (CV) test, based on Autolab 302N electrochemical workstation (Metrohm, Germany). The CV curve was recorded between -1.0 and +1.5 V in the Na_2SO_4 electrolyte solution (1 M, pH 5.2) containing both DHT (50 mM) and Fe^{3+} (50 mM) with a scan

rate of 0.05 V/s. The CV measurement was originally swept from +0.1 to -1.0 V, followed by five cycles. In addition, the CV curves of the electrolyte solutions containing single DHT (50 mM) or Fe^{3+} (50 mM) were also recorded.

Detection of ROS production

Prior to each trial, fresh ZnFeDHT was prepared through mixing ZnDHT (80 μg/mL) with Fe^{3+} (400 μM) directly in the test solution, and the same concentration of free Fe^{3+} and ZnDHT were set for comparison.

ESR assay. For •OH detection, the ZnFeDHT in sodium acetate (NaAc) buffer solution (20 mM, pH 5.2) with or without H_2O_2 (10 mM) was mixed with the equal volume 5,5-dimethyl-1-pyrroline-*N*-oxide (DMPO) (100 mM in H_2O) acted as a spin-trapping agent. For $O_2•^-$ detection, the fresh ZnFeDHT prepared in methanol was mixed with the equal volume DMPO (100 mM in methanol).

TMB and MB colorimetric analyses. The colorless TMB can be oxidized by •OH into the blue TMB cation-free radical (oxTMB) showing a characteristic absorption at 648 nm. The blue MB can be oxidized by •OH to undergo color fading with a decline of the characteristic absorption at 665 nm. Typically, 10 μL TMB (40 mM in ethanol) or MB (5 mM in H_2O) as the substrate were added into the H_2O_2-contained NaAc buffer solution (20 mM, pH 5.2) in the presence of ZnFeDHT. The NaAc buffer solutions with different pH (6.0 and 7.4) were also prepared for comparison. The UV–vis absorption spectra of the test solutions were recorded at a certain reaction time.

OPD fluorescent analysis. The non-fluorescent OPD can be easily oxidized by ROS to form the yellow fluorescent 2, 3-diaminophenazine (DAP). 10 μL OPD (40 mM in ethanol) were added into the NaAc buffer solution (20 mM, pH 5.2) with different settings (blank; Fe^{3+}; ZnDHT; ZnFeDHT; H_2O_2; Fe^{3+} + H_2O_2; ZnDHT + H_2O_2; ZnFeDHT + H_2O_2). After reaction for 10 min, the test solutions were used for fluorescence intensity measurement (E_x: 417 nm, E_m: 557 nm). To remove the dissolved O_2, the solution was sufficiently bubbled with N_2 before reaction.

RhB decolorization assay. 4 μL RhB (5 mM in H_2O) were added into the different

groups (blank; Fe^{3+}; ZnDHT; ZnFeDHT; ZnFeDHT + SOD; ZnFeDHT + CAT) in NaAc buffer solution (20 mM, pH 5.2). The addition of SOD or CAT aimed to investigate the $O_2\bullet^-$ and H_2O_2 intermediate generations during the reactions, respectively. The characteristic UV−vis absorption values at 554 nm of the test solutions were recorded after 12 h of reaction for determining the relative RhB-decolorizing efficacies, according to the formula of $(A_{blank} - A_{sample})/ (A_{blank} - A_{ZnFeDHT}) \times 100\%$.

Calculation details

All the DFT calculations were performed utilizing the Gaussian 09 (G09) program package. The optimization was conducted employing Becke's three-parameter hybrid method using the LYP correlation functional (B3LYP) level. The B3LYP geometries were optimized based on the LANL2DZ basis sets. Relative energies of all stationary points were corrected with zero-point vibrational energies (ZPE). The molecular structure of DHT was built on the basis of the repeated fragments in the unit cell without considering H_2O molecule. The BDE was determined by the following equation:

$$E_{BDE} = E_{complex} - (E_{ion} + E_{DHT})$$

where $E_{complex}$ is the total energy of coordination complex, and E_{ion} and E_{DHT} are the energies for metal ion and DHT molecule. Thereinto, three different binding modes were carefully screened. Charge distribution analyses were performed on the basis of the Mulliken population. While for the Fe-DHT coordination complex, four DHT molecules are coordinated with one Fe^{3+}, and most stable coordination structure (i.e., hexacoordinated conformation) was fully optimized. Similarly, other hexacoordinated structures between different trivalent metal ions and DHT molecules were also analyzed. Furthermore, the hexacoordinated Fe-DHT structure was used to calculate the proposed catalytic reactions. Transition states were evaluated utilizing the intrinsic reaction coordinate (IRC) method at the B3LYP level. Gibbs free energies were further estimated following the multiple-step reaction in order to compare the energy barriers between Fe^{3+} and Fe-DHT.

Preparation of ZnDHT NM

For biological applications, ZnDHT was further modified by CS to construct ZnDHT NM. In detail, the obtained ZnDHT was resuspended in H_2O (1 mg/mL), and subsequently mixed with quaternary ammonium CS aqueous solution (2 mg/mL) in a glass vial under ultrasonic oscillation at room temperature for 30 min. Afterwards, the CS-decorated ZnDHT NM was centrifugally collected and washed with H_2O for three times. Finally, ZnDHT NM was stored at 4 °C until further usage, and the dosage used was determined based on the bare ZnDHT content. To evaluate the stability against serum proteins, ZnDHT NM was treated with bovine serum albumin (10 mg/mL) and the particle size variations were monitored at different time points.

Cell culture

4T1 cells labeled with luciferase were obtained from Y. Wang at Shanghai Tenth People's Hospital. HCT-116 cells were provided by Shanghai Yuchun Biology Co., Ltd. Human dermal fibroblasts (HDF), mouse embryonic fibroblasts (NIH 3T3), human embryonic kidney cells (HEK-293T), and human umbilical vein endothelial cells (HUVEC) were acquired from the Cell Bank of Chinese Academy of Sciences. Lymphocytes were collected from the mouse peripheral blood by using a mouse peripheral blood lymphocyte separation kit (Beyotime) based on the principle of density gradient centrifugation. 4T1, HDF, NIH 3T3, and HEK-293T cells were cultivated in the Dulbecco's modified Eagle medium (DMEM) (Gibco, USA) supplemented with 10% (v:v) fetal bovine serum (FBS) and 1% (v:v) penicillin-streptomycin (P/S). HCT-116 cells were cultivated in the specific complete medium (CM-0096, Yuchun Biology, Shanghai). HUVEC cells were cultivated in the endothelial cell medium (ScienCell, USA) with the addition of 5% (v:v) FBS, 1% (v:v) endothelial cell growth factor, and 1% (v:v) P/S. Lymphocytes were cultivated in the RPMI 1640 medium (Gibco, USA) supplemented with 10% (v:v) FBS and 1% (v:v) P/S. For CSC tumorsphere culture, 4T1 cells were seeded in ultralow attachment culture dishes (Corning, USA) with the serum-free DMEM/F12 culture medium (Gibco, USA) containing B27 supplements (1×), epidermal growth factor (EGF, 20 ng/mL), basic

fibroblast growth factor (bFGF, 20 ng/mL), insulin (5 μg/mL), and 1% (v:v) P/S to acquire the 4T1 mammosphere cells. All the cultures were conducted in an incubator (Thermo Scientific, USA) at 37°C and 5% CO_2 atmosphere.

Cellular uptake

4T1 cells were seeded in CLSM-exclusive culture disk and cultured for 24 h. Subsequently, the cells were incubated with the fresh culture medium containing Cy5-labeled ZnDHT NM (40 μg/mL) for various time intervals (10 min, 1 h, and 5 h). Then, these cells were gently washed with PBS for three times before the fixation with paraformaldehyde solution (4 wt%) for 10 min. After PBS washing, the cell nuclei were stained with DAPI for 5 min before fluorescence observation in CLSM. To determine the location of nanoparticles and lysosomes within cells, 4T1 cells were co-treated with Cy5-labeled ZnDHT NM (40 μg/mL) and LysoTracker Green at 37 °C for 1 h. After PBS washing, Hoechst 33342 was employed to stain the cell nuclei for 5 min before imaging by CLSM.

Analysis of intracellular Fe content

The intracellular Fe levels were monitored by an intracellular iron colorimetric assay kit (APPLYGEN, Beijing, China). 4T1 cells were seeded in adherent 6-well plates (1×10^5 cells per well) with the serum-containing culture medium for 24 h. Afterwards, the cells were incubated in the fresh culture medium containing free Fe^{3+} ($FeCl_3$, 100 μM) and ZnDHT NM (0 or 40 μg/mL) for 5 h. The cells without specific treatment were served as control. After the incubation, these cells were gently washed with cold PBS for two times. Then, the cell lysis reagent of assay kit was used to treat the cells in a table concentrator for 2 h. Finally, the cell lysate fluids were collected for iron colorimetric assay according to the manufacturer's protocol. Thereinto, the supernatants from the cell lysate fluids obtained by centrifugation (4 °C, 12000 rpm, 10 min) were used for intracellular free Fe ion analysis, while the cell lysate fluids without centrifugation treatment in the parallel experiments were directly used for intracellular total Fe content analysis.

ELISA analysis of TfR protein level

4T1 mammosphere cells were seeded in ultralow attachment 6-well plates (1 × 10^5 cells per well) with the serum-free CSC suspension culture medium, and incubated with ZnDHT NM (15 μg/mL) or DFOM (100 μM) for 48 h. The 4T1 mammosphere cells and adherent cells without treatment were also collected for comparisons. Afterwards, the cells were treated by RIPA lysis buffer for 1 h, and centrifuged (4 °C, 12000 rpm, 10 min) to obtain the supernatants. BCA protein assay was performed to determine the total protein content. Then, the TfR protein levels of different samples were analyzed using TfR ELISA kit according to the manufacturer's protocol.

Evaluation of anti-CSCs ability

Tumorsphere-forming assay. 4T1 mammosphere cells were seeded in ultralow attachment 6-well plates (1 × 10^5 cells per well) with the serum-free CSC suspension culture medium. After 24 h of culture, these cells were treated with different concentrations of DOX, DFOM, and ZnDHT NM for 4 days. After treatments, the formed tumorspheres were observed and imaged under an inverted microscope, and the number of tumorspheres (diameter > 50 μm) was counted.

Tumorsphere cytotoxicity assessment. 4T1 mammosphere cells were seeded in ultralow attachment 96-well plates (1 × 10^4 cells per well) with the serum-free CSC suspension culture medium for 24 h. Subsequently, the cells accepted with the treatments as described above for 48 h were further incubated with the cell counting kit-8 (CCK-8) for 6 h, and the final OD_{450} was recorded for cell viability analysis. In addition, the living/dead cells after treatment with different concentrations of ZnDHT NM were incubated with Calcein-AM/PI staining solution at 37°C for fluorescent observation.

Determination of ALDH^{high} proportion. To monitor the proportion of ALDH^{high} cells in tumorspheres, 4T1 mammosphere cells seeded in ultralow attachment 6-well plates (1 × 10^5 cells per well) were incubated with different concentrations of ZnDHT

NM or various formulations, DOX (0.5 μM), DFOM (100 μM), or ZnDHT NM (15 μg/mL) for 48 h. Then, the treated mammosphere cells were dissociated, and further stained with ALDEFLUOR™ kit (STEMCELL Technologies, 01700) according to the manufacturer's protocol for flow cytometer analysis.

Examination of stemness-associated protein and gene expressions. 4T1 mammosphere cells were seeded, incubated with various formulations as described above for 48 h. Afterwards, the total proteins and RNA of the treated mammosphere cells were extracted by RIPA lysis buffer (Beyotime, China) and TRIzol reagent (Invitrogen, USA), respectively. To examine the expression of proteins (for example, Sox2, Oct4, and Nanog) at the different groups, bicinchoninic acid (BCA) protein assay was firstly carried out to determine the total protein content, and then typical Western blot experiment was performed by electrophoresis run on denaturing polyacrylamide gels to visualize the comparable results. To estimate the relative gene levels (for example, Sox2, Oct4, and Nanog), the RNA content was firstly quantified by Nano-Drop spectrophotometry (Thermo Fisher, USA), and subsequently according to the manufacturer's protocol RNA was transcribed into cDNA by employing PrimeScript 1st Strand cDNA synthesis kit (TOYOBO, Japan) for further real-time quantitative polymerase chain reaction (RT-qPCR) process on a StepOnePlus Real time system (Applied Biosystems, USA). For RT-qPCR, Gapdh was utilized as the housekeeping gene and the relative gene expressions were calculated by the $2^{-\Delta\Delta Ct}$ method. All the used antibodies and primer sequences are described in the Supplementary information.

Bulk RNA-seq and data analysis

4T1 cells seeded in adherent 6-well plates (1×10^5 cells per well) were incubated in the fresh culture medium with or without ZnDHT NM (40 μg/mL) for 48 h. After the incubation, these cells were gently washed with PBS for two times, and then treated with TRIzol reagent to extract RNA. The samples were sequenced using Illumina NovaSeq6000 with the mode of PE150 (Pair-end 150 bp). Bulk RNA-seq analysis was performed by Shanghai Biotechnology Corporation. After DE mRNAs

screening (P value < 0.05, |FC| ≥ 1.5) [3], the KEGG pathway and GO enrichment analyses were conducted and R software (version 4.3.1) was applied for bioinformatic result plotting. Additionally, Cytoscape software (version 3.8.0) was used for analyzing the PPI network of genes based on STRING database (https://cn.string-db.org/) [4].

Cell migration and invasion assays

Tumor cell migration ability was evaluated by a wound-healing assay. In brief, 4T1 cells were seeded in adherent 12-well plates (5×10^4 cells per well) for 24 h. After sticking to the wall, the monolayer cells were scratched with three parallel vertical lines by a pipette tip, and then the detached cells were gently washed with PBS for three times. Afterwards, the cells were incubated in the culture medium with different formulations, DOX (1 μM), DFOM (200 μM), or ZnDHT NM (30 μg/mL). The cells without specific treatment were served as control. After 12 h of incubation, the cells were imaged under an inverted microscope and analyzed by ImageJ software.

Additionally, tumor cell invasion ability was assessed by a transwell assay. In brief, the 4T1 cells with different pretreatments (blank; DOX, 1 μM; DFOM, 200 μM; ZnDHT NM, 30 μg/mL), were seeded onto the upper chamber of transwells, which were soaked in the 24-well plates containing 500 μL of fresh culture medium in each well. After 12 h of incubation, the cells in the upper chamber were wiped off while the invaded cells on the lower surface were fixed with paraformaldehyde solution (4 wt %) for 20 min and further stained with 0.1% crystal violet for 10 min. The invaded cells were imaged and counted in the randomly selected areas.

Determination of tumor cell-killing effect

Cytotoxicity assessment. Different kinds of cells, including 4T1, HCT-116, HDF, NIH 3T3, HEK-293T, HUVEC, and lymphocyte cells, were seeded in adherent 96-well plates (1×10^4 cells per well) with the serum-containing culture medium for 24 h. Afterwards, the cells were incubated in the fresh culture medium containing different concentrations (0, 5, 10, 15, 20, 25, 30, 35, 40, 45, and 50 μg/mL) of ZnDHT

NM for another 24 h. In addition, the neutral culture medium (pH 7.4) without H_2O_2 as the normal physiological environment and the mildly acidic culture medium (pH 6.0) in the presence of H_2O_2 (100 μM) as the tumor microenvironment were regulated to culture the tumor cells for comparisons. Ultimately, the cell viabilities were determined by typical CCK-8 assay. Additionally, the cells after treatments were also characterized by the Calcein-AM/PI living/dead cell staining.

Intracellular ROS generation sensing. DCFH-DA probe can be converted into DCFH within cells, which is capable of reacting with ROS to produce DCF with green fluorescence (E_x: 488 nm, E_m: 520 nm). In detail, 4T1 and HDF cells were seeded in confocal laser scanning microscopy (CLSM)-exclusive culture disk, incubated with the fresh culture medium with or without ZnDHT NM (50 μg/mL) for 5 h. In addition, 4T1 cells were incubated with the regulated culture medium with or without ZnDHT NM (40 μg/mL) for 5 h. Next, the cells were gently washed with phosphate-buffered saline (PBS) twice, and further incubated with DCFH-DA (10 μM) at 37°C for 20 min and then with Hoechst 33342 for 5 min before fluorescence observation in CLSM and fluorescent intensity measurement by flow cytometry.

LMP assay. Intracellular LMP was evaluated by detecting the transfer of bulky FITC-dextran (10 kDa) from the lysosome into the cytosol. In detail, 4T1 cells were firstly incubated with the fresh culture medium containing FITC-dextran (1mg/mL) for 2 h. Then, these cells were gently washed with PBS and treated with ZnDHT NM (40 μg/mL) or chloroquine (100 μM, as positive control) for 5 h. Afterwards, the cells were fixed with paraformaldehyde solution (4 wt %) for 10 min. Finally, the cell nuclei were stained with 4′,6-diamidino-2-phenylindole (DAPI) for 5 min before fluorescence observation in CLSM.

Mitochondrial membrane potential analysis. Based on JC-1 probe, red fluorescence can be detected in normal mitochondrial membranes with high potential (JC-1 aggregate) while green fluorescence can be found in damaged mitochondrial membranes with low potential (JC-1 monomer). 4T1 cells accepted with the treatments as described above for 5 h. Next, the cells were incubated with JC-1 staining solution according to the manufacturer’s protocol before fluorescent

observation and imaging.

Apoptosis detection. 4T1 cells were seeded in adherent 6-well plates (1 × 10^5 cells per well) and accepted with the treatments as described above for 24 h. Afterwards, all of treated cells were trypsin-dissociated, washed, and treated with Annexin V-FITC/PI apoptosis detection kit in the dark at 37°C for flow cytometer analysis.

LPO assay. Cellular LPO was detected by using BODIPY™ 581/591 C11 as a fluorescent sensor. After different treatments for 5 h, 4T1 cells were incubated with BODIPY™ 581/591 C11 dye (10 μM) at 37 °C for 30 min. Then, the cell nuclei were stained with Hoechst 33342 for 5 min before fluorescence observation and imaging.

Intracellular Zn^{2+} ion release

4T1 cells were treated with different concentrations (0, 30, 40, and 50 μg/mL) of ZnDHT NM for 10 h or with ZnDHT NM (40 μg/mL) for different times (0 min, 10 min, 5 h, and 10 h). Next, the cells were gently washed with PBS and further incubated with a Zn^{2+} ion fluorescent probe, Metal Fluor™ Zn-520 (AAT Bioquest, USA), at 37°C according to the manufacturer's protocol. Finally, the cell nuclei were stained with Hoechst 33342 for 5 min before fluorescence observation and imaging.

Assessment of GR activity

4T1 cells were seeded in adherent 6-well plates (1 × 10^5 cells per well), and were incubated in the fresh culture medium with different concentrations of free Zn^{2+} ($ZnCl_2$, 0, 0.5, 1, 2, 4, and 8 μg/mL) or ZnDHT NM (0, 30, 40, and 50 μg/mL) for 5 h. After washing with PBS, the cells in different groups were collected and then were treated by RIPA lysis buffer at 4 °C. The supernatants were obtained by centrifugation (4 °C, 12000 rpm, 10 min) for GR activity evaluation using GR assay kit according to the manufacturer's protocol.

Analysis of intracellular GSH level

4T1 cells seeded in adherent 12-well plates (5 × 10^4 cells per well) were treated

with different concentrations of ZnDHT NM (0, 30, 40, and 50 μg/mL) for 5 h. After washing with PBS, the cells in different groups were collected and then were treated by Triton-X-100 buffer (0.4%) at 4 °C. The supernatants were obtained by centrifugation (4 °C, 12000 rpm, 5 min), and were mixed with 20 μL of DTNB/DMSO test solution (10 mM). Finally, the characteristic UV−vis absorbance values at 412 nm of the test solutions were recorded after 5 min of reaction for determining the relative GSH levels.

Animal experiment

The female BALB/c nude mice (4-week-old, about 15 g) used in the animal experiments were acquired from Shanghai Legen Biotechnology Co., Ltd. All the animal assays were approved by the Institutional Animal Care and Use Committees of Shanghai Tenth People’s Hospital (approval number: SHDSYY-2022-6290).

***In vivo* biosafety assessment**

Healthy BALB/c nude mice were systemically treated with ZnDHT NM (5, 10, and 20 mg/kg) through tail vein injection. The injection of saline alone was set as the control group. The body weights of these treated mice were recorded (n = 5). After one month of feeding, the systemic blood from mice was collected from the eyes and the serum was obtained by centrifugation for hematological and biochemical indicators analysis. In addition, the main organs (heart, liver, spleen, lung, and kidney) were extracted and further sliced up for histopathological evaluation by H&E staining.

Hemolysis analysis

Red blood cells (RBCs) were collected from the mouse blood for hemolysis analysis. The purified RBCs were incubated with different concentrations (25, 50, 100, 200, 400, and 800 μg/mL) of ZnDHT NM at 37 °C, and incubated with PBS and H_2O as negative and positive control, respectively. After 1h of incubation, the supernatants were obtained by centrifugation (3000 rpm, 10 min) and their characteristic UV−vis

absorption values at 540 nm were recorded for calculating the hemolysis rates on the basis of the formula of $(A_{sample} - A_{PBS})/(A_{water} - A_{PBS}) \times 100\%$.

Inhibition of *in vivo* tumorigenesis

4T1 mammosphere cells were incubated with the serum-free CSC suspension culture medium containing different concentrations of ZnDHT NM for 96 h. Then, the treated mammosphere cells were collected, dissociated, and injected into the mammary fat pads of female BALB/c nude mice (5×10^5 cells per mouse). After 21 d of feeding, the tumor volume and weight were recorded (n = 5).

***In vivo* fluorescence imaging**

Three 4T1 tumor-bearing mice were treated with Cy5-labeled ZnDHT NM (10 mg/kg) through intratumoral injection. The mice without treatment were served as control. Subsequently, the *in vivo* fluorescence images and the fluorescence intensities were obtained and recorded using the VISQUE InVivo Smart-LF system at predetermined time points (0.1 h, 1 h, 2 h, 4 h, 8 h, 12 h, 24 h, 48 h, and 72 h) (E_x: 650 nm; E_m: 670 nm).

Intratumoral ROS generation detection

To sense the ROS production in the tumor area of mice, the DHE staining assay was performed. The fresh tumor tissues of 4T1 tumor-bearing mice were collected at 12 h post different interventional treatments (saline; DOX, 2 mg/kg; DFOM, 4.86 mg/kg; ZnDHT NM, 10 mg/kg where DHT was 4.86 mg/kg), and further embedded in optimal cutting temperature (OCT) compound for frozen section (4 μm thickness). Subsequently, the tumor slices were incubated with DHE (20 μM) at 37 °C for 1 h and then stained with Hoechst 33342 for 10 min before fluorescence observation in CLSM.

X-ray fluorescence (XRF) elemental imaging and Prussian blue staining

To determine the tumor tissue distributions of Zn and Fe, XRF elemental imaging

was carried out on M4 Tornado Micro-XRF spectrometer with ESPRIT 1.6 (Bruker, Germany), provided by Boyue Instruments Co., Ltd., Shanghai, China. The fresh tumor tissues of 4T1 tumor-bearing mice were dissected at different time points (1 h, 12 h, 24 h, 3 d, and 7 d) post the treatment with ZnDHT NM (10 mg/kg) through intratumoral injection (the unexposed mice were served as control), and further embedded in OCT compound for frozen section (40 μm thickness). The tumor tissue slices were placed on a glass slide and kept at 4 °C for further XRF measurement. To monitor the tissue accumulation of Fe, the tumors of the 4T1 tumor-bearing mice with or without ZnDHT NM interventional treatment were dissected at 7 d, and further sliced up (4 μm thickness) for standard Prussian blue staining. The optical photographs were obtained by an invert microscope.

Assessment of *in vivo* anti-cancer efficiency

Inhibition of orthotopic breast tumor growth. To build the orthotopic 4T1 tumor models, 4T1 cells suspended in PBS were inoculated into the mammary fat pads of female BALB/c nude mice (1×10^6 cells per mouse). After 4 d, the 4T1 tumor-bearing mice with a tumor volume of about 30 mm^3 were assigned to four groups (n = 5) at random: 1) saline (as the control group); 2) DOX (2 mg/kg, a clinically relevant dose [5]); 3) DFOM (4.86 mg/kg); 4) ZnDHT NM (10 mg/kg, where DHT was 4.86 mg/kg). The 4T1 tumor-bearing mice were treated with different formulations once every two days for four times through intratumoral injection as interventional treatment. The tumor volumes ($width^2 \times length \times 0.5$) and the body weights of these treated mice were recorded every two days. After a period of 12 d, the tumors of mice were extracted for weighting (the tumor inhibition rate was calculated by $(1 - w/w_0) \times 100\%$; w, the tumor weights of the treatment groups; w_0, the tumor weight of the control group), and further sliced up for H&E staining, TUNEL assay, and GPX4 activity investigation as well as immunohistochemical/immunofluorescent analyses of CSC-associated protein expressions (Nanog, Sox2, Oct4, and CD44). In addition, the main organs (heart, liver, spleen, lung, and kidney) were also extracted for H&E staining.

Inhibition of postoperative breast tumor recurrence and metastasis. To build the orthotopic 4T1 tumor resection models, 4T1 cells suspended in PBS were inoculated into the mammary fat pads of female BALB/c nude mice (1×10^6 cells per mouse). After 9 d, the 4T1 tumor-bearing mice were surgically treated to remove the visible tumor tissues and the wound was carefully sutured and sterilized. After the wound healed on the Day 2, the surgically treated mice were intratumorally or intravenously injected with different formulations as described above once every two days for four times as interventional treatment or systemic treatment. The body weights of these treated mice were recorded every two days within 30 days, and the mice were intraperitoneally injected with D-Luciferin potassium salt at predetermined time points for monitoring tumor recurrence and metastasis by bioluminescent imaging. At the Day 22, a portion of treated mice in the parallel experiments were executed and the lung and liver tissues from each group were collected for H&E staining and CD44/Sox2 immunofluorescence co-staining analyses. For survival rate assessment, the survival periods of the surgically treated mice were recorded during different treatments until the Day 45.

Statistical analysis

For analysis of the collected data, Origin 2018 software, Microsoft Excel 2016 software, and GraphPad Prism 9.5.1 software were employed. ImageJ software was utilized to record the mean fluorescence intensities (MFI) in fluorescence images and count the regional areas and the cell numbers in optical photographs. The results obtained from independent experiments ($n \geq 3$) were expressed as means ± standard error of the mean (s.e.m.). The significance of the difference was determined *via* two-tailed Student's *t*-test for comparisons between two groups, through one-way analysis of variances (ANOVA) with Tukey's post-hoc test or two-way ANOVA with Bonferroni's post-hoc test for comparisons among multiple (more than two) groups, by log-rank (Mantel-Cox) test for the comparison of survival curves. The P values less than 0.05 were considered statistically significant.

Supplementary Figures

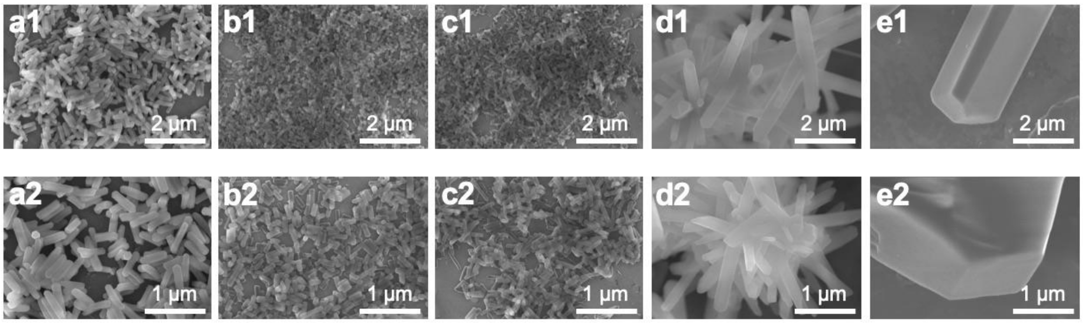

Figure S1. SEM images of ZnDHT with different diameters synthesized in the reaction solvents with the H_2O/DMF volume ratio of 0 (**a1**,**2**), 0.02 (**b1**,**2**), 0.04 (**c1**,**2**), 0.08 (**d1**,**2**), and 0.16 (**e1**,**2**). The size of ZnDHT can be regulated by varying the H_2O content in the reaction solvent due to that H_2O-dissociated protons can coordinate with the carboxyl groups of both DHT and CH_3COO^- or can affect the formed coordination bonds of Zn-deprotonated DHT during the competitive and reversible dissolution-recrystallization process in the solvothermal reaction [6]. All the materials show a clear rod-like shape. Without H_2O addition, the synthesized ZnDHT has a relatively large size (~130 nm in width and ~480 nm in length). Upon H_2O addition, the size of ZnDHT increases from nanometers to micrometers with the elevation of H_2O content. Given the anti-cancer application, the synthesized ZnDHT (**b1**,**2**) with a relatively small nanoscale size was selected for the further studies.

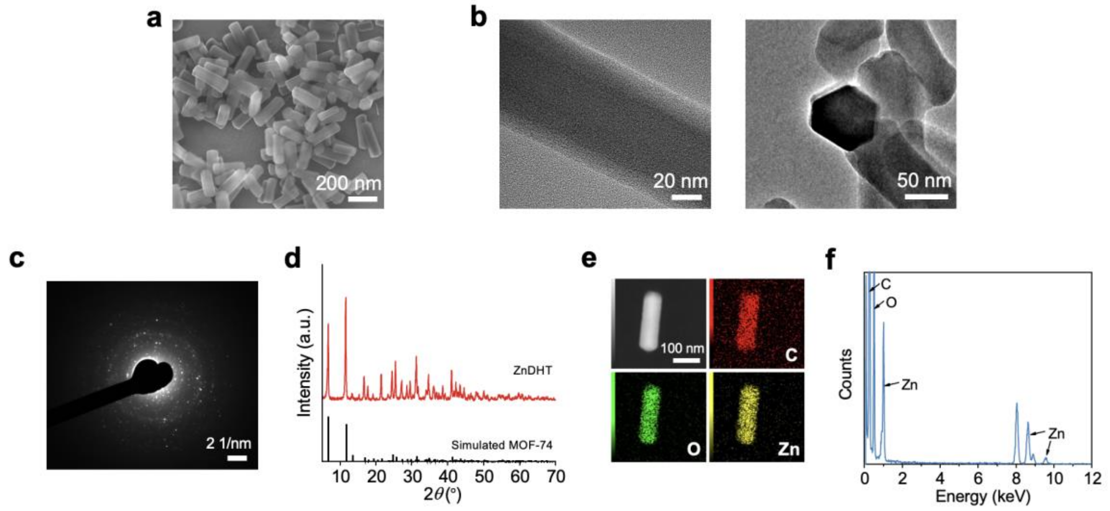

Figure S2. SEM image (**a**), TEM images (**b**), SAED pattern (**c**), XRD pattern (**d**), element mappings (**e**), and EDS profile (**f**) of as-selected nanosized ZnDHT in this work. The crystalline structure of synthesized ZnDHT is similar with the reported metal-organic framework (MOF)-74-type crystal [7].

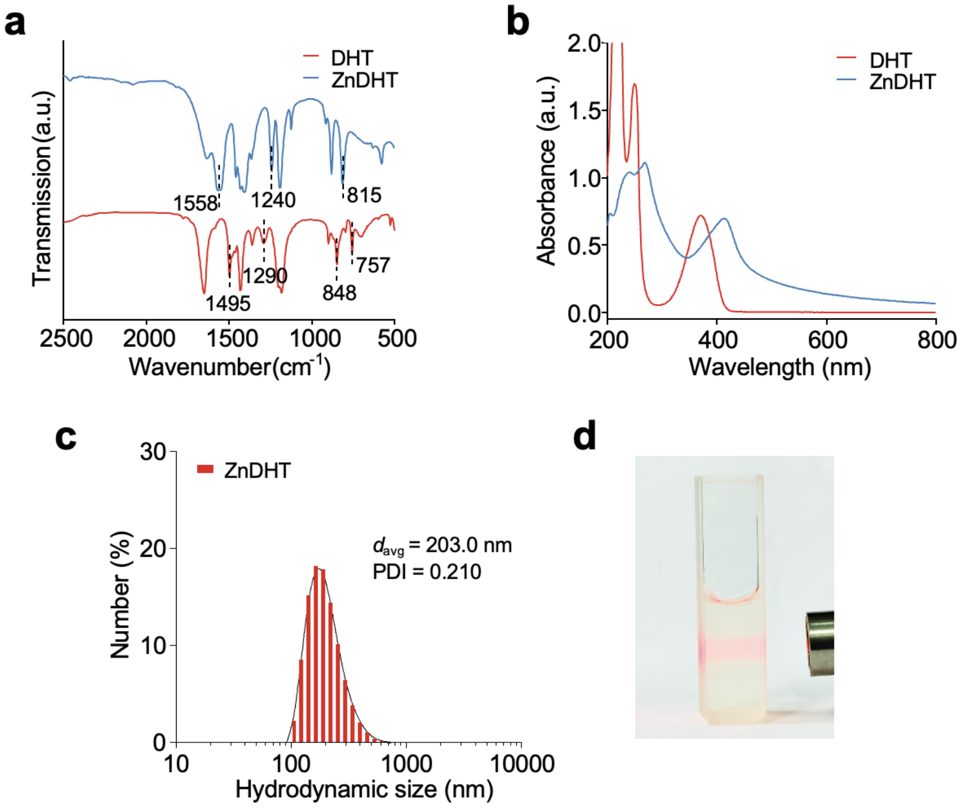

Figure S3. FTIR spectra of DHT and ZnDHT (**a**). The stretching vibration bands of carboxyl groups at 1290 cm^{-1} and 1495 cm^{-1} in DHT were shifted to 1240 cm^{-1} and 1558 cm^{-1} in ZnDHT, respectively, indicating the coordination between Zn^{2+} and carboxylate oxygens of DHT. Moreover, the flexural vibration bands of phenolic hydroxyl groups at 757 cm^{-1} and 848 cm^{-1} in DHT were combined into 815 cm^{-1} in ZnDHT, suggesting the coordination between Zn^{2+} and phenate oxygens of DHT. Accordingly, Zn^{2+} is able to coordinate with both phenate and carboxylate oxygens in DHT ligands to form ZnDHT structure. UV-Vis absorption spectra of DHT and ZnDHT (**b**). ZnDHT has a similar UV-Vis absorption spectrum with DHT but with some redshift, attributing to the enlarged conjugate area of DHT after coordination with Zn^{2+}. Hydrodynamic size distribution of ZnDHT analyzed by dynamic light scattering (**c**), and photograph of ZnDHT suspension in H_2O with laser irradiation (**d**) indicating the Tyndall phenomenon of nanoparticles. Both results (**c**,**d**) demonstrate that ZnDHT has good water dispersibility. PDI, polydispersity index.

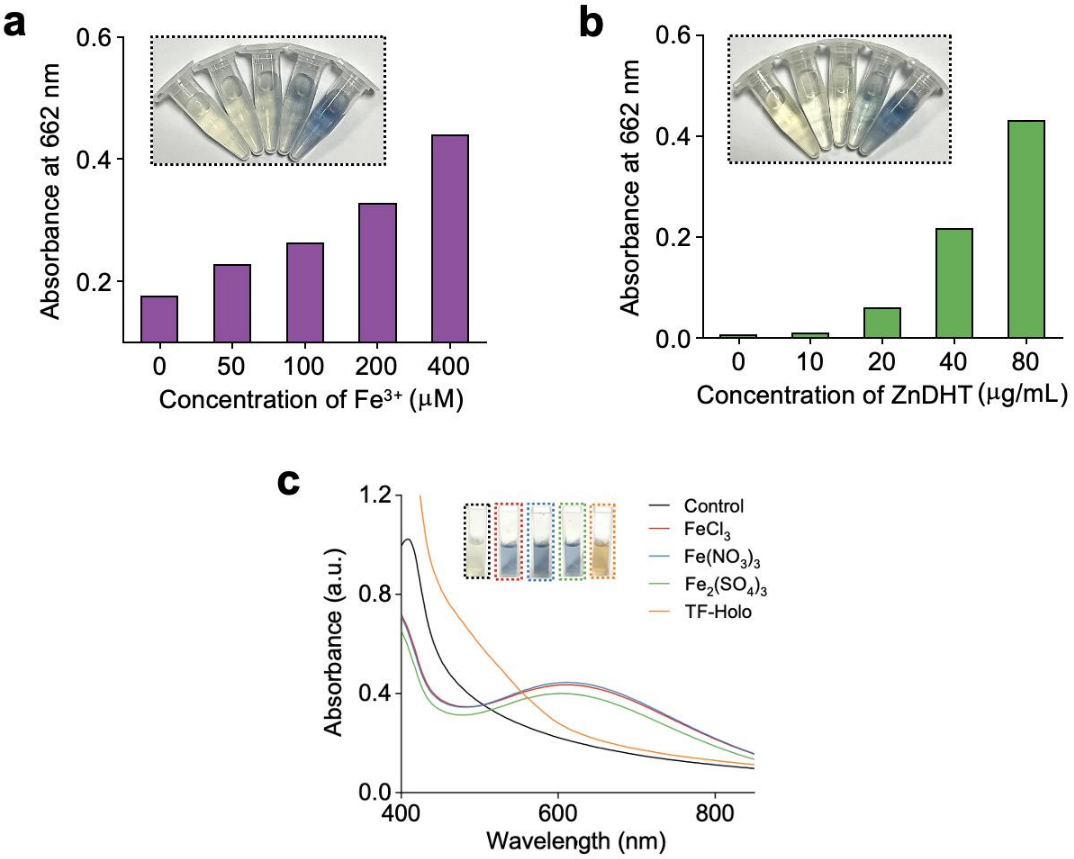

Figure S4. Concentration-dependent UV-Vis absorption (662 nm) of ZnDHT (80 μg/mL) after dispersed in the solution with different concentrations of Fe^{3+} (**a**), or that of different contents of ZnDHT after dispersed in the solution with Fe^{3+} (400 μM) (**b**). UV-Vis absorption spectra of ZnDHT after dispersed in the solutions containing different iron salts or TF (Holo) (**c**). Insert: the photographs of these tested solutions after reactions. No color change and characteristic UV-Vis absorbance peak were observed after mixing ZnDHT with TF-Holo in the solution, indicating that ZnDHT can hardly react with the Fe^{3+} coordinated on TF-Holo directly.

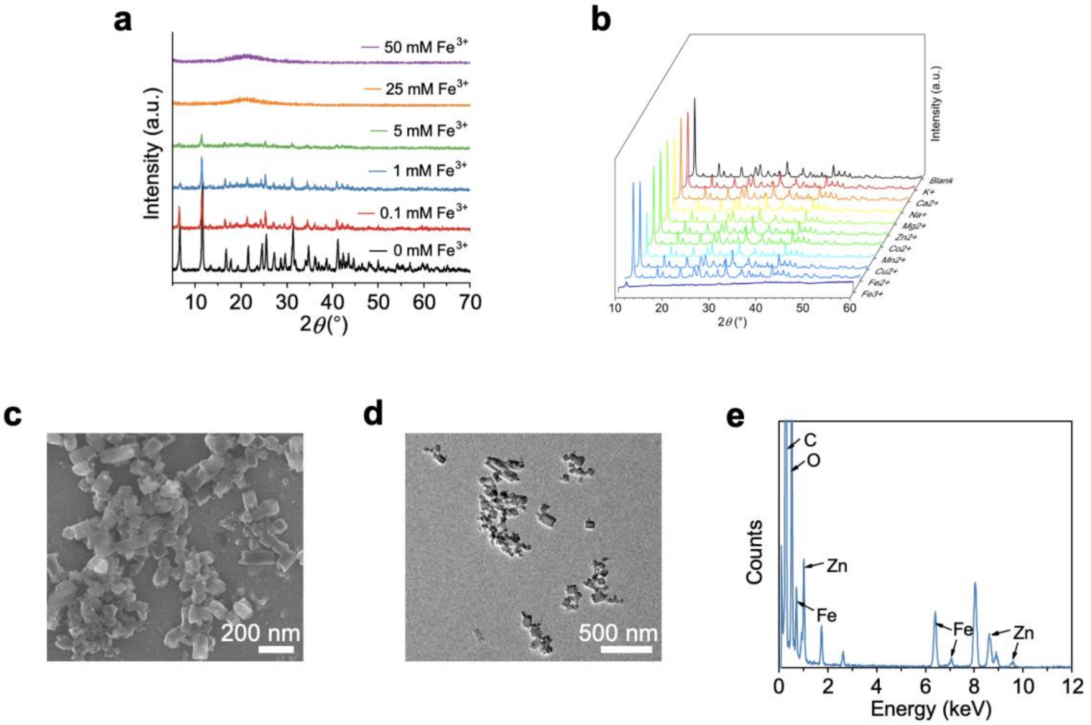

Figure S5. XRD patterns of ZnDHT after reaction with different concentrations of Fe^{3+} (**a**) or with different kinds of metal ions (**b**). The blank group in (**b**) represents the ZnDHT without treatment. SEM image (**c**), TEM image (**d**), and EDS profile (**e**) of ZnFeDHT prepared by reacting ZnDHT (1 mg/mL) with Fe^{3+} (5 mM).

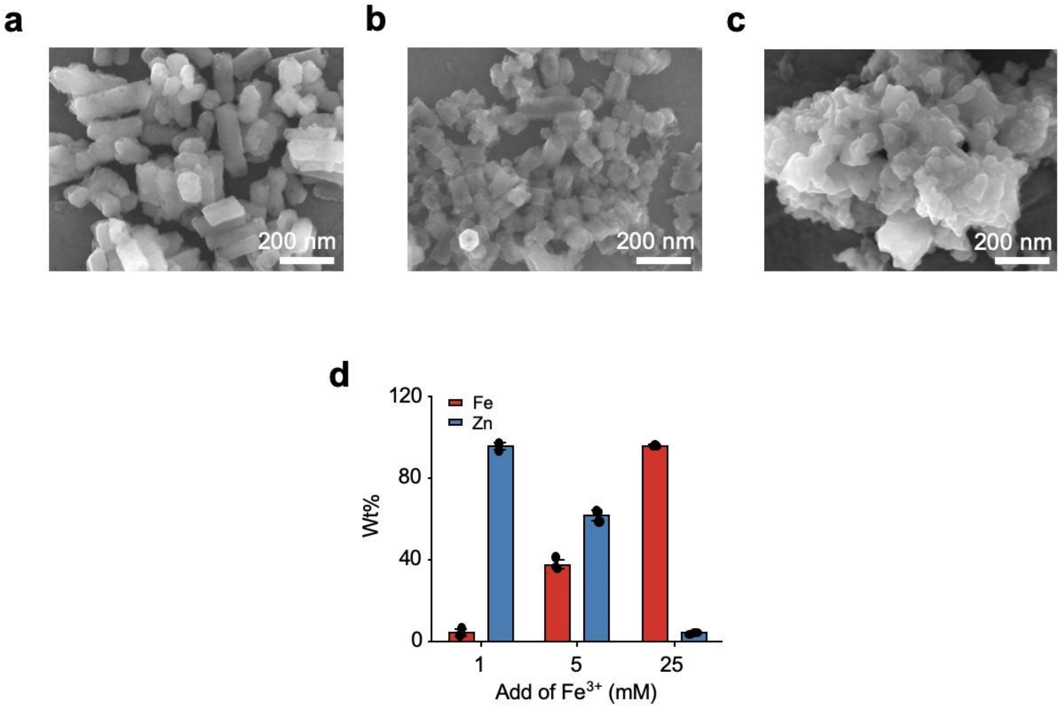

Figure S6. SEM images of ZnDHT after reaction with different concentrations of Fe^{3+} (**a**, 1mM; **b**, 5 mM; **c**, 25 mM), and corresponding Fe and Zn element mass ratios in nanoparticles analyzed by EDS (**d**). As the amount of Fe^{3+} in the reaction increased, the surface of ZnDHT was first eroded, and further the rod-shaped structure gradually destroyed to release Zn^{2+} until the morphology became completely irregular. Data are expressed as mean ± s.e.m. (n = 3 independent experiments).

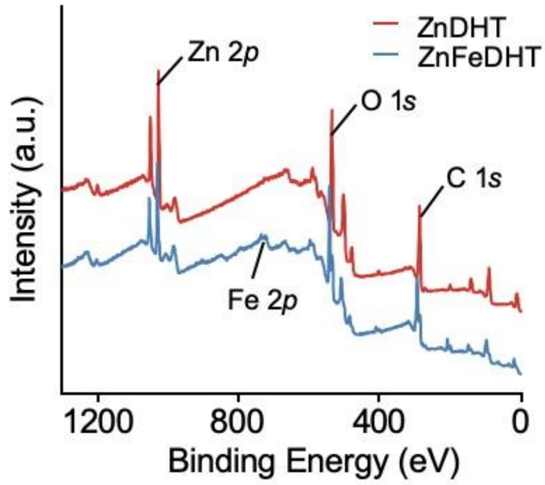

Figure S7. Full survey XPS spectra of ZnDHT and ZnFeDHT.

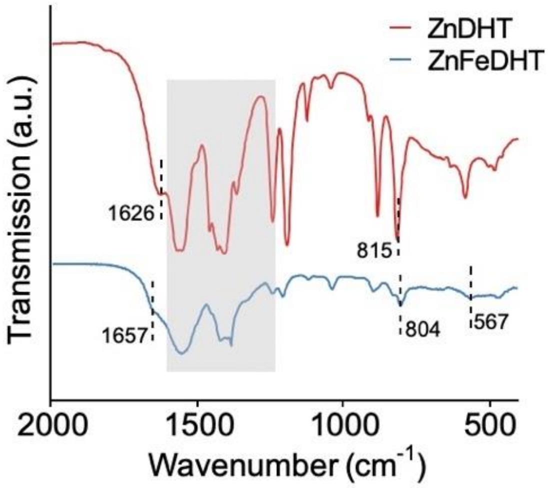

Figure S8. FTIR spectra of ZnDHT and ZnFeDHT. The stretching vibration bands of carboxyl groups at the range from 1600 cm^{-1} to 1200 cm^{-1} in ZnDHT were varied significantly after forming ZnFeDHT, suggesting that Fe^{3+} coordinated with the carboxylate oxygens of DHT in the structure thereby affecting the initial coordination between Zn^{2+} and DHT. The stretching vibration of the C=O bond in carboxyl groups at 1626 cm^{-1} vanished while a new C=O stretching at 1657 cm^{-1} appeared, probably due to the existence of benzoquinone in ZnFeDHT [8]. In addition, the flexural vibration band of phenolic hydroxyl groups at 815 cm^{-1} in ZnDHT were shifted and weakened after forming ZnFeDHT, indicating that Fe^{3+} also coordinated with the phenate oxygens of DHT. The new peak at 567 cm^{-1} could be attributed to the stretching vibration of Fe-O bond.

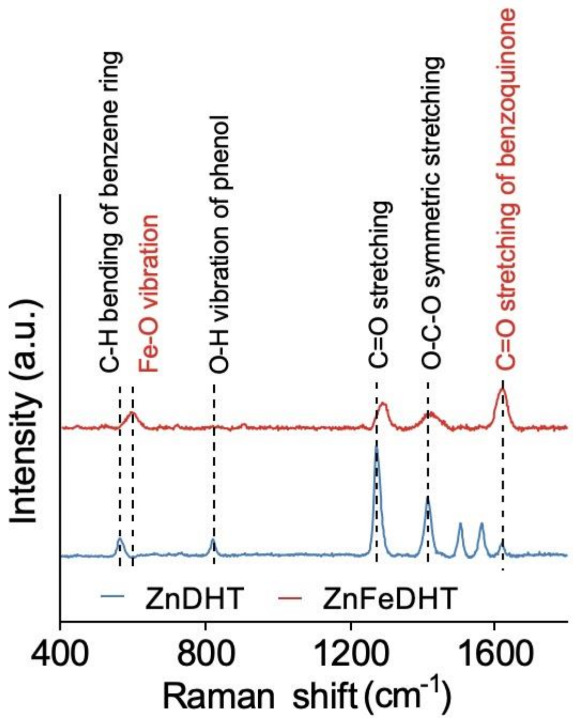

Figure S9. Raman spectra of ZnDHT and ZnFeDHT. The new bands in the low-frequency region (640~560 cm^{-1}) of ZnFeDHT could be assigned to the Fe-O vibration. Compared to ZnDHT, the vibrations of the bonds in both carboxyl and phenolic hydroxyl groups of ZnFeDHT were varied, proving the coordination of Fe^{3+} with both phenate and carboxylate oxygens of DHT in the ZnFeDHT structure. In addition, the most prominent peak located at 1622 cm^{-1} of ZnFeDHT could be associated with the C=O stretching mode of benzoquinone [9].

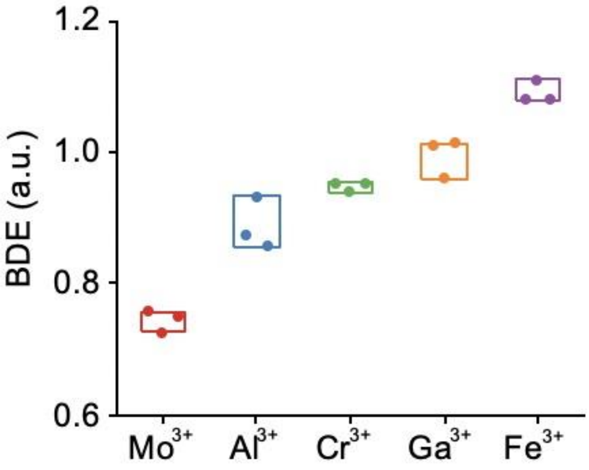

Figure S10. The calculated bond dissociation energies (BDEs) between DHT and different trivalent metal ions in the three coordinated modes (**M**-O carboxylate, **M**-O phenate, and O-**M**-O bridge-type).

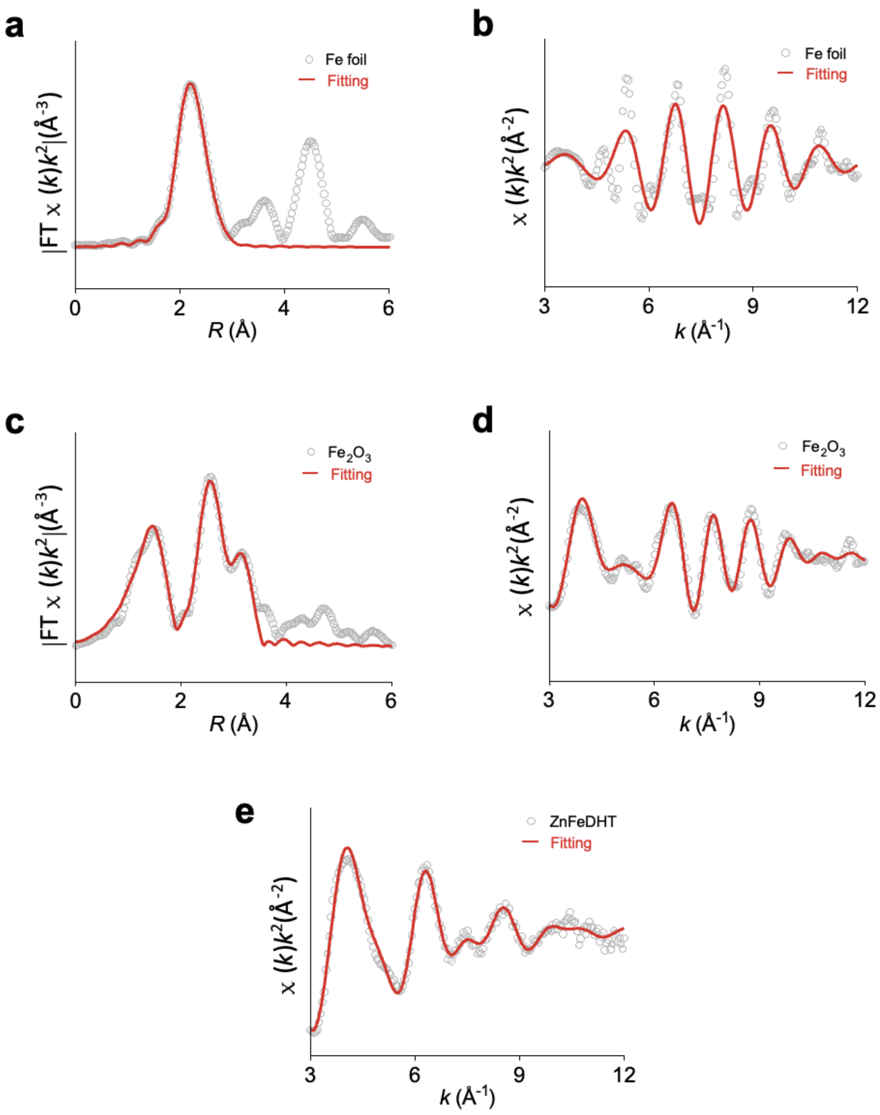

Figure S11. Fe K-edge EXAFS fitting curves of Fe foil and Fe_2O_3 in *R* space (**a**,**c**) and *k* space (**b**,**d**). Fe K-edge EXAFS fitting curve of ZnFeDHT in *k* space (**e**).

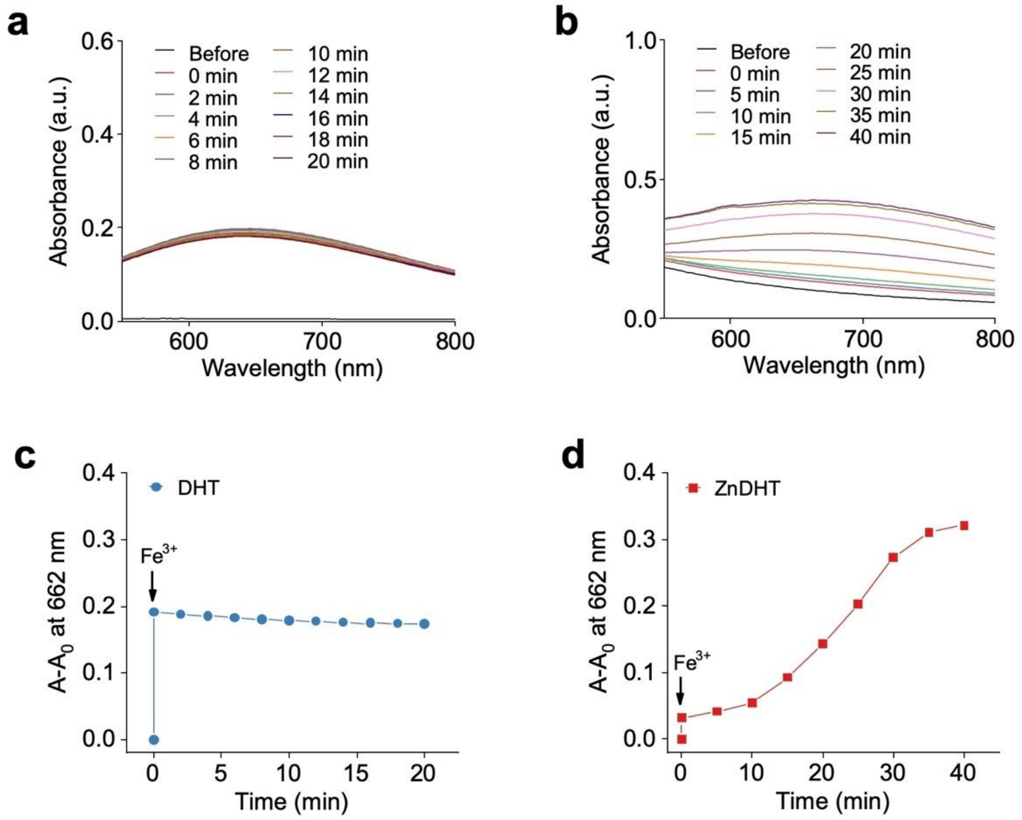

Figure S12. UV-Vis absorption spectra of DHT (**a**) and ZnDHT (**b**) after dispersed in the Fe^{3+}-contained solution for different times, and corresponding time-dependent UV-Vis absorbance (662 nm) changes for DHT group (**c**) and ZnDHT group (**d**).

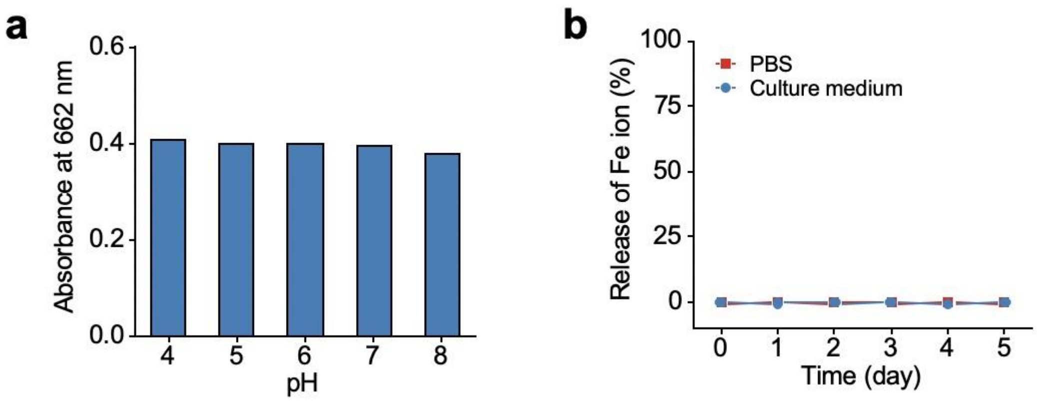

Figure S13. UV-Vis absorption (662 nm) of ZnDHT (80 μg/mL) after dispersed in the solution with Fe^{3+} (400 μM) under the wide pH range (from acid to alkali) (**a**). The characteristic absorptions of the tested solutions with different pH showed little differences, indicating the good pH stability of ZnDHT as a Fe^{3+} capturing agent. Fe ion release curves of ZnFeDHT dispersed in PBS and culture medium (**b**). The negligible Fe ion release from ZnFeDHT was detected in the simulated physiological environments for a long time, suggesting that the Fe^{3+}-chelating effect of ZnDHT is irreversible.

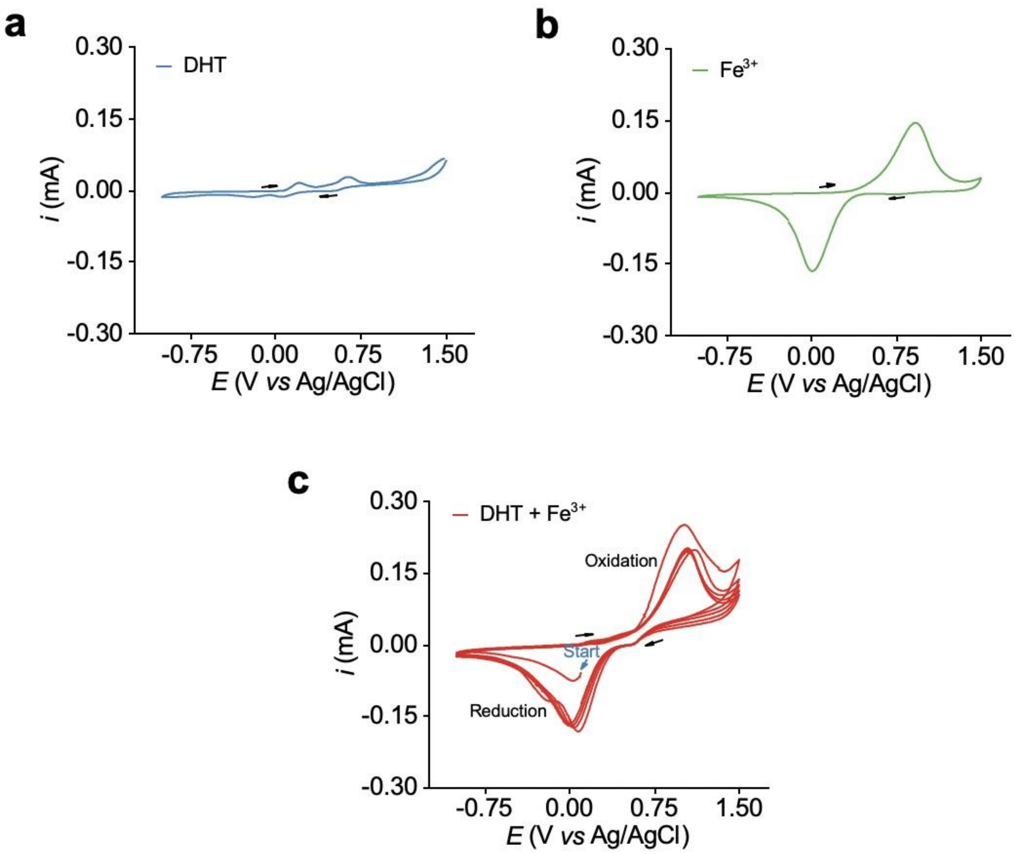

Figure S14. CV curves for the electrolyte solutions containing DHT (**a**) or Fe^{3+} (**b**). Successive CV curves for the electrolyte solution containing both DHT and Fe^{3+} (**c**). These successive CV curves were obtained by originally sweeping from +0.1 to -1.0 V, followed by five cycles. Compared to DHT, an apparent redox cycling could be visualized for Fe^{3+} during CV measurement. By investigating the redox behavior of coordinated Fe-DHT structure in the electrolyte solution based on the successive CV measurement, we found that, in the original reduction process (i.e., +0.1 to -1.0 V), the reduction peak was significantly weakened, confirming that the coordination between Fe^{3+} and DHT ligand endows the Fe sites of Fe-DHT with enhanced reducibility resulting in preventing the acceptance of additional electrons from external environment. In addition, during the subsequent cycles of CV, the oxidation and reduction peaks were visible with the first decrease and then stability in the current, indicative of the redox response character of Fe-DHT structure combining of irreversible DHT oxidation and reversible Fe redox cycling.

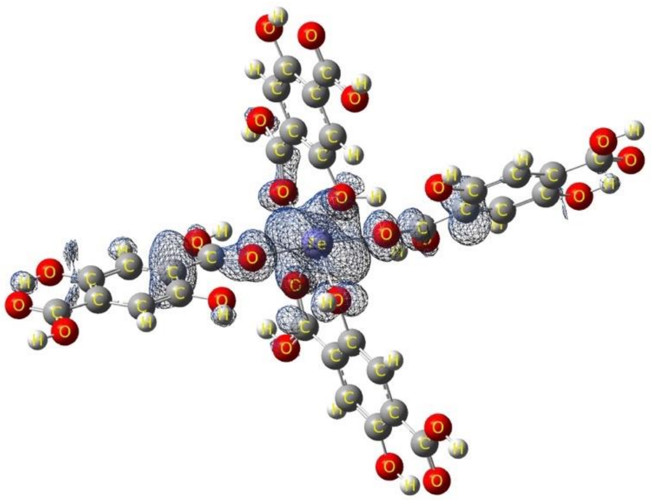

Figure S15. Calculated spin density of the optimized hexacoordinated Fe-DHT conformation based on the DFT calculations.

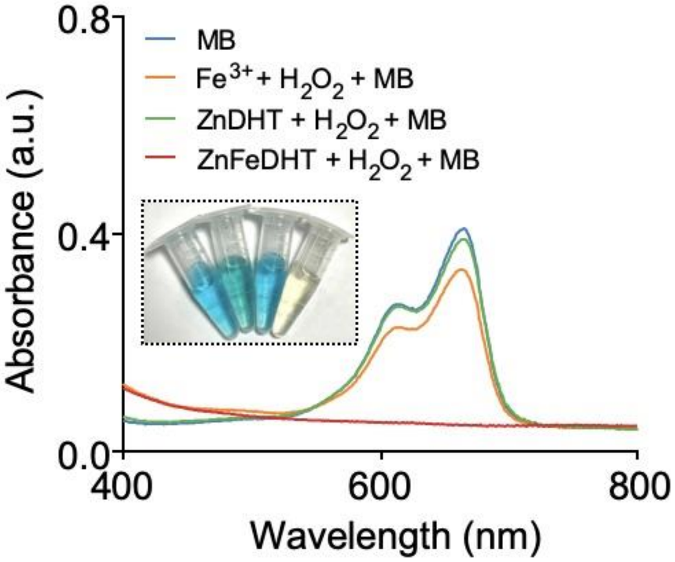

Figure S16. UV-Vis absorption spectra of the H_2O_2-contained buffer solutions (pH 5.2) with MB as an indicator in the presence of ZnDHT, Fe^{3+}, or fresh ZnFeDHT. Insert: the photographs of these tested solutions after reactions.

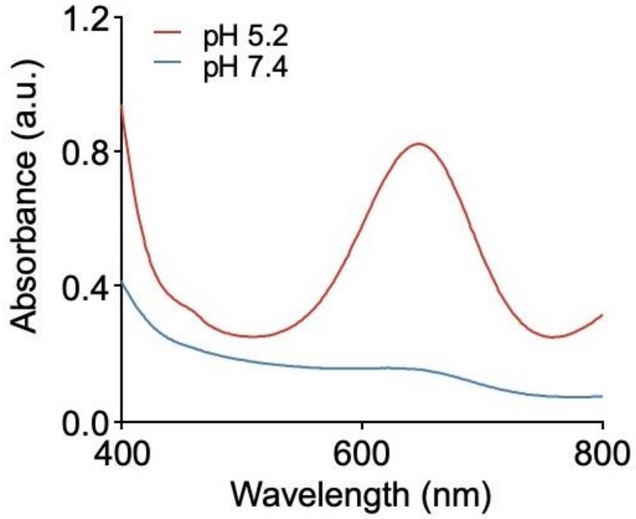

Figure S17. UV-Vis absorption spectra of the H_2O_2-contained buffer solutions under different pH (5.2 and 7.4) with TMB as a colorimetric indicator in the presence of fresh ZnFeDHT.

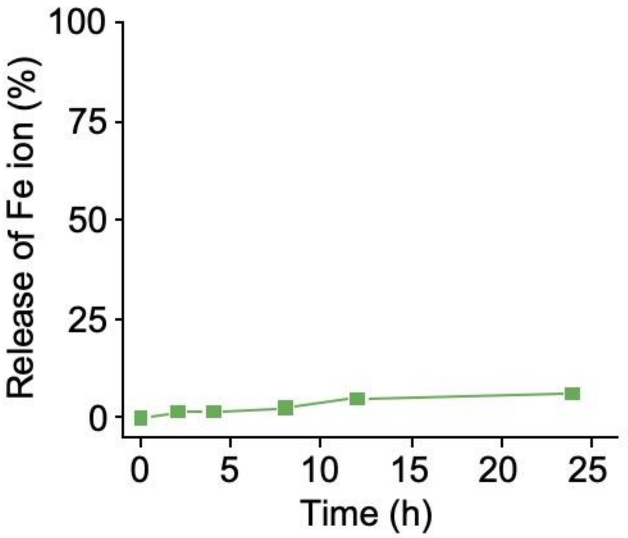

Figure S18. Fe ion release curve of ZnFeDHT in the PBS (pH 6.0; H_2O_2, 100 μM) that simulates the tumor microenvironment. The inapparent Fe ion release from ZnFeDHT during the catalytic process was detected in the simulated environment, proving the structural integrity of the ZnFeDHT after redox reactions.

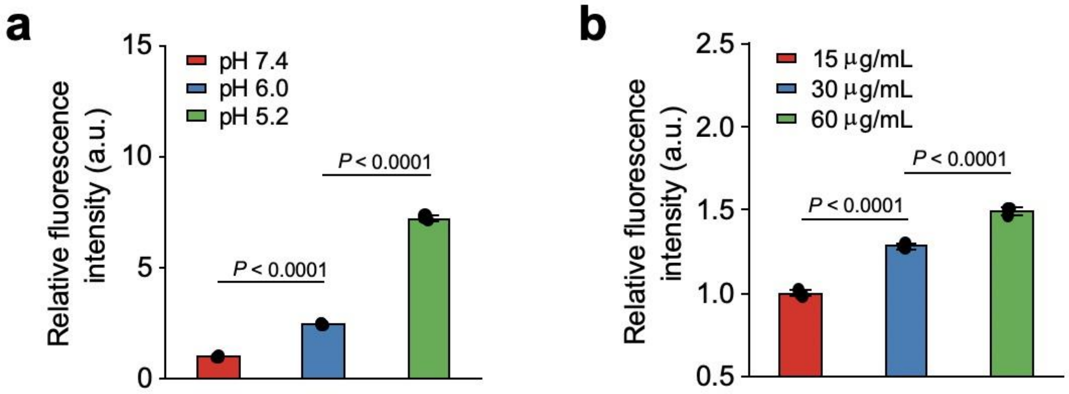

Figure S19. Relative fluorescence intensities of the buffer solutions under different pH with *o*-phenylenediamine (OPD) as a fluorescent indicator and with fresh ZnFeDHT (**a**). Relative fluorescence intensities of the buffer solutions (pH 5.2) with OPD as a fluorescent indicator and with different concentrations of fresh ZnFeDHT (**b**). Thereinto, different concentrations of ZnFeDHT were prepared by mixing different contents of ZnDHT with the same content of Fe^{3+} in the test solution. Data are expressed as mean ± s.e.m. (n = 3 independent experiments). Statistical significance was determined by one-way ANOVA with Tukey's post-hoc test.

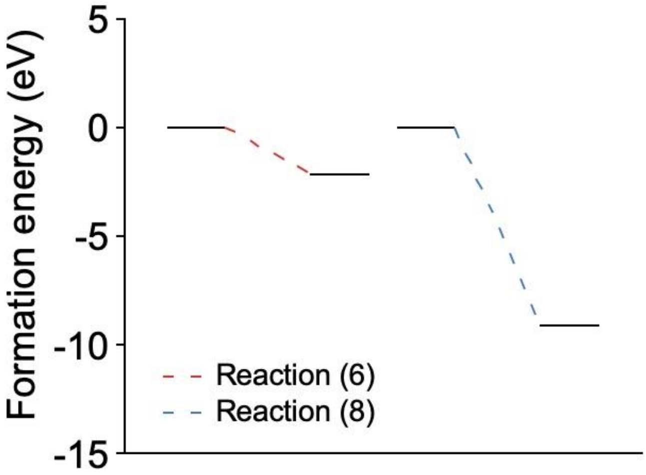

Figure S20. Comparison of the formation energies of $[Fe^{II}\text{-sqDHT}\bullet]^-$ + O_2 and $[Fe^{III}\text{-sqDHT}\bullet]$ + $O_2\bullet^-$, representing the reaction (6); Comparison of the formation energies of $[Fe^{II}\text{-qDHT}]$ + $O_2\bullet^-$ + $2H^+$ and $[Fe^{III}\text{-qDHT}]^+$ + H_2O_2, representing the reaction (8).

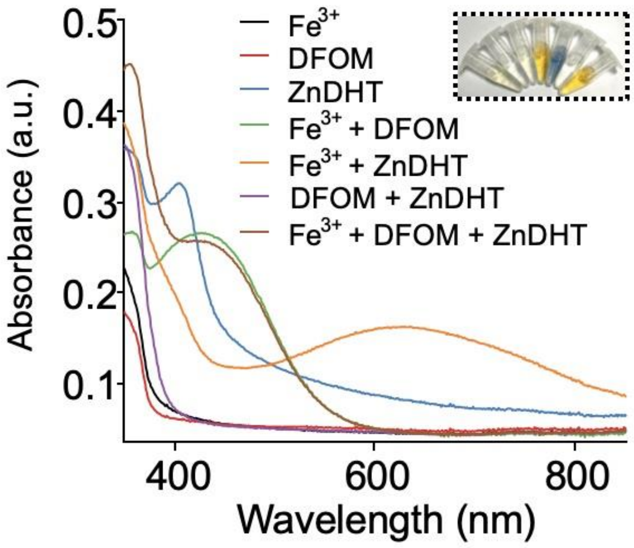

Figure S21. UV-Vis absorption spectra of DFOM and/or ZnDHT after dispersed in the solutions with or without Fe^{3+}. Insert: the photographs of these tested solutions after reactions. The colorless DFOM can easily chelate Fe^{3+} in the solution to form the yellow Fe-DFOM complex showing a characteristic absorption at 431 nm. It can be observed that the simultaneous reaction of Fe^{3+} with both of DFOM and ZnDHT resulted in producing a yellow solution with the characteristic absorption at 431 nm but no absorption at 662 nm, suggesting that DFOM exhibits a stronger binding force toward Fe^{3+} to form coordination complex than ZnDHT.

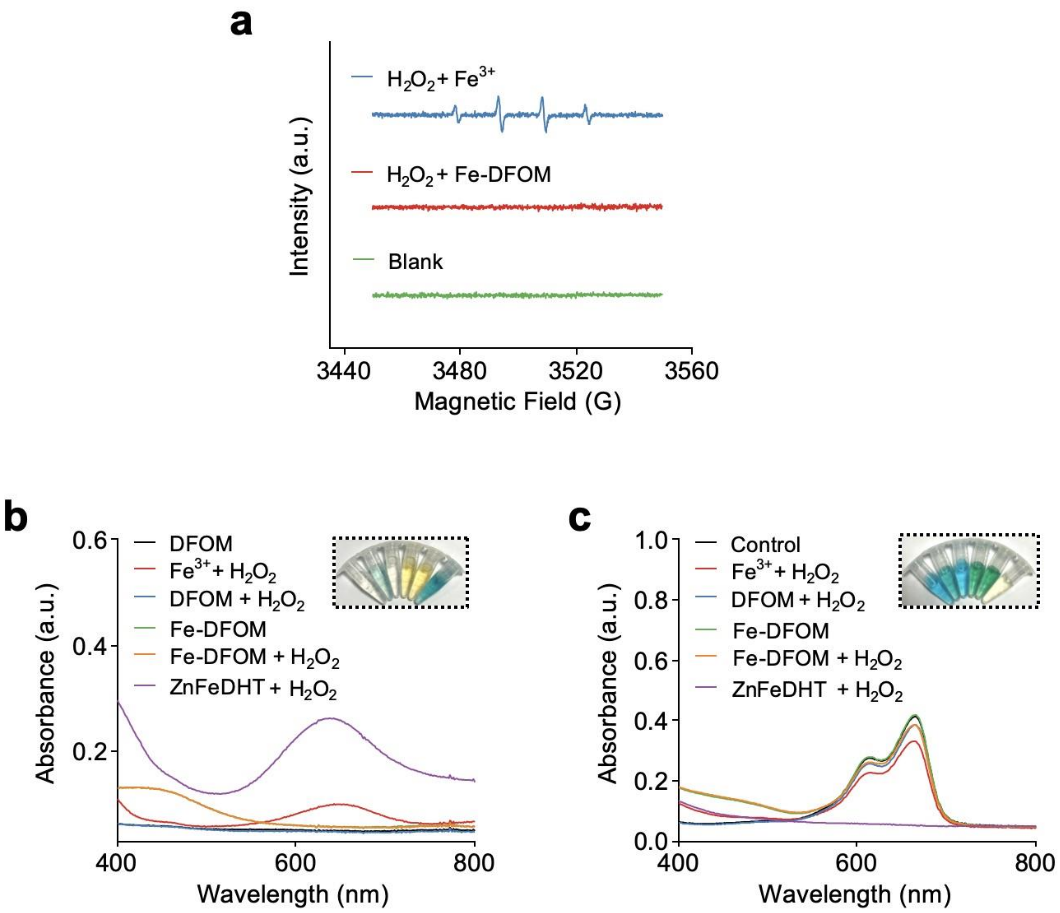

Figure S22. ESR spectra evaluating •OH generation in the H_2O_2-contained buffer solution (pH 5.2) with Fe^{3+} or fresh Fe-DFOM complex (**a**). UV-Vis absorption spectra of the H_2O_2-contained buffer solutions (pH 5.2) with TMB (**b**) or MB (**c**) as the indicator in the presence of Fe^{3+}, DFOM, fresh Fe-DFOM complex, or fresh ZnFeDHT. Insert: the photographs of these tested solutions after reactions. Prior to each trial, fresh Fe-DFOM complex was prepared through mixing DFOM (400 μM) with Fe^{3+} (400 μM) directly in the test solution, and the same concentration of free Fe^{3+} was set for comparison. According to the ESR results, compared to the free Fe^{3+} group, there was no significant •OH signal observed in the Fe-DFOM group, because of the redox-inactive nature of Fe-DFOM complex that blocks the catalytic •OH generation from H_2O_2 by Fenton reactions [10]. Both TMB and MB colorimetric assays demonstrate that compared to free Fe^{3+}, Fe-DFOM complex restricts the catalytic conversion of H_2O_2 to •OH while ZnFeDHT obviously boosts the •OH generation.

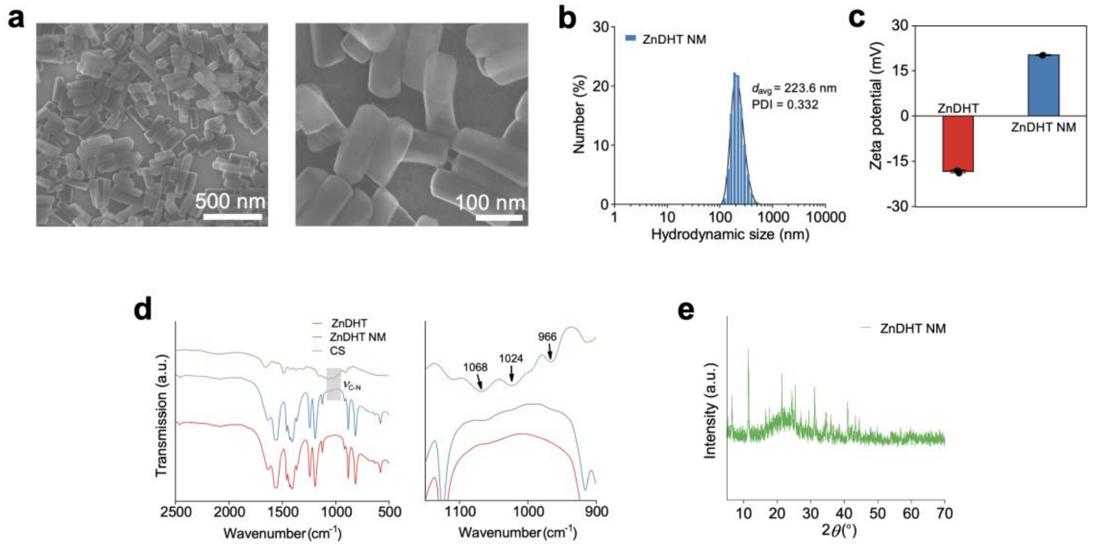

Figure S23. SEM images (**a**), hydrodynamic size distribution (**b**), and zeta potential (**c**) of ZnDHT NM. The average hydrodynamic size of ZnDHT NM was larger than that of ZnDHT (203.0 nm, **Figure S3c**), and after the CS modification, the zeta potential of nanoparticles changed from -18.4 mV to +20.3 mV. Both results testify the successful modification of CS on the ZnDHT. FTIR spectra of CS, ZnDHT, and ZnDHT NM (**d**). Compared to ZnDHT, the additive bands in the range of 1100 cm^{-1} to 950 cm^{-1} in ZnDHT NM could be assigned to the stretching vibration of the C-N bond of CS, owing to the modification of CS on the nanoparticles. XRD pattern of ZnDHT NM (**e**). The XRD characteristic peaks of ZnDHT after the reaction did not change significantly, indicating that the CS modification negligibly destroyed the crystalline structure of ZnDHT (**Figure S2d**). Data are expressed as mean ± s.e.m. (*n* = 3 independent experiments).

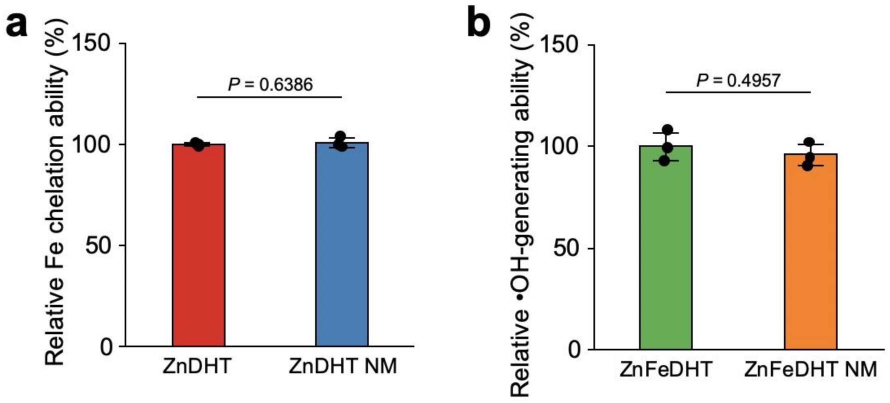

Figure S24. Comparison of Fe chelation ability between ZnDHT and ZnDHT NM by dispersing them in the solutions with Fe^{3+} (**a**). Comparison of •OH-generating ability between ZnFeDHT and ZnFeDHT NM in the H_2O_2-contained buffer solutions (pH 5.2) with TMB as a colorimetric indicator (**b**). Prior to each trial, fresh ZnFeDHT NM was prepared in the test solution using the same way as preparing ZnFeDHT. ZnDHT NM exhibited the similar Fe chelation ability to ZnDHT, and there was insignificant difference in catalytic •OH generation performance between ZnFeDHT and ZnFeDHT NM, suggesting that the CS modification did not impair the functions of ZnDHT. Data are expressed as mean ± s.e.m. (n = 3 independent experiments). Statistical significance was determined by two-tailed Student's t-test.

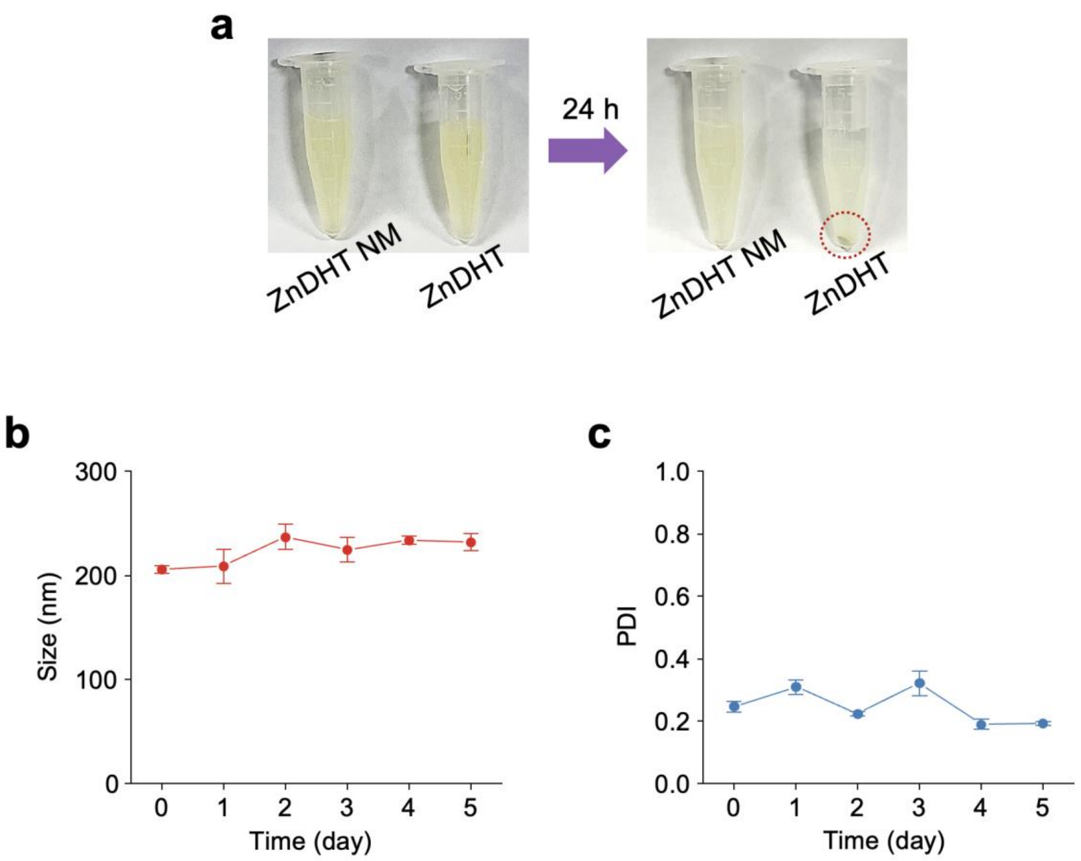

Figure S25. Photographs of ZnDHT NM and ZnDHT dispersed in water before and after 24 h (**a**). As shown in figures, ZnDHT NM was well dispersed in water to form homogeneous dispersions, and still remained stable after 24 h of incubation. However, an obvious agglomeration phenomenon was observed for ZnDHT after 24 h of incubation. This result confirms that ZnDHT NM exhibits the enhanced colloidal stability compared to ZnDHT, owing to the decoration of hydrophilic CS on the surface. Accordingly, ZnDHT NM with good stability will be suitable for biomedical applications. Hydrodynamic size (**b**) and PDI (**c**) changes of ZnDHT NM after incubation with BSA (10 mg/mL). There were no significant variations in both particle size and PDI of ZnDHT NM during the incubation, demonstrating the high stability to serum proteins that benefits *in vivo* applications. Data are expressed as mean ± s.e.m. (*n* = 3 independent experiments).

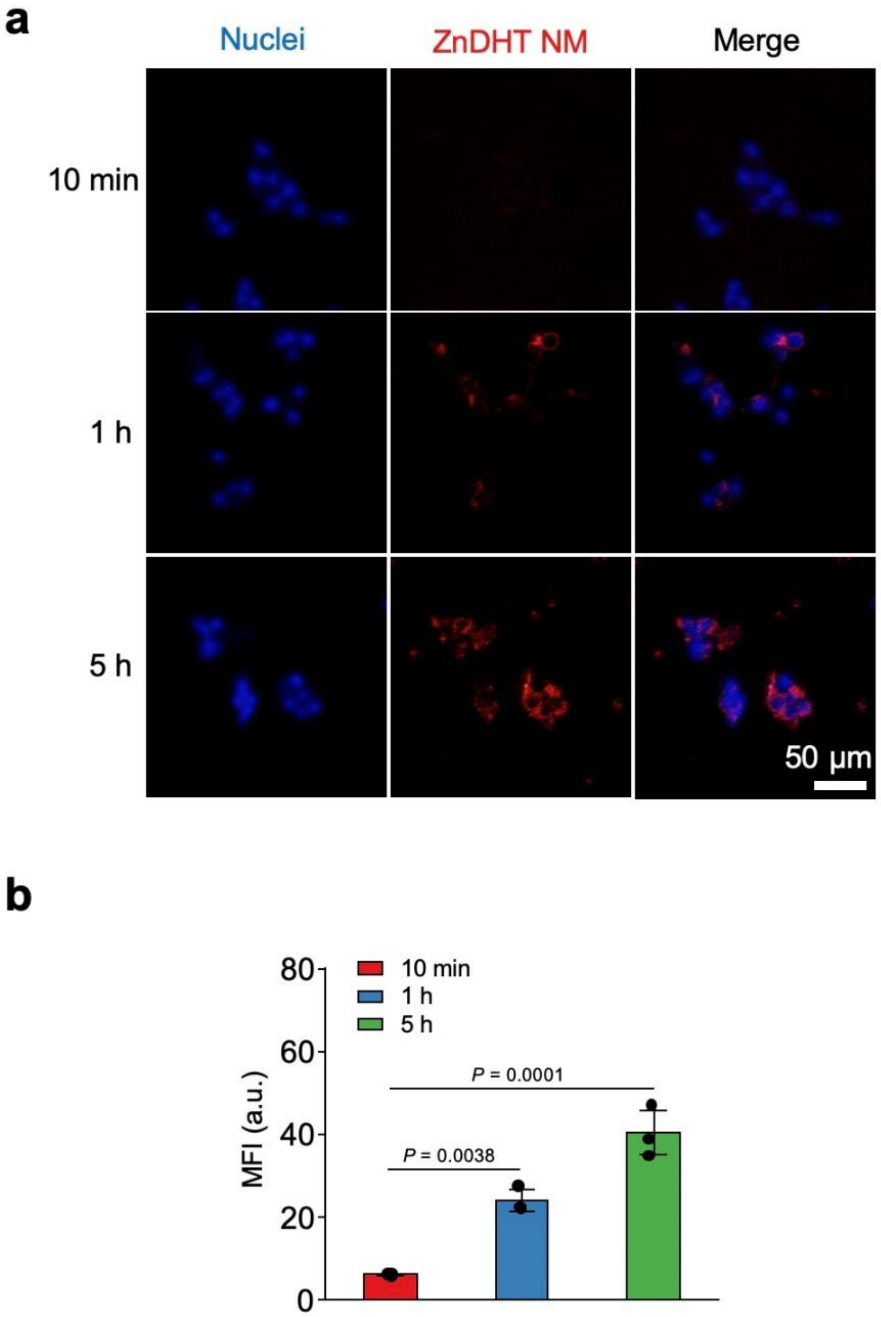

Figure S26. Time-dependent cell uptake of ZnDHT NM by 4T1 cells after incubation for 10 min, 1 h, and 5 h (blue fluorescence represents the cell nuclei; red fluorescence represents the Cy5-labeled ZnDHT NM) (**a**). Corresponding red fluorescence intensities of different groups in **a** (**b**). Data are expressed as mean ± s.e.m. (*n* = 3 independent experiments). Statistical significance was determined by one-way ANOVA with Tukey's post-hoc test.

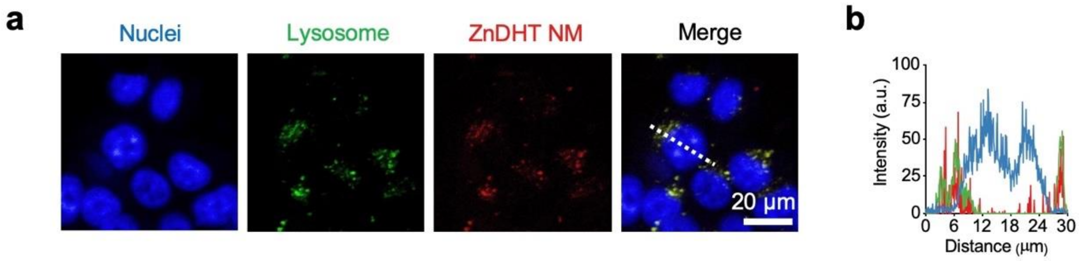

Figure S27. Fluorescence images of 4T1 cells after incubation of ZnDHT NM for 1 h (blue fluorescence represents the cell nuclei; green fluorescence represents the lysosomes; the red fluorescence represents the Cy5-labeled ZnDHT NM) (**a**). Corresponding fluorescence intensities (including blue, green, and red) of the area marked by a white dashed line in **a** (**b**). The fluorescence images exhibit effective co-location of the nanomedicines and the lysosomes, indicating that ZnDHT NM can be endocytosed by tumor cells and then enter the endosomes and lysosomes of cells.

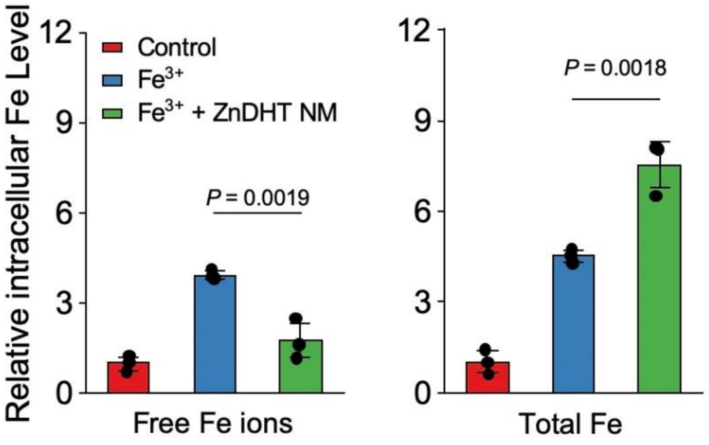

Figure S28. Analysis of the intracellular free Fe ion (left) or the intracellular total Fe content (right) of 4T1 cells after treatment of Fe^{3+} or Fe^{3+} plus ZnDHT NM. The simultaneous treatment of free Fe^{3+} and ZnDHT NM increased the total Fe level within cells compared to that of only Fe^{3+}, suggesting that ZnDHT NM could chelate the Fe^{3+} in the medium and then be internalized by tumor cells. Despite the elevated total Fe level within cells, the addition of ZnDHT NM significantly lowered the free Fe ion level within Fe^{3+}-incubated tumor cells. It can be explained as the Fe^{3+}-chelated ZnDHT NM (i.e., ZnFeDHT NM) could not release Fe ions into cells after being internalized, but be able to continuously chelate the intracellular Fe ions by its remaining oxygen chelating sites. From this perspective, treatment of ZnDHT NM would benefit the depletion of both tumor extracellular Fe ions and intracellular Fe ions as well as increase the presence of therapeutic ZnFeDHT NM within tumor cells to induce cell death. Data are expressed as mean ± s.e.m. (n = 3 independent experiments). Statistical significance was determined by one-way ANOVA with Tukey's post-hoc test.

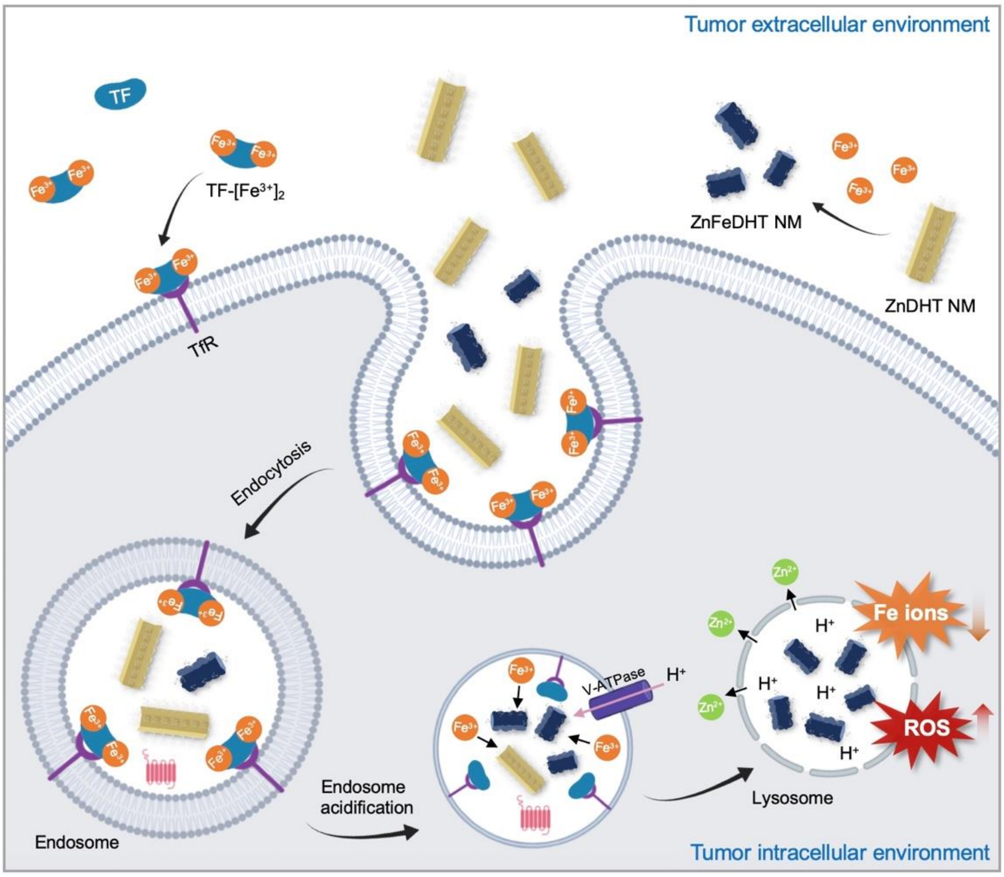

Figure S29. Schematic illustration of the endogenous Fe^{3+} capturing mechanism of ZnDHT NM in the tumor microenvironment. Typically, the transferrin (TF) pathway is used by cells for Fe uptake [11]. One TF can carry two Fe^{3+} to form the complex TF-$[Fe^{3+}]_2$ circulating through the bloodstream, which binds to its receptor, transferrin receptor (TfR), at the cell surface and is endocytosed by cells. Subsequently, these delivered Fe^{3+} are released in the acidic endosomes of cells [12]. According to the cellular Fe metabolism and our experimental results, herein we propose a possible mechanism for the ZnDHT NM-mediated endogenous Fe^{3+} capturing in the tumor microenvironment. On the one hand, after entering the tumor microenvironment, a part of ZnDHT NM could firstly chelate the extracellular free Fe^{3+} to form ZnFeDHT NM; on the other hand, these endocytosed nanomedicines (ZnDHT NM/ZnFeDHT NM) by tumor cells could further chelate the TF-$[Fe^{3+}]_2$-released Fe^{3+} in the acidic endosomes and be sent onwards to the acidic lysosomes [13], resulting in the effective Fe depletion and ROS generation as well as the Zn^{2+} release within the cells.

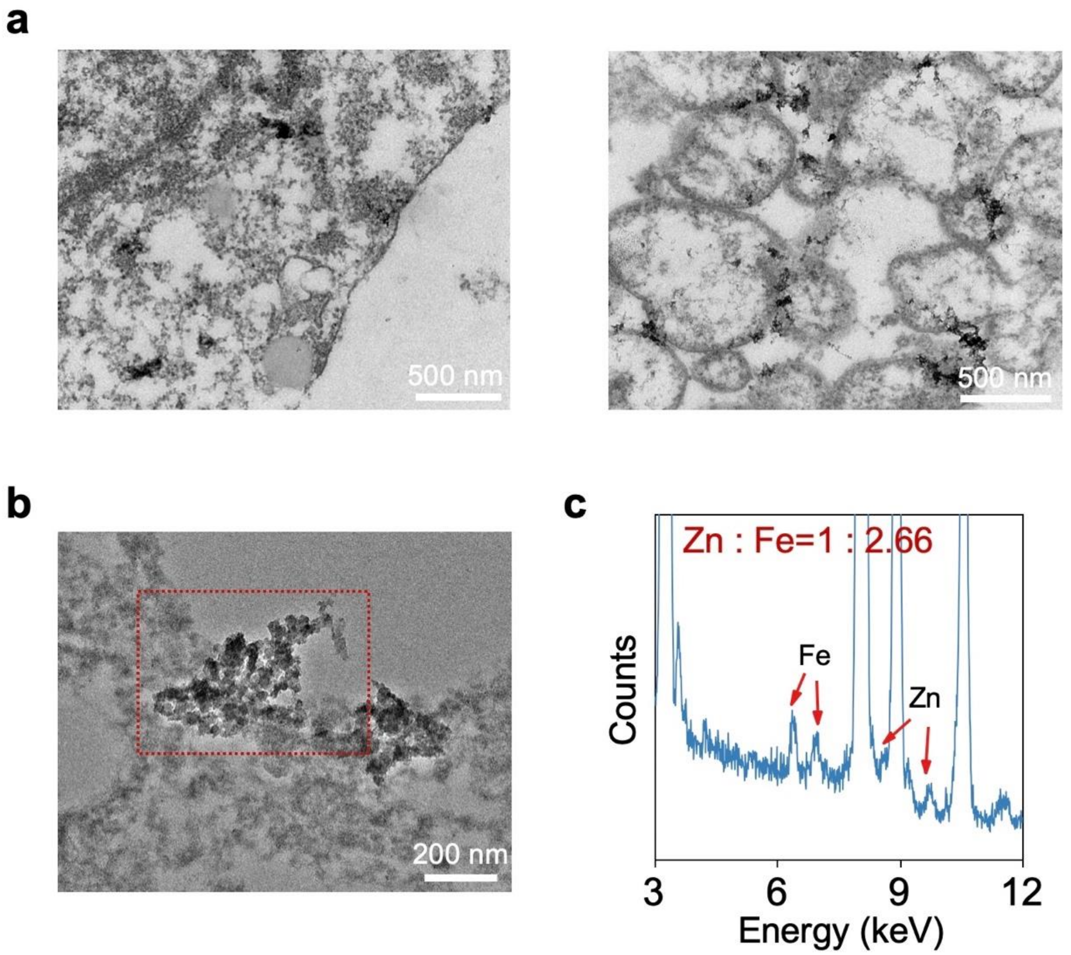

Figure S30. TEM images of 4T1 cells after incubation of ZnDHT NM (**a**). Locally magnified TEM image of 4T1 cells after incubation of ZnDHT NM (**b**) and corresponding EDS profile (**c**). Red dashed box indicates the ZnFeDHT NM formed within cells and the position for EDS analysis. The Zn/Fe element mass ratio in the formed ZnFeDHT NM was calculated as 1 : 2.66.

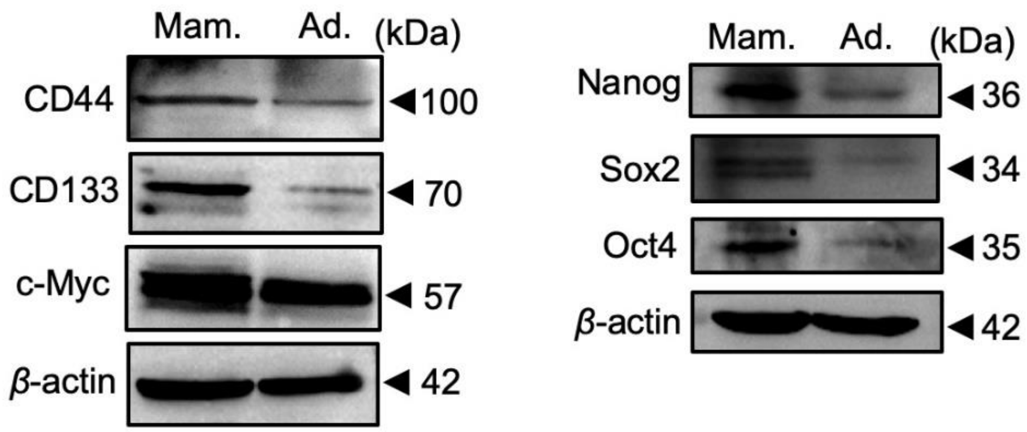

Figure S31. Western blot analysis of the expressions of the CSC surface markers and stemness-related proteins in 4T1 mammosphere cells (Mam.) and adherent cells (Ad.).

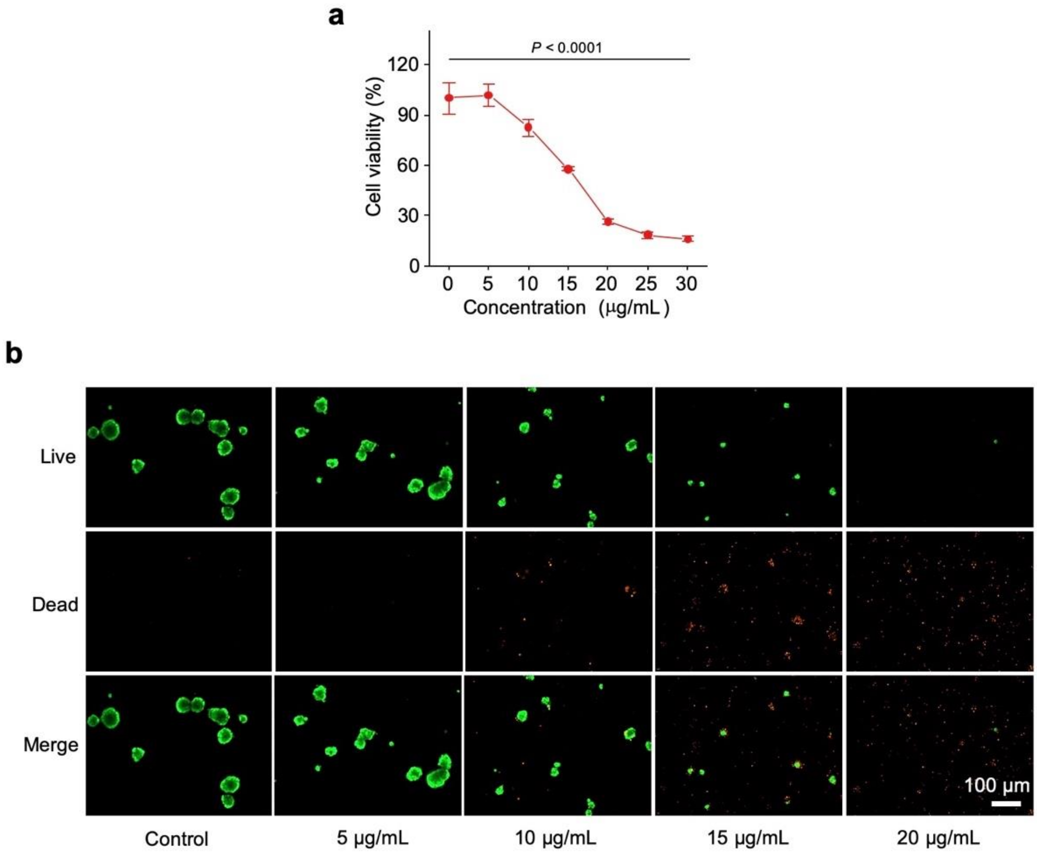

Figure S32. Cell viabilities (**a**) and live/dead cell staining analysis (**b**) of 4T1 mammosphere cells cultivated in the serum-free culture medium with treatment of different concentrations of ZnDHT NM for 48 h. The green fluorescence represents the live cells, and the red fluorescence represents the dead cells. Data are expressed as mean ± s.e.m. (n = 4 independent experiments). Statistical significance was determined by one-way ANOVA with Tukey's post-hoc test.

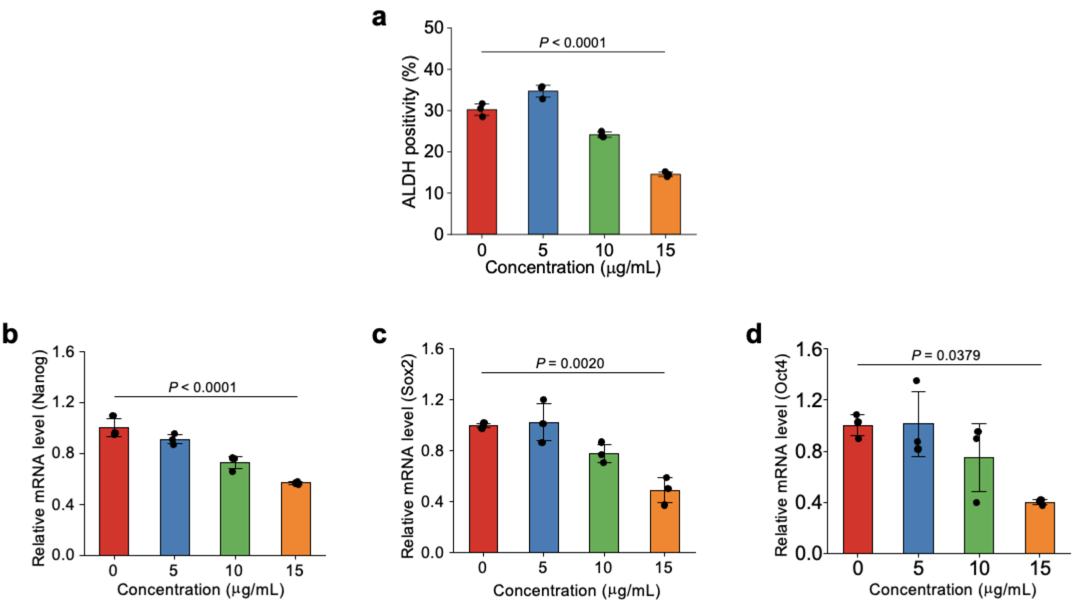

Figure S33. Statistic results of the percentage of ALDH^{high} cells sorted from 4T1 mammosphere cells upon treatment with different concentrations of ZnDHT NM, obtained by flow cytometric analysis (**a**). RT-qPCR analysis of the relative gene expressions of Nanog (**b**), Sox2 (**c**), and Oct4 (**d**) in 4T1 mammosphere cells upon treatment with different concentrations of ZnDHT NM. Data are expressed as mean ± s.e.m. (n = 3 independent experiments). Statistical significance was determined by one-way ANOVA with Tukey's post-hoc test.

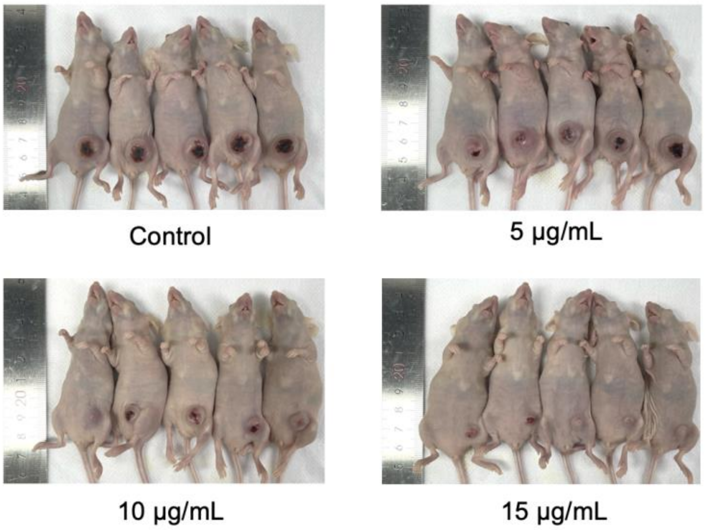

Figure S34. Photographs of the mice on day 21 post inoculation of the 4T1 mammosphere cells upon treatment with different concentrations of ZnDHT NM.

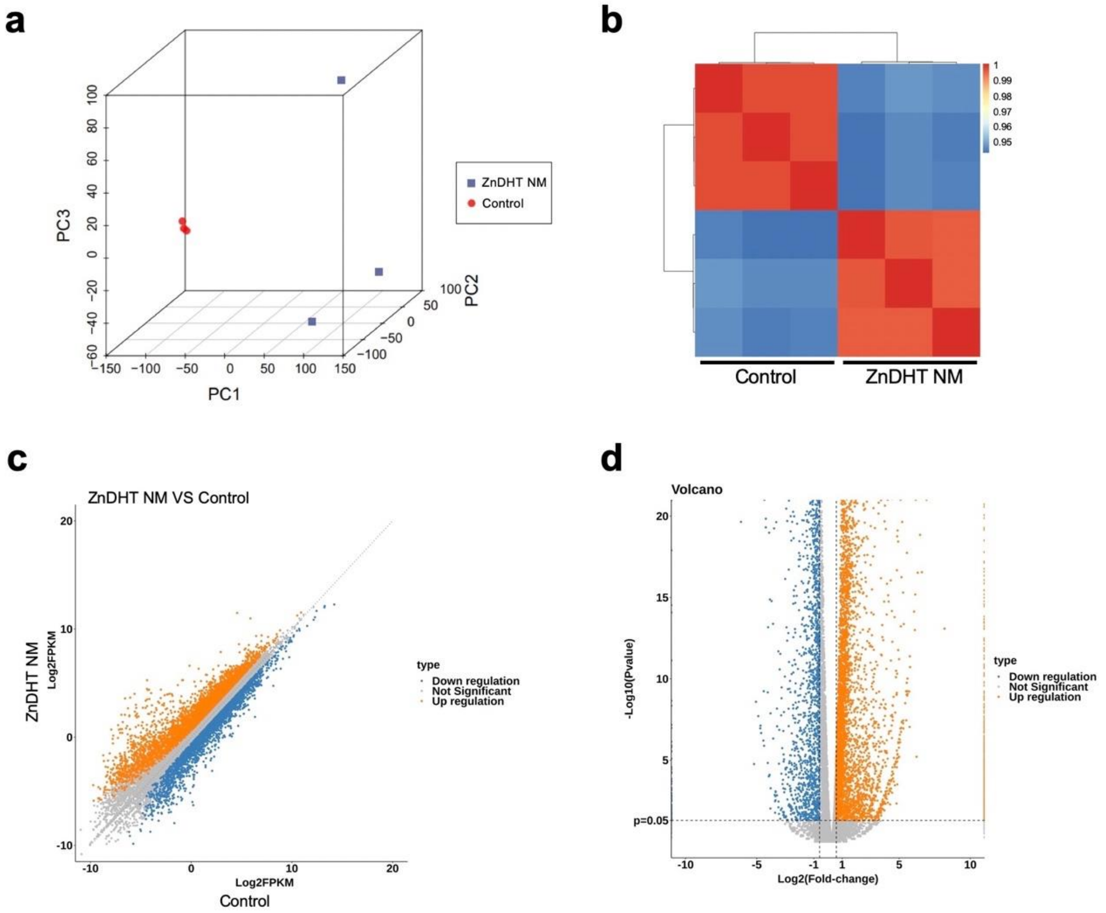

Figure S35. Principal component analysis (**a**) and correlation heatmap analysis (**b**) between different groups (control and ZnDHT NM) in the whole RNA-seq (*n* = 3). Scatter plot (**c**) and volcano plot (**d**) showing the identified upregulated and downregulated genes by ZnDHT NM.

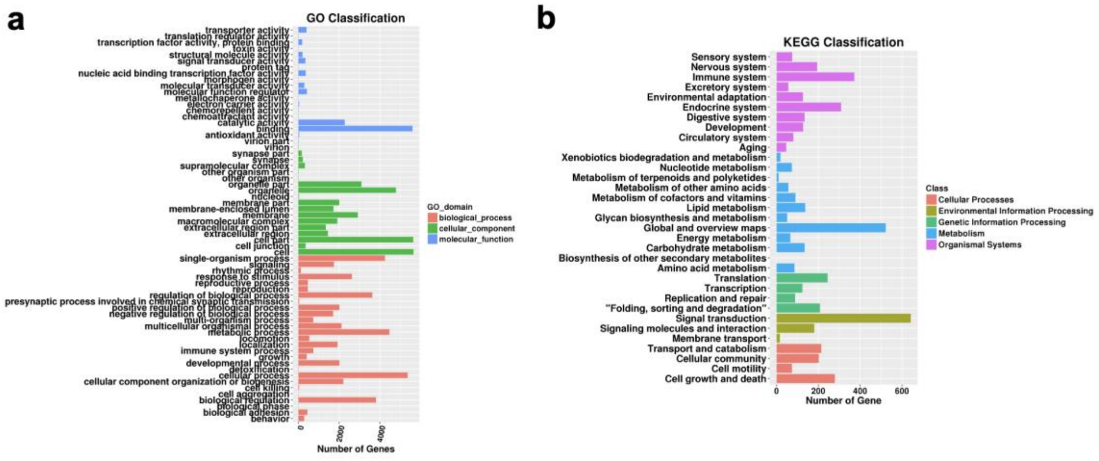

Figure S36. GO classification analysis for showing the biological process, cellular component, and molecular function associated genes (**a**). KEGG classification analysis for exhibiting the cellular processes, environmental information processing, genetic information processing, metabolism, and organismal systems associated genes (**b**).

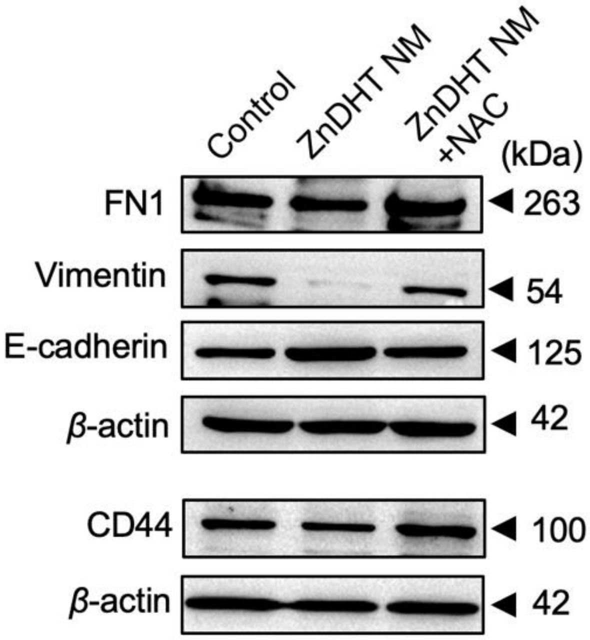

Figure S37. Western blot analysis of the expressions of the EMT (Fibronectin-1 (FN1), Vimentin, and E-cadherin) and CSC (CD44) markers in the ZnDHT NM-treated 4T1 cells with or without the addition of *N*-acetyl-L-cysteine (NAC). The treatment of ZnDHT NM caused in the downregulation of Fibronectin-1 and Vimentin (mesenchymal markers) and the upregulation of E-cadherin (epithelial maker), demonstrating the ability of ZnDHT NM in inhibiting the EMT of 4T1 cells. Moreover, the treatment of ZnDHT NM led to a decrease of CD44 expression, hinting the decline of the CSC populations. However, the further addition of NAC significantly blocked the inhibitory effects of ZnDHT NM on the EMT and CSC properties, uncovering that ROS could be a vital factor in the regulatory mechanisms of ZnDHT NM.

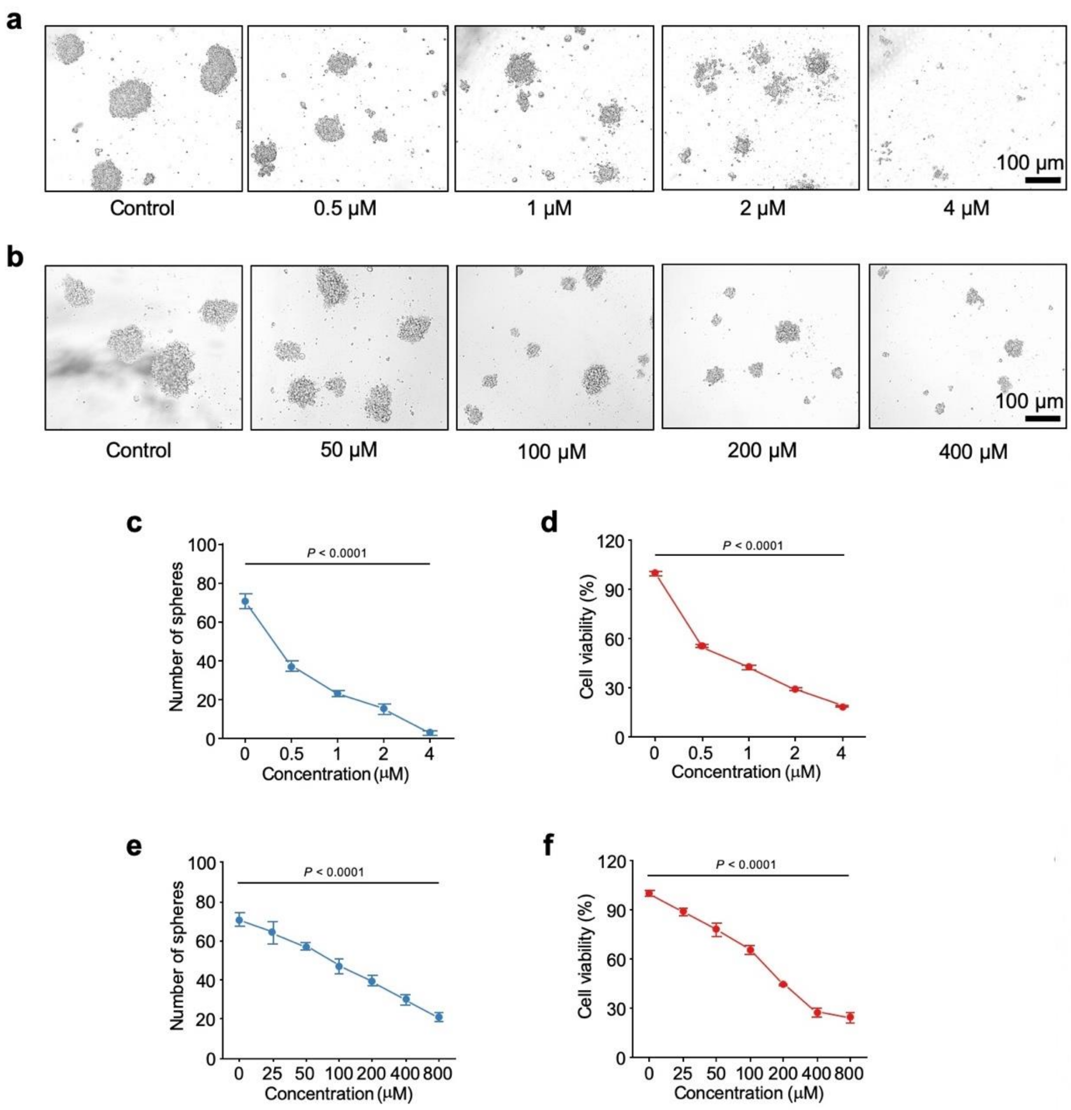

Figure S38. Tumorsphere formation of 4T1 mammosphere cells cultivated in the serum-free culture medium with treatment of different concentrations of DOX (**a**) or DFOM (**b**) for 4 days, and corresponding counted numbers of tumorspheres (diameter > 50 μm) for the DOX (**c**) or DFOM (**e**) group. Cell viabilities of 4T1 mammosphere cells cultivated in the serum-free culture medium with treatment of different concentrations of DOX (**d**) or DFOM (**f**) for 48 h. Data are expressed as mean ± s.e.m. (*n* = 4 independent experiments). Statistical significance was determined by one-way ANOVA with Tukey's post-hoc test.

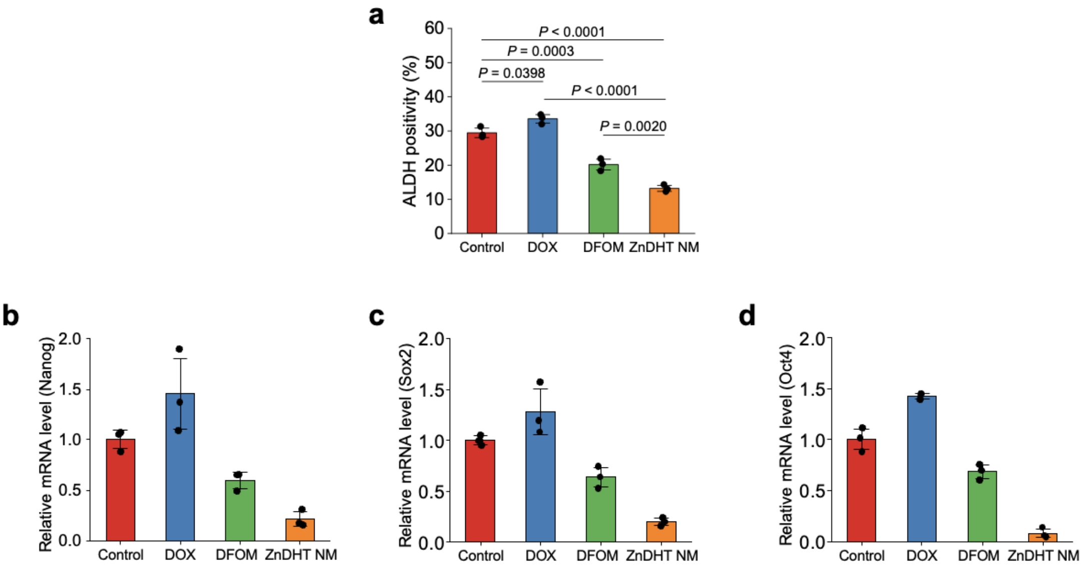

Figure S39. Statistic results of the percentage of ALDH^{high} cells sorted from 4T1 mammosphere cells upon different treatments, obtained by flow cytometric analysis (**a**). RT-qPCR analysis of the relative gene expressions of Nanog (**b**), Sox2 (**c**), and Oct4 (**d**) in 4T1 mammosphere cells upon treatment with different formulations. Data are expressed as mean ± s.e.m. (*n* = 3 independent experiments). Statistical significance was determined by one-way ANOVA with Tukey's post-hoc test.

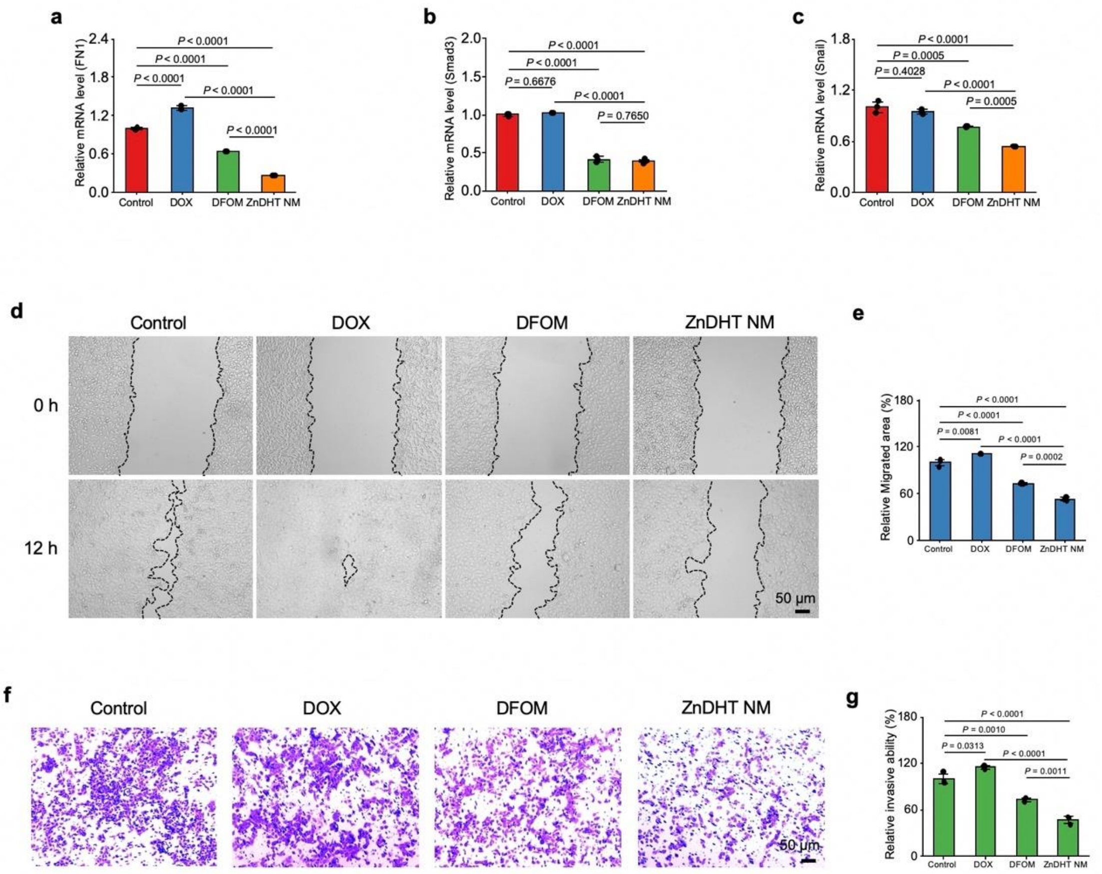

Figure S40. RT-qPCR analysis of the EMT-associated gene expressions in 4T1 cells upon treatment with different formulations (**a**,**b**,**c**). Cell migration of 4T1 cells treated with different formulations (**d**) and corresponding relative migrated area analysis (**e**). Cell invasion of 4T1 cells after treatment of different formulations (**f**) and corresponding relative invasive ability evaluation (**g**). Fibronectin-1 (FN1) is a mesenchymal marker, Smad3 is a regulator in promoting the EMT, and Snail is a mesenchymal-associated transcription factor [14]. As shown in **a**,**b**,**c**, DFOM could inhibit the EMT-associated gene expressions of 4T1 cells to some extent and ZnDHT NM showed the stronger suppressive effects on the EMT. According to the cell migration and invasion assays (**d**-**g**), DOX slightly promoted the migration and invasion of 4T1 cells, while both DFOM and ZnDHT NM prevented their migrated and invasive capacities, where ZnDHT NM possessed the most effective inhibitory effects. Data are expressed as mean ± s.e.m. (*n* = 3 independent experiments). Statistical significance was determined by one-way ANOVA with Tukey’s post-hoc test.

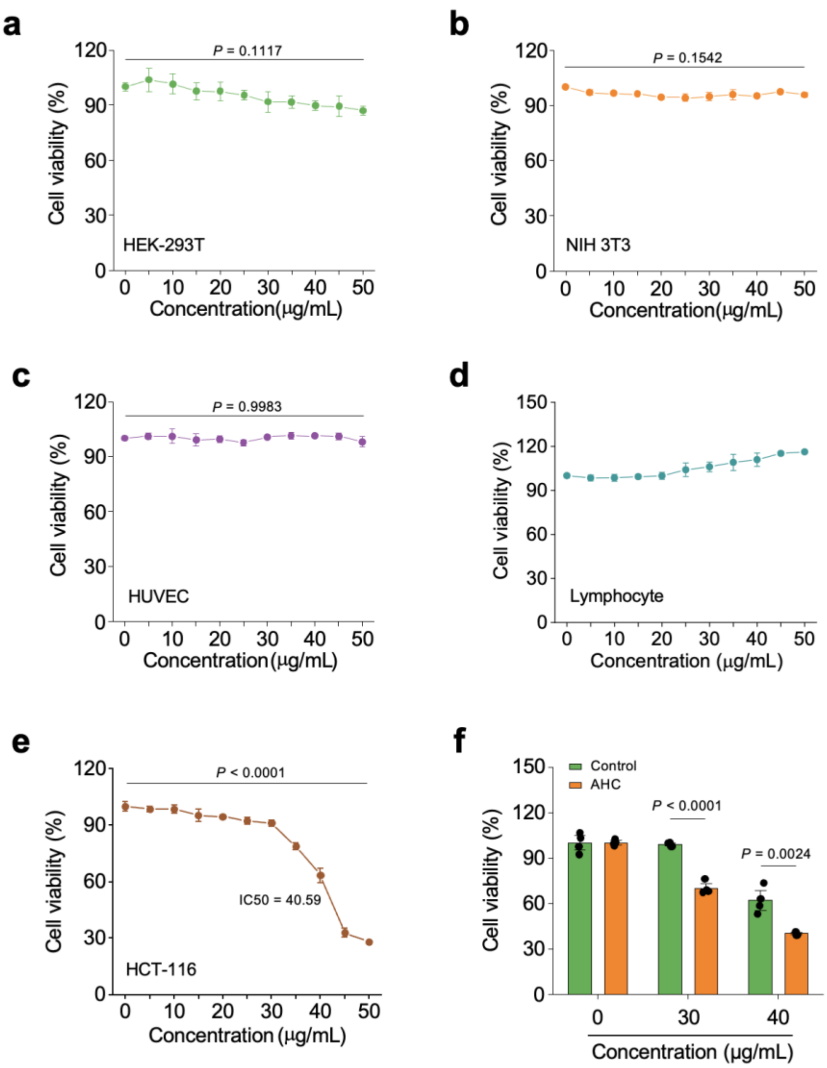

Figure S41. Cell viabilities of HEK-293T (**a**), NIH 3T3 (**b**), HUVEC (**c**), lymphocyte (**d**), HCT-116 (**e**) cells after incubation with different concentrations of ZnDHT NM for 24 h. IC50 represents the half maximal inhibitory concentration. Cell viabilities of HCT-116 cells after incubation with different concentrations of ZnDHT NM under the neutral culture medium (pH 7.4; H_2O_2, 0 μM) (as control) or the mildly acidic culture medium (pH 6.0; H_2O_2, 100 μM) (as the simulated tumor microenvironment, named AHC) for 24 h (**f**). Data are expressed as mean ± s.e.m. (*n* = 4 independent experiments). Statistical significance was determined by one-way ANOVA with Tukey's post-hoc test (**a**-**e**) or two-tailed Student's *t*-test (**f**).

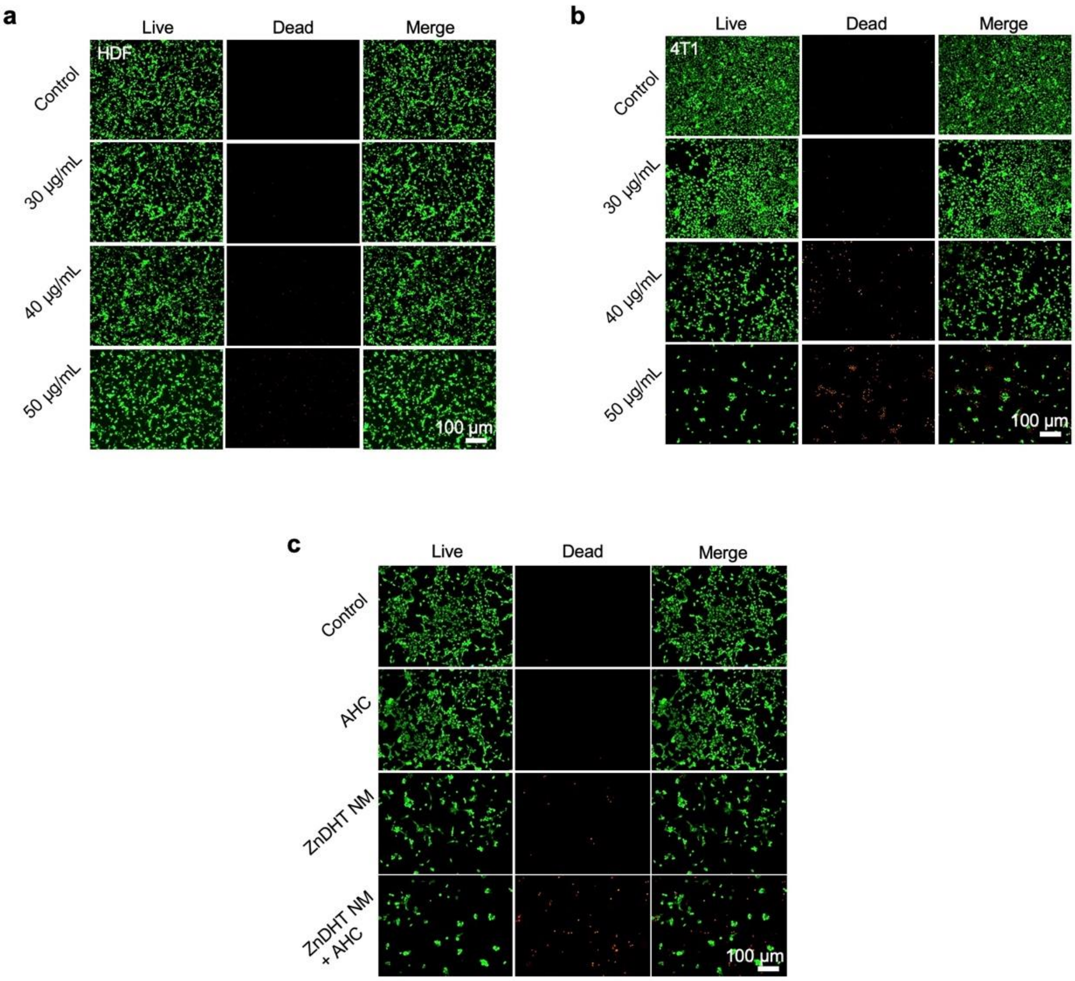

Figure S42. Live/dead cell staining analysis of HDF cells (**a**) and 4T1 cells (**b**) after incubation with different concentrations of ZnDHT NM for 24 h. Live/dead cell staining analysis of 4T1 tumor cells after incubation with or without ZnDHT NM (40 μg/mL) under the neutral culture medium (pH 7.4; H_2O_2, 0 μM) (as control) or the mildly acidic culture medium (pH 6.0; H_2O_2, 100 μM) (as the simulated tumor microenvironment, named AHC) for 24 h (**c**). The green fluorescence represents the live cells, and the red fluorescence represents the dead cells. Similar to the CCK-8 analysis results, ZnDHT NM caused a dose-dependent killing effect on tumor cells but no obvious killing effect on normal cells, demonstrating the tumor cell-selective toxicity of ZnDHT NM and its high biocompatibility toward normal cells. In addition, the AHC further enhanced the cytotoxic effect of ZnDHT NM on tumor cells, proving the tumor microenvironment-specific therapeutic ability of ZnDHT NM.

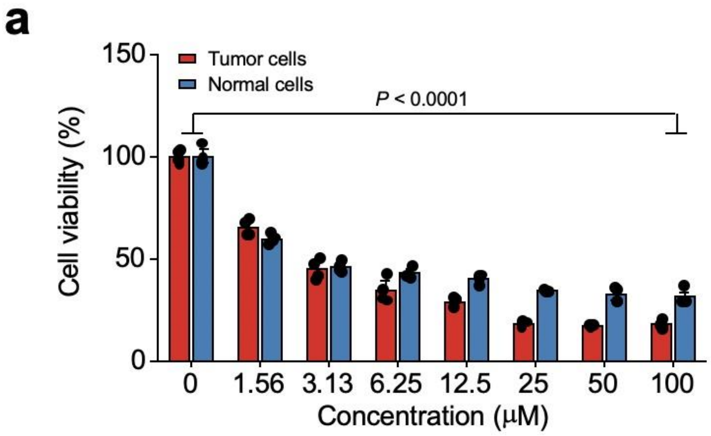

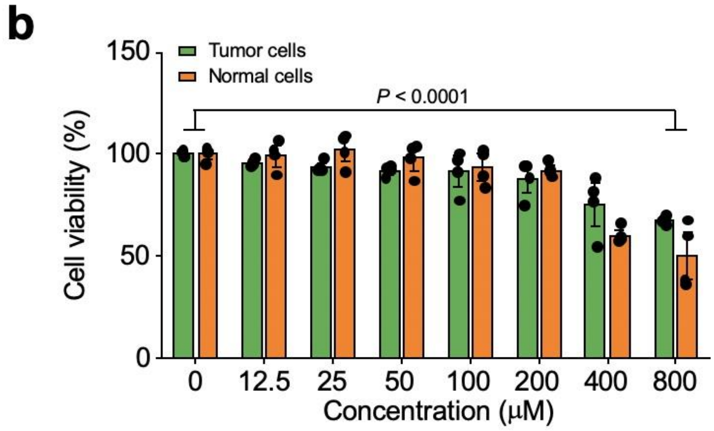

Figure S43. Cell viabilities of 4T1 tumor cells and HEK-293T normal cells after incubation with different concentrations of DOX (**a**) or DFOM (**b**) for 24 h. DOX exhibited high cytotoxicity on both tumor cells and normal cells, and only 3.13 μM of DOX killed over 50% of these cells. In contrast, DFOM showed moderate toxic effects on both tumor cells and normal cells, and the viabilities of tumor cells and normal cells still exceeded 50% after incubation with DFOM at the concentration as high as 800 μM. These results evidence that both of DOX and DFOM do not have highly significant tumor cell-selective therapeutic effectiveness. Data are expressed as mean ± s.e.m. (*n* = 4 independent experiments). Statistical significance was determined by one-way ANOVA with Tukey's post-hoc test.

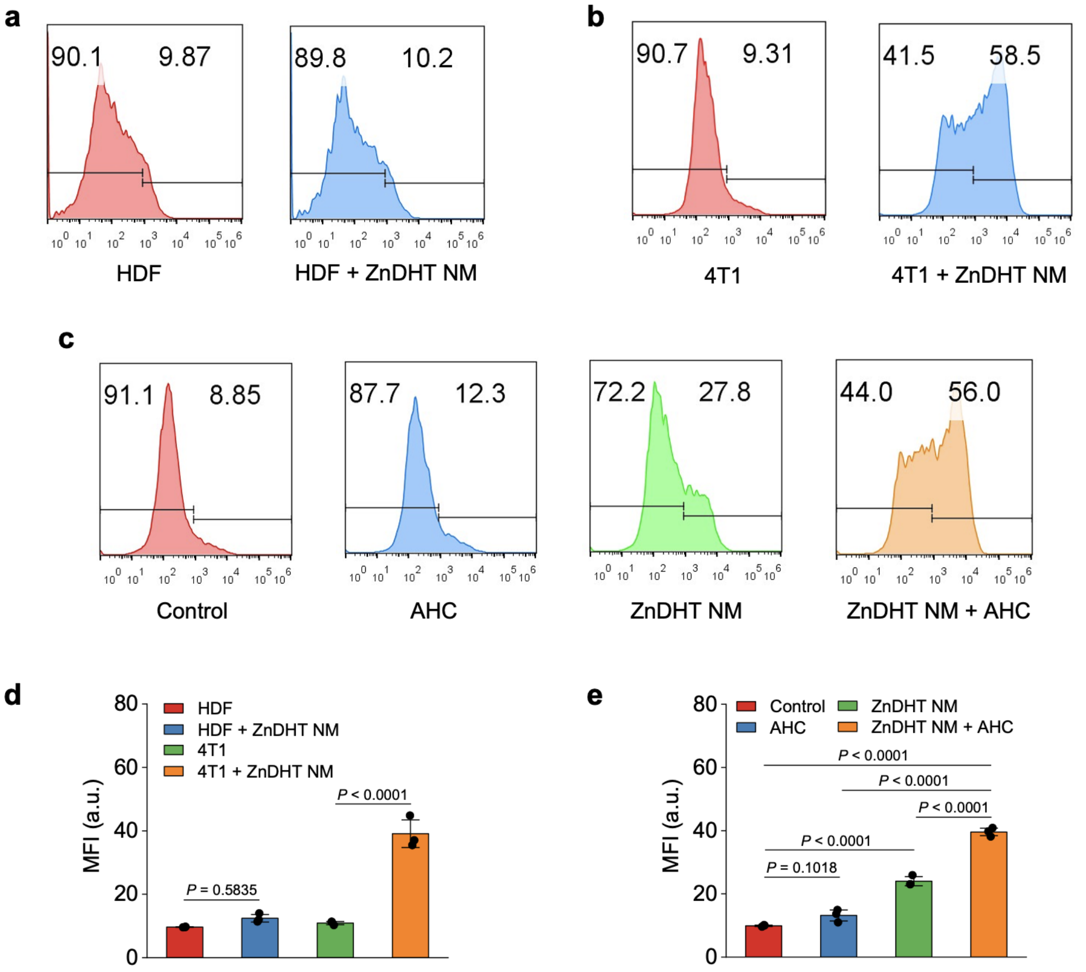

Figure S44. Flow cytometric analysis on the ROS produced in the ZnDHT NM-treated HDF cells (**a**) and 4T1 cells (**b**) or in 4T1 cells after different treatments (**c**) based on DCFH-DA as an intracellular ROS fluorescent probe. Corresponding green fluorescence intensities of different groups in **Fig. 5d** (**d**) and **Fig. 5e** (**e**). The flow cytometric analysis results are consistent with the fluorescence observation experiments. Data are expressed as mean ± s.e.m. (n = 3 independent experiments). Statistical significance was determined by one-way ANOVA with Tukey's post-hoc test.

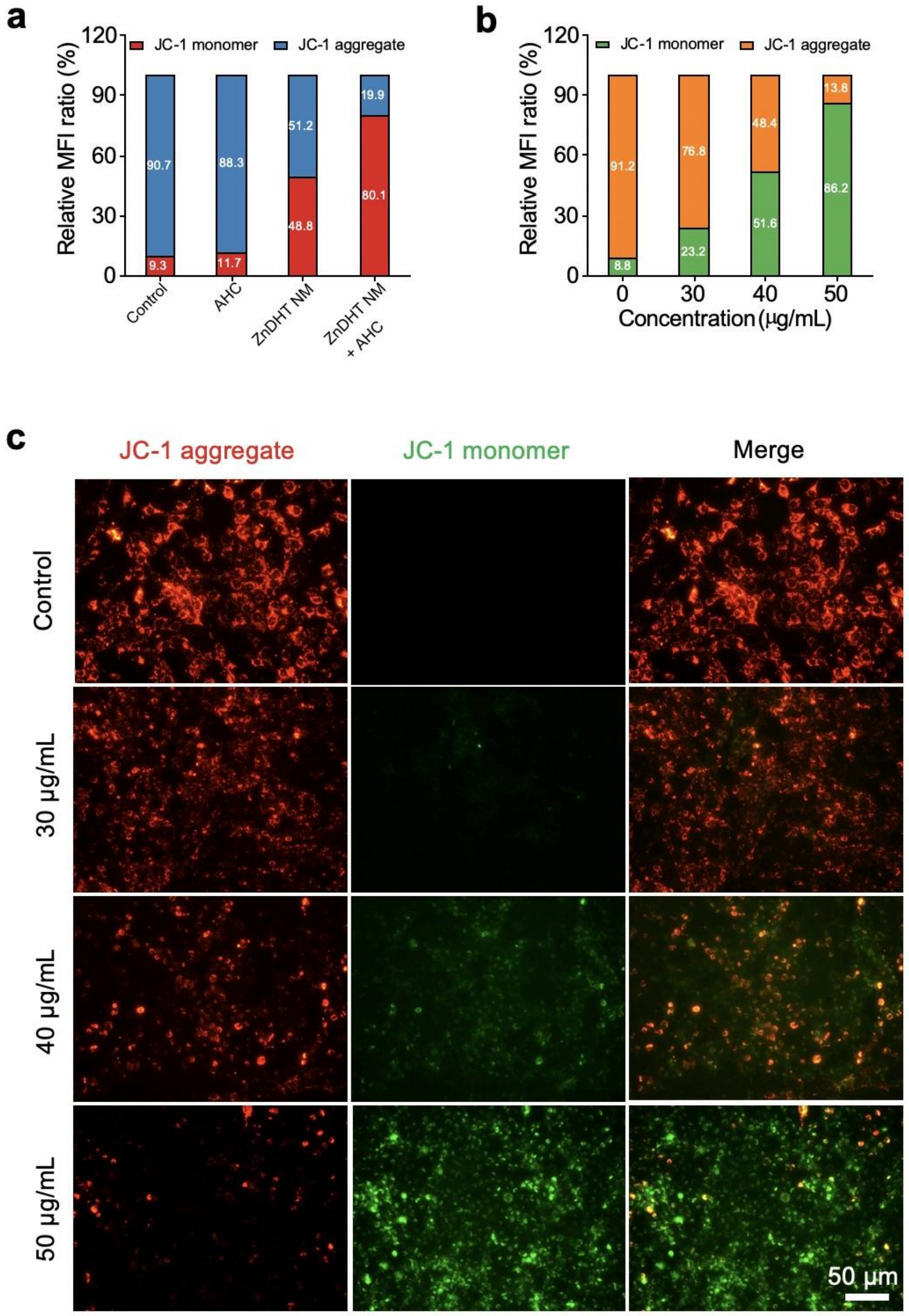

Figure S45. Relative fluorescence analysis of JC-1 staining of 4T1 cells with different treatments (**a**) or with incubation of different concentrations of ZnDHT NM (**b**). Fluorescence images of JC-1 staining of 4T1 cells with incubation of different concentrations of ZnDHT NM (**c**). The red fluorescent JC-1 aggregate indicates normal mitochondrial membrane potential, but the green fluorescent JC-1 monomer represents the depolarized mitochondrial membrane. As shown in figures, ZnDHT NM exhibited a dose-dependent damage effect on the mitochondria of 4T1 cells.

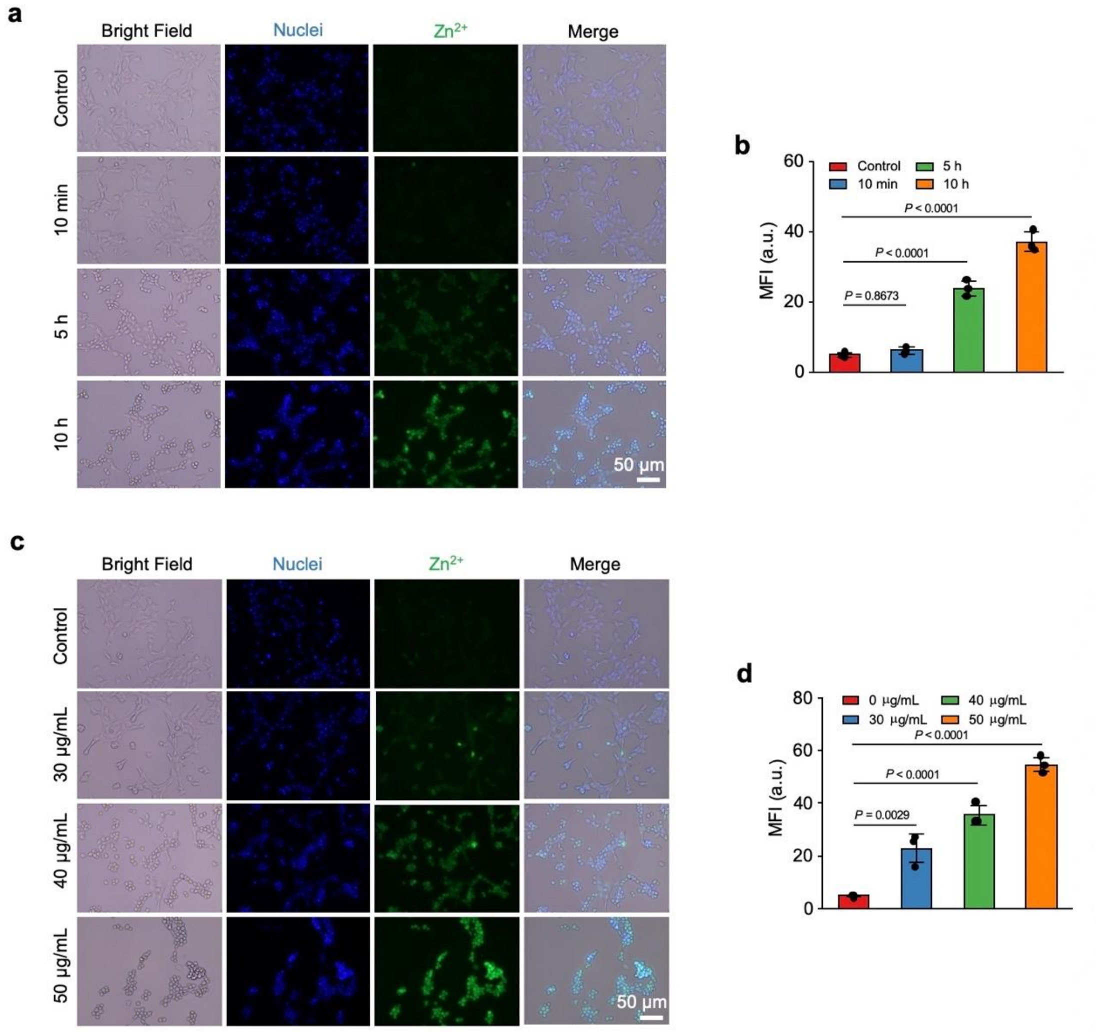

Figure S46. Fluorescence images presenting the intracellular Zn^{2+} (green fluorescence) of 4T1 cells after incubation of ZnDHT NM for different times (**a**) or with treatment of different concentrations of ZnDHT NM (**c**) based on Metal Fluor™ Zn-520 as an intracellular Zn^{2+} fluorescence probe, and corresponding green fluorescence intensities of different groups in **a** (**b**) and **c** (**d**). As the ZnDHT NM incubation time prolonged, the amount of Zn^{2+} inside the 4T1 cells continued to increase. In addition, the treatment with more ZnDHT NM led to an increased amount of Zn^{2+} inside the 4T1 cells. These results indicate that ZnDHT NM after cellular uptake could respond to the endogenous Fe^{3+} to release Zn^{2+}. Data are expressed as mean ± s.e.m. (n = 3 independent experiments). Statistical significance was determined by one-way ANOVA with Tukey's post-hoc test.

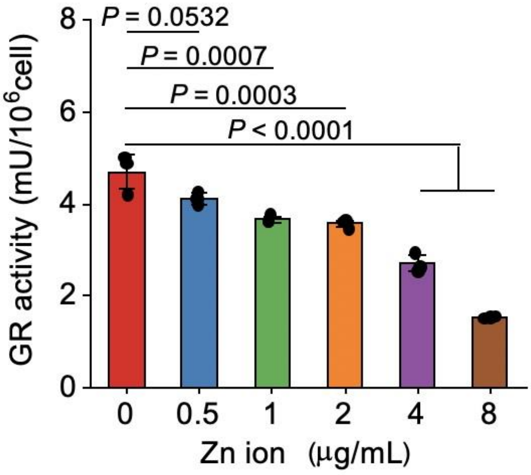

Figure S47. Cellular GR activity evaluation of 4T1 cells after treatment with different concentrations of Zn^{2+}. Zn^{2+} inhibited the GR activity of 4T1 cells in a dose-dependent manner, confirming the ability to disturb cellular antioxidant defense, in consistent with the previous studies [15]. Data are expressed as mean ± s.e.m. (n = 3 independent experiments). Statistical significance was determined by one-way ANOVA with Tukey's post-hoc test.

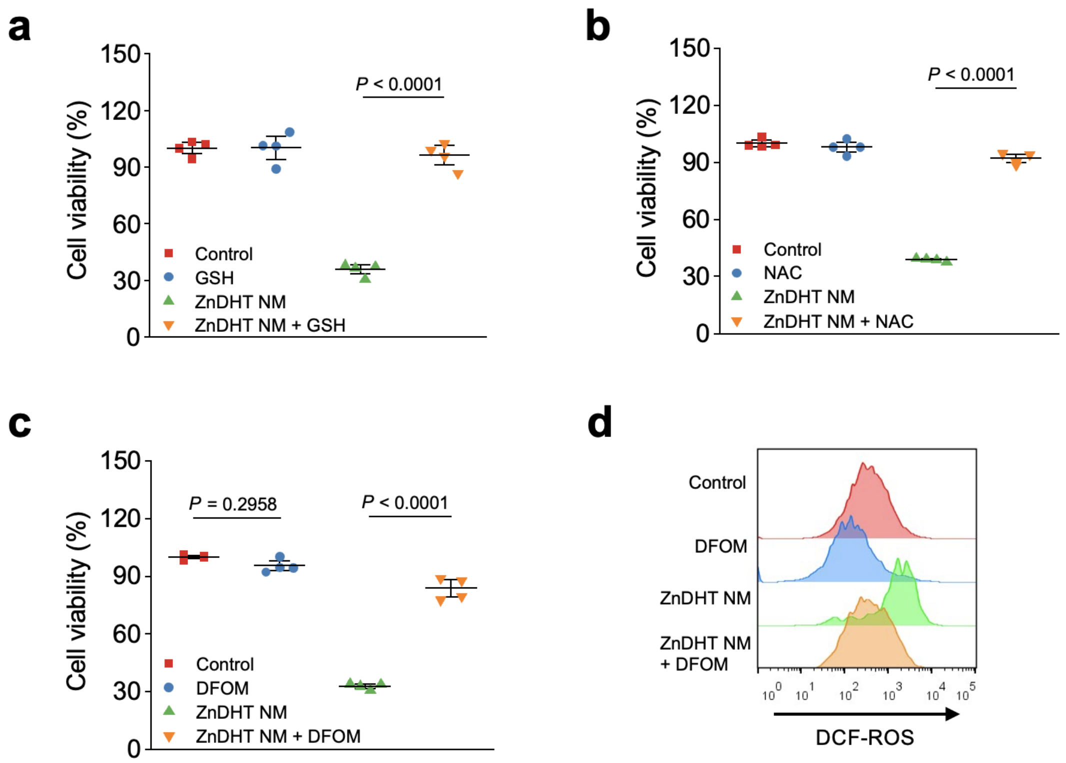

Figure S48. Cell viabilities of 4T1 cells after incubation with ZnDHT NM (40 μg/mL) and/or reduced glutathione (GSH, 2 mM) for 24 h (**a**). Cell viabilities of 4T1 cells after incubation with ZnDHT NM (40 μg/mL) and/or *N*-acetyl-L-cysteine (NAC, 2 mM) for 24 h (**b**). Cell viabilities of 4T1 cells after incubation with ZnDHT NM (40 μg/mL) and/or DFOM (200 μM) for 24 h (**c**). Flow cytometric analysis on the ROS produced in 4T1 cells after incubation with ZnDHT NM (40 μg/mL) and/or DFOM (200 μM) for 5 h based on DCFH-DA as an intracellular ROS fluorescent probe (**d**). DFOM significantly lowered the intracellular ROS production in the ZnDHT NM-treated cells, owing to the strong ability of DFOM on competing for the endogenous Fe^{3+} to form the redox-inert Fe-DFOM complex instead of to produce the redox-active ZnFeDHT NM. This may be the reason why DFOM could prevent ZnDHT NM from killing most of the treated 4T1 cells. Data are expressed as mean ± s.e.m. (*n* = 4 independent experiments). Statistical significance was determined by one-way ANOVA with Tukey's post-hoc test.

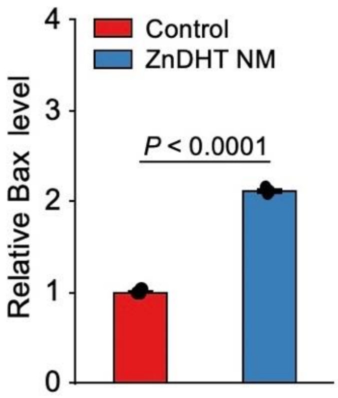

Figure S49. RT-qPCR analysis of the relative Bax gene expression in the ZnDHT NM-treated 4T1 cells. Data are expressed as mean ± s.e.m. (n = 3 independent experiments). Statistical significance was determined by two-tailed Student's t-test.

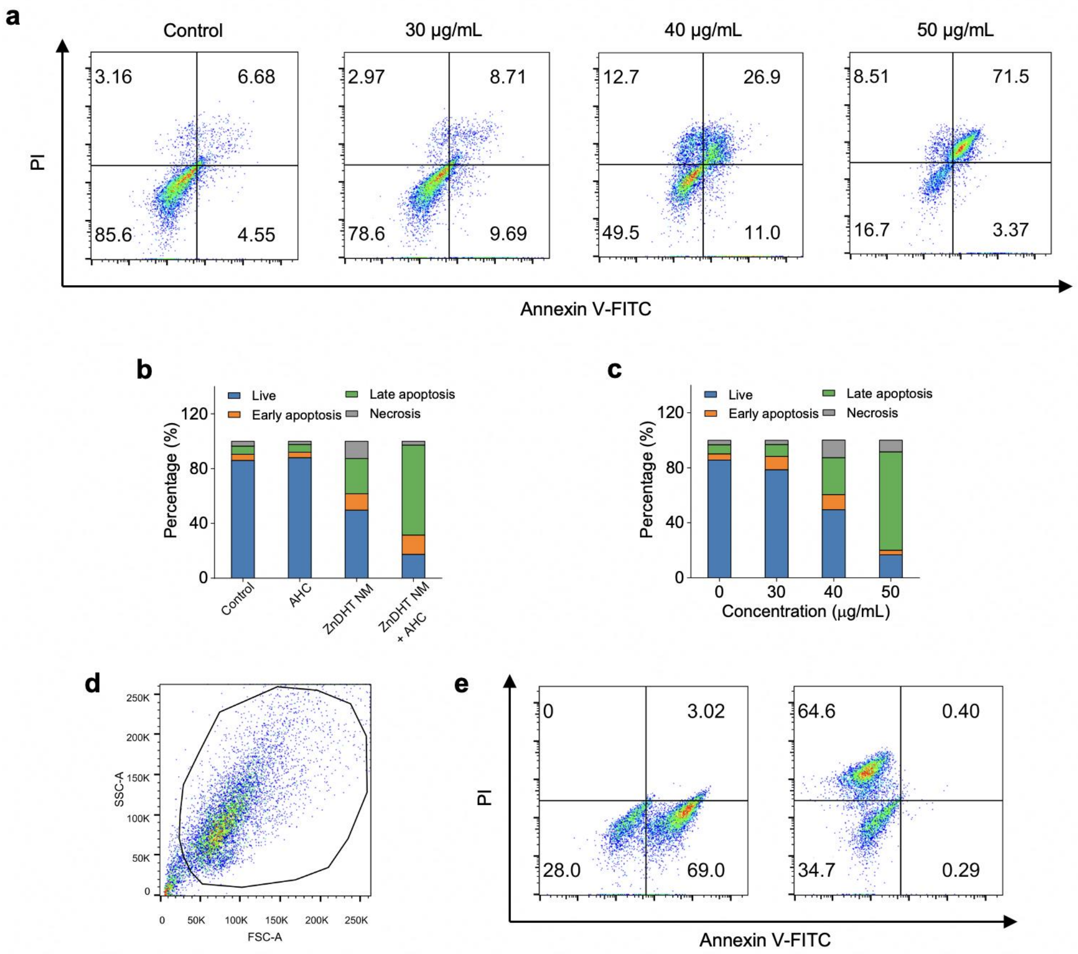

Figure S50. Flow cytometric analysis of Annexin V-FITC/PI staining of 4T1 cells with treatment of different concentrations of ZnDHT NM (**a**). Quantitative results in flow cytometric analysis of Annexin V-FITC/PI staining of 4T1 cells with different treatments (**b**) or with treatment of different concentrations of ZnDHT NM (**c**). Gating strategy to sort 4T1 cells for flow cytometric analyses in **Fig. 5s** and **Figure S50** (**d**). Flow cytometric analysis of single Annexin V-FITC (left) or single PI (right) staining of live/dead 4T1 cells (**e**).

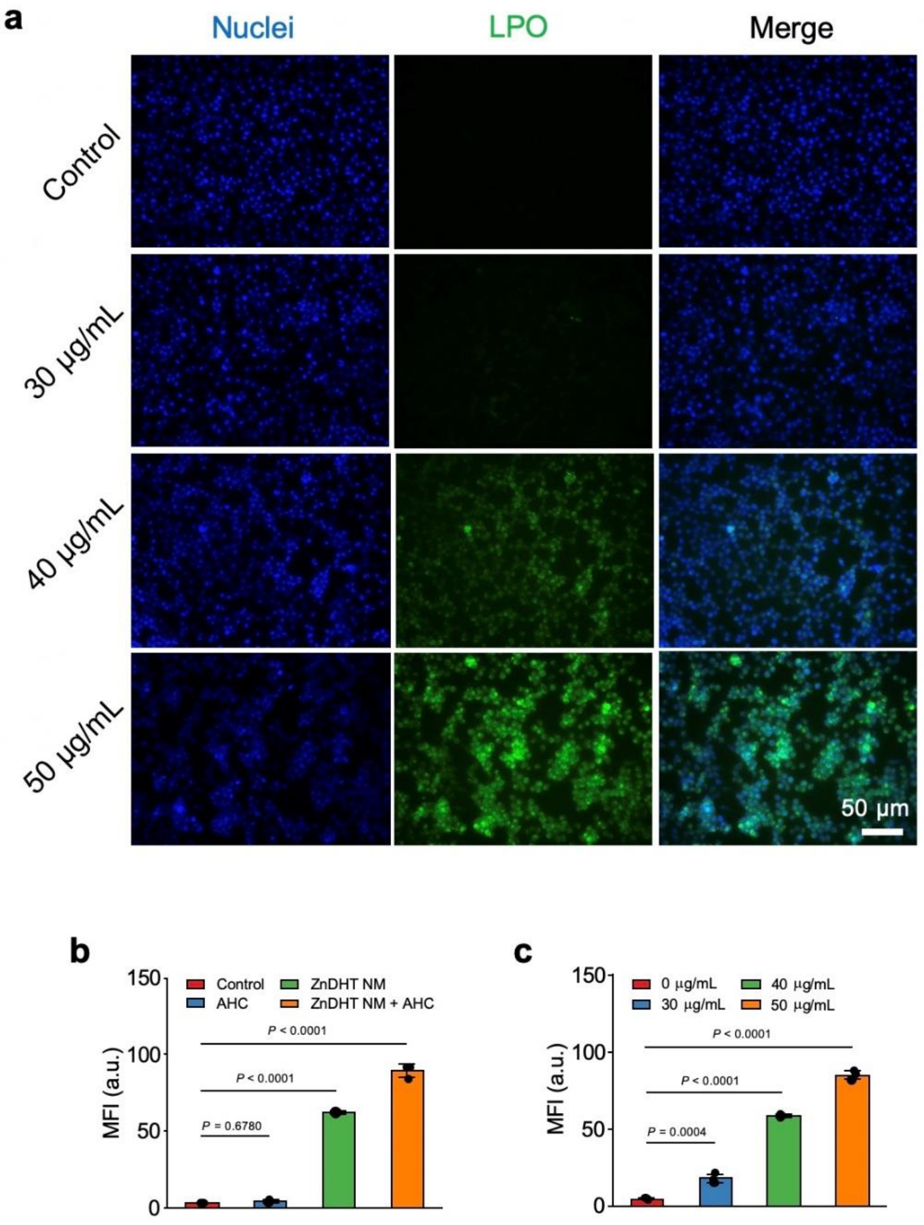

Figure S51. Fluorescence images of BODIPY™ 581/591 C11 staining of 4T1 cells with treatment of different concentrations of ZnDHT NM (**a**). The green fluorescence represents cellular LPO. Corresponding green fluorescence intensities of BODIPY™ 581/591 C11 staining of 4T1 cells with different treatments (**b**) or with treatment of different concentrations of ZnDHT NM (**c**). Data are expressed as mean ± s.e.m. (*n* = 3 independent experiments). Statistical significance was determined by one-way ANOVA with Tukey's post-hoc test.

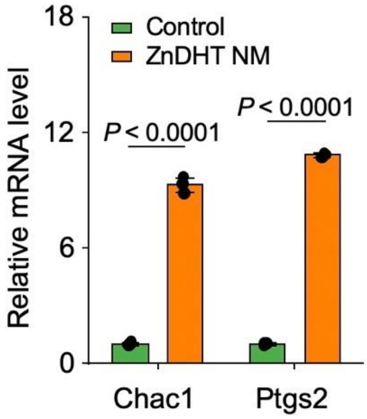

Figure S52. RT-qPCR analysis of the relative Chac1 and Ptgs2 gene expressions in the ZnDHT NM-treated 4T1 cells. The upregulation of Chac1 and Ptgs2 genes has been identified as the important genetic hallmark of ferroptosis [16]. Accordingly, the results indicate that ZnDHT NM activated the ferroptosis of tumor cells. Data are expressed as mean ± s.e.m. (*n* = 3 independent experiments). Statistical significance was determined by two-tailed Student's *t*-test.

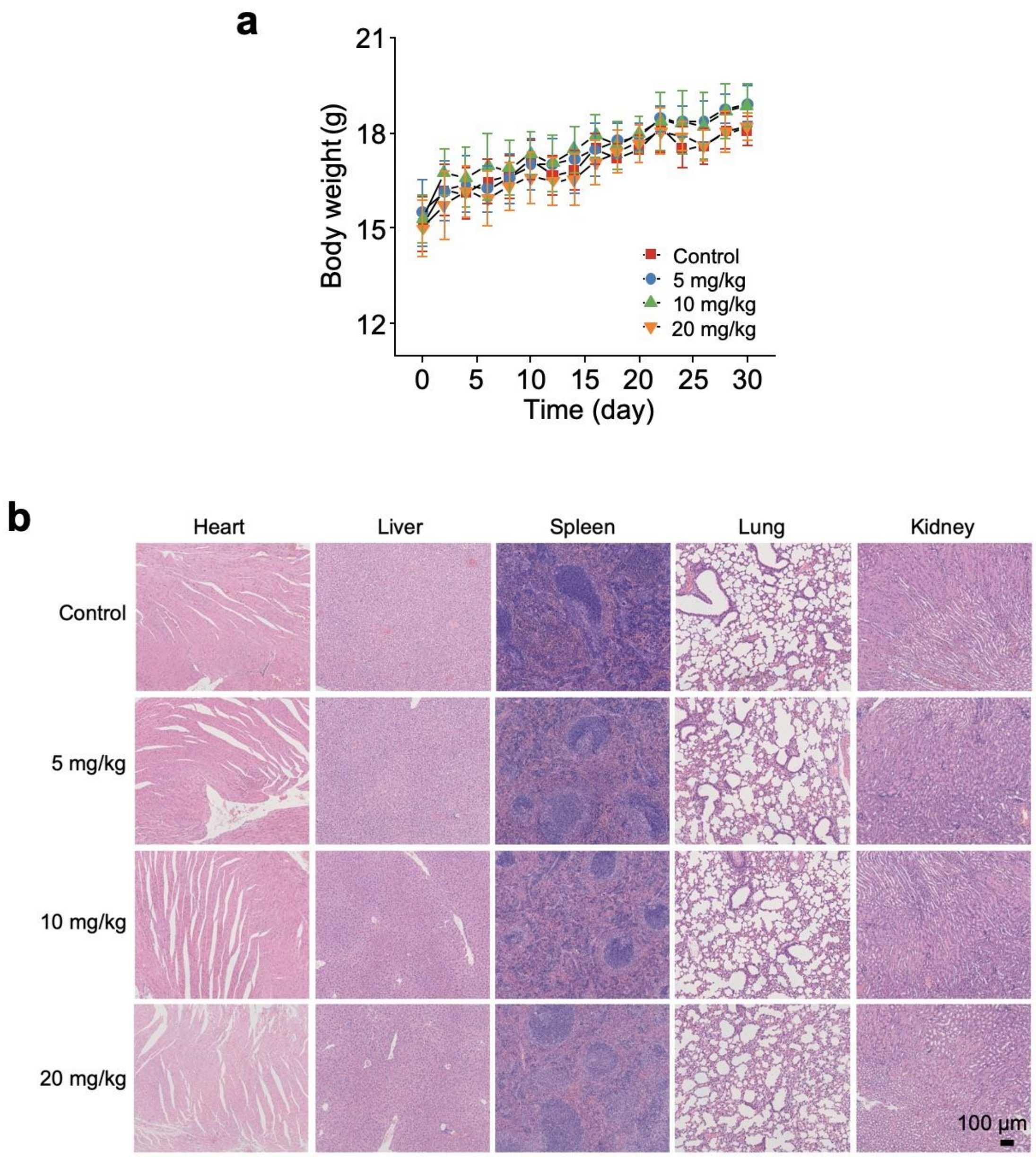

Figure S53. Body weight variations of the healthy mice post systemic administration of different concentrations of ZnDHT NM (**a**), exhibiting no significant fluctuations in body weight compared to the control. Representative images of H&E staining of the tissues (including heart, liver, spleen, lung, and kidney) obtained from the healthy mice on day 30 post systemic administration of different concentrations of ZnDHT NM (**b**). The histopathological evaluation after administrations shows no noticeable abnormality of these main organs in the ZnDHT NM-treated groups in comparison to the control, demonstrating the *in vivo* biocompatibility of ZnDHT NM. Data are expressed as mean ± s.e.m. (n = 5 independent experiments).

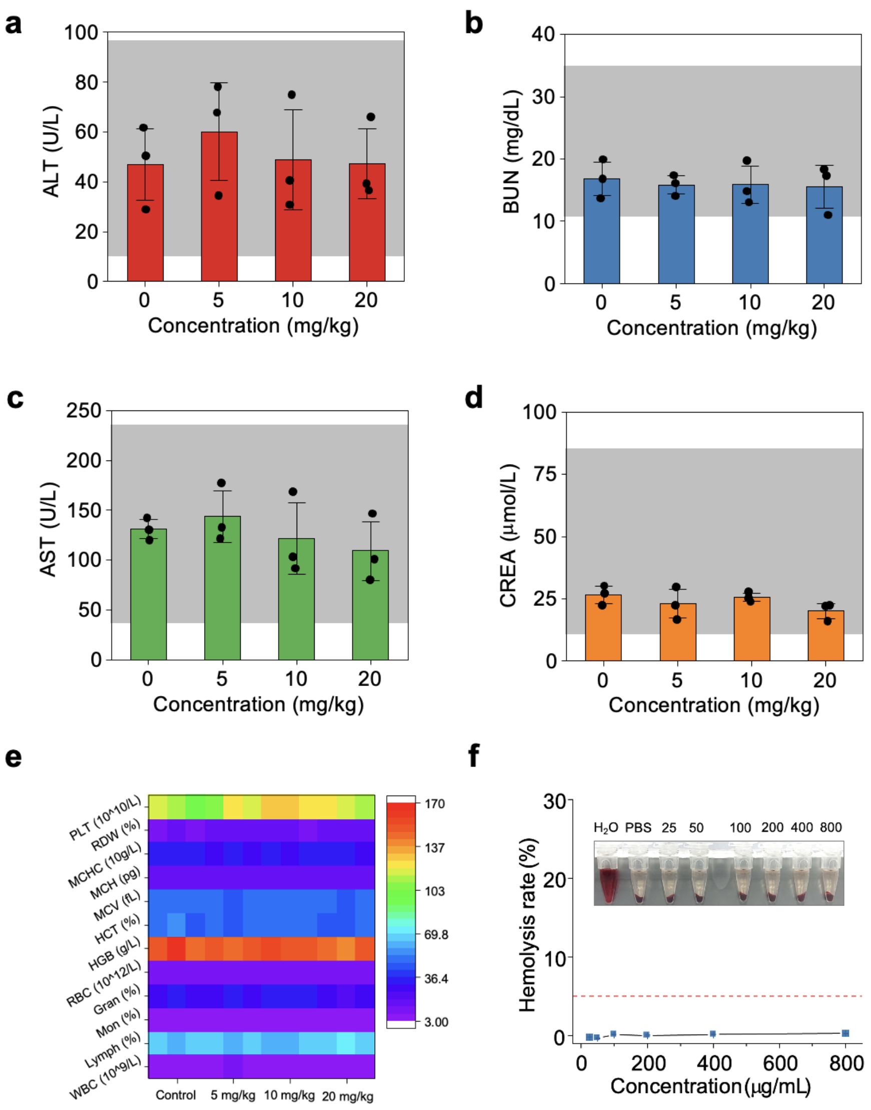

Figure S54. Analysis of the liver and kidney function indexes including alanine aminotransferase (ALT) (**a**), urea nitrogen (BUN) (**b**), aspartate aminotransferase (AST) (**c**), and creatinine (CREA) (**d**), and the blood-related parameters (**e**) of the healthy mice on day 30 post systemic administration of different concentrations of ZnDHT NM. Lighter gray bars represent the range of normal values obtained from the healthy mice. All these indicators in the ZnDHT NM-treated groups were presented to be no significant fluctuations compared to the control, proving the good blood safety of ZnDHT NM. Hemolysis assay for ZnDHT NM at different concentrations (**f**). Red dashed line indicates the position of 5% hemolysis rate. Insert: the photograph of these tested solutions after centrifugation. Data are expressed as mean ± s.e.m. (*n* = 3 independent experiments).

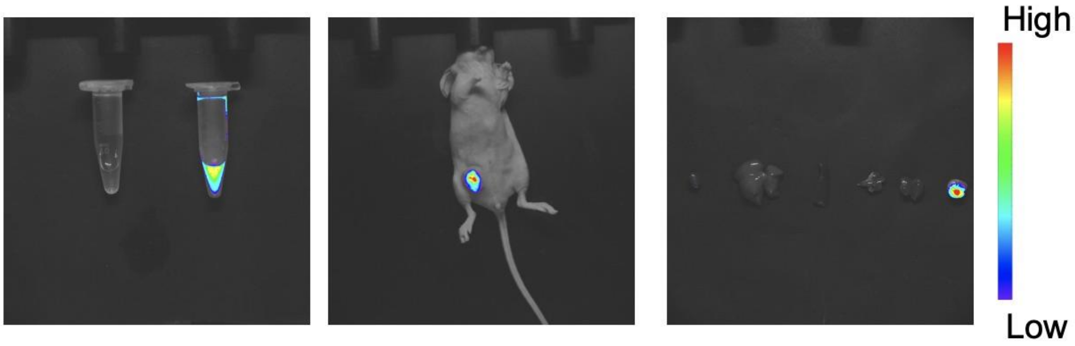

Figure S55. Fluorescence image of saline and Cy5-labeled ZnDHT NM in the tubes, *in vivo* animal fluorescence image of the tumor-bearing mouse post interventional treatment of Cy5-labeled ZnDHT NM at 24 h, and *ex vivo* fluorescence image of the main organs (from left to right: heart, liver, spleen, lung, kidney) and tumor of the tumor-bearing mouse post interventional treatment of Cy5-labeled ZnDHT NM at 24 h.

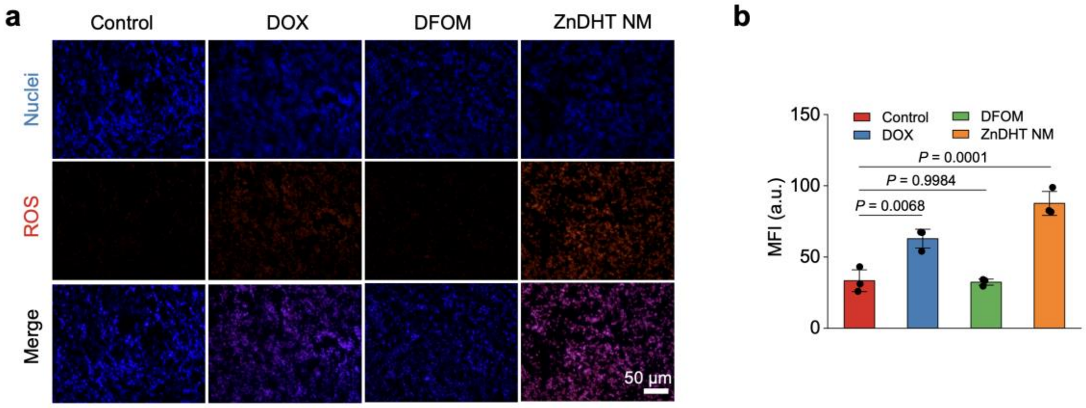

Figure S56. Representative images of ROS staining (red fluorescence) of the tumor tissues obtained from the 4T1 tumor-bearing mice after different interventional treatments for 12 h (**a**), and corresponding red fluorescence intensities of different groups (**b**). Compared with the control group and DFOM group, the DOX group produced a certain amount of ROS in the tumor tissue, due to the intricate mechanisms of DOX in inducing ROS generation [17]. More importantly, ZnDHT NM showed the highest efficacy on inducing ROS generation in the tumor tissue. Data are expressed as mean ± s.e.m. (*n* = 3 independent experiments). Statistical significance was determined by one-way ANOVA with Tukey's post-hoc test.

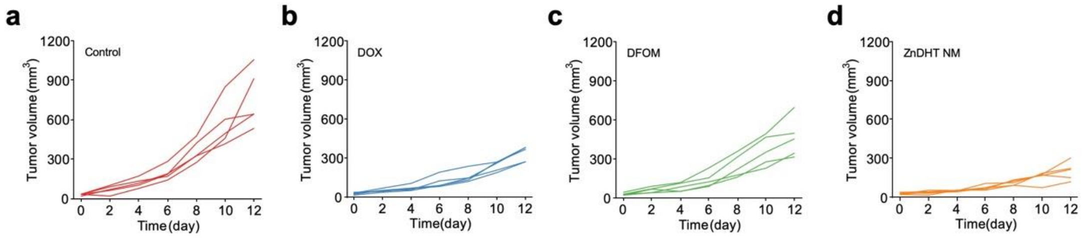

Figure S57. Individual tumor growth of the 4T1 tumor-bearing mice in the different treatment groups (n = 5), including control (**a**), DOX (**b**), DFOM (**c**), and ZnDHT NM (**d**).

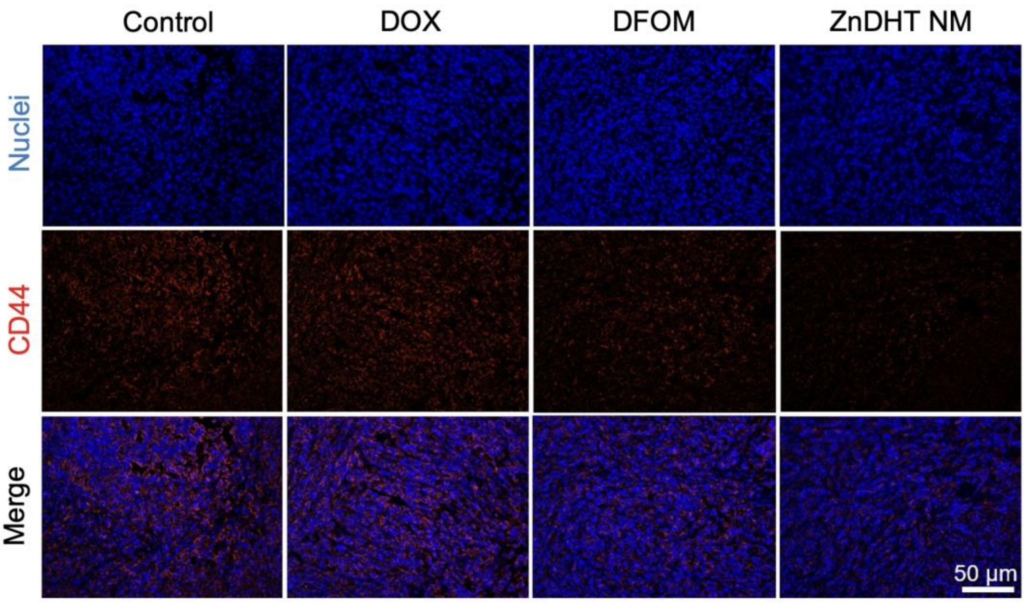

Figure S58. Representative images of CD44 immunofluorescent staining of the tumor tissues obtained from the 4T1 tumor-bearing mice after different treatments.

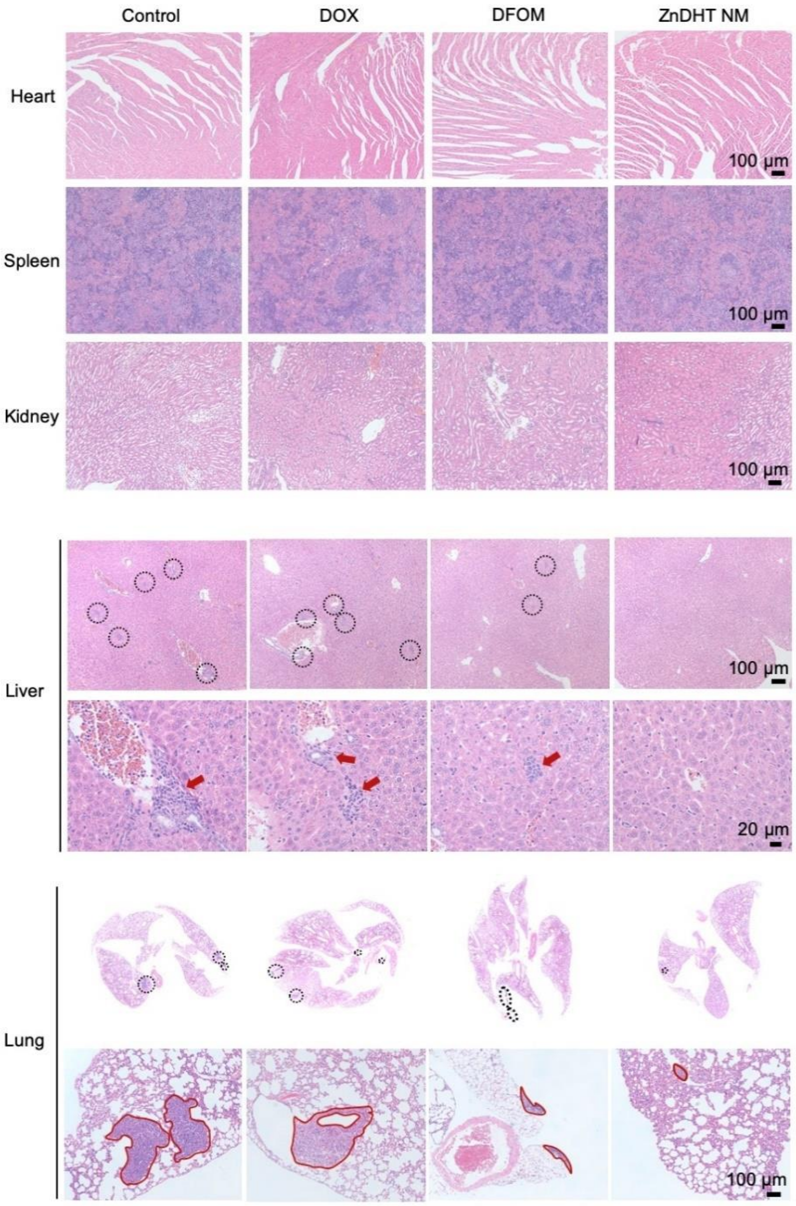

Figure S59. Representative images of H&E staining of the normal tissues (including heart, liver, spleen, lung, and kidney) obtained from the 4T1 tumor-bearing mice after different treatments. Black circles represent the metastatic tumors in the livers and lungs. Red arrows and lines indicate the positions of amplified tumor metastatic nodules. As shown in figures, the livers and lungs of the 4T1 tumor-bearing mice after the tumor orthotopic inoculation for 12 days presented noticeable tumor metastatic nodules. In comparison to the DOX and DFOM groups, the ZnDHT NM group showed the most effective inhibition on tumor liver and lung metastasis. In addition, no significant organ lesion and inflammation were found in the ZnDHT NM group, indicating the biosafety of treatment of ZnDHT NM.

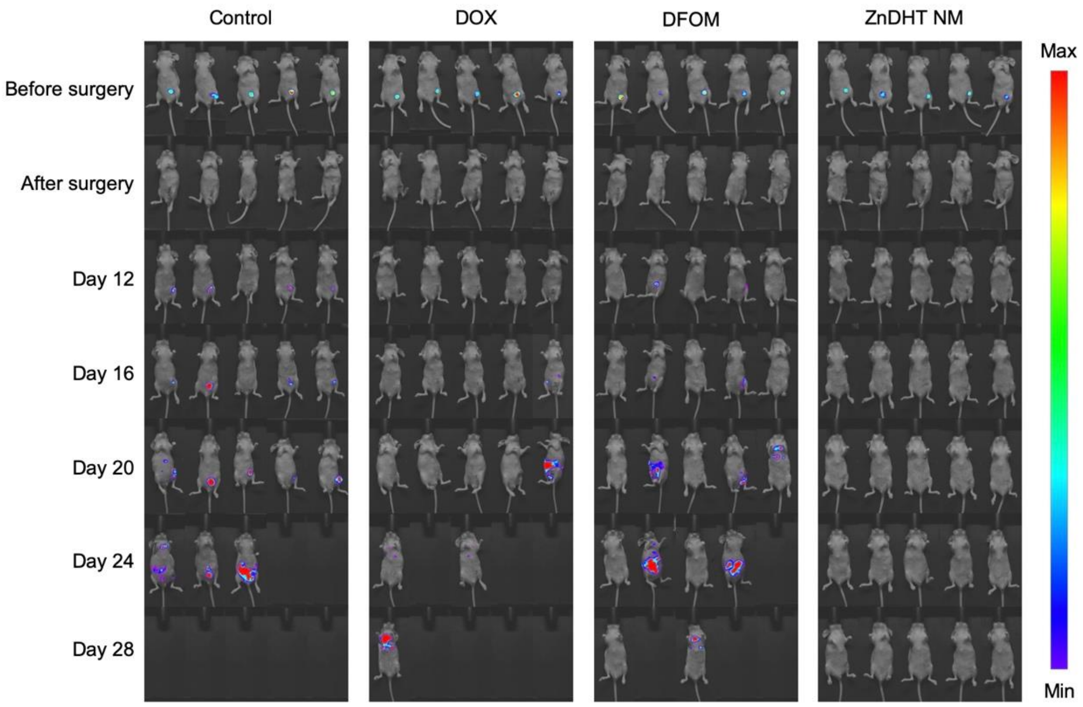

Figure S60. Bioluminescence images of the surgically treated luciferase-labeled 4T1 tumor-bearing mice at different days post different interventional treatments (*n* = 5). The empty spaces indicate the death of the mice.

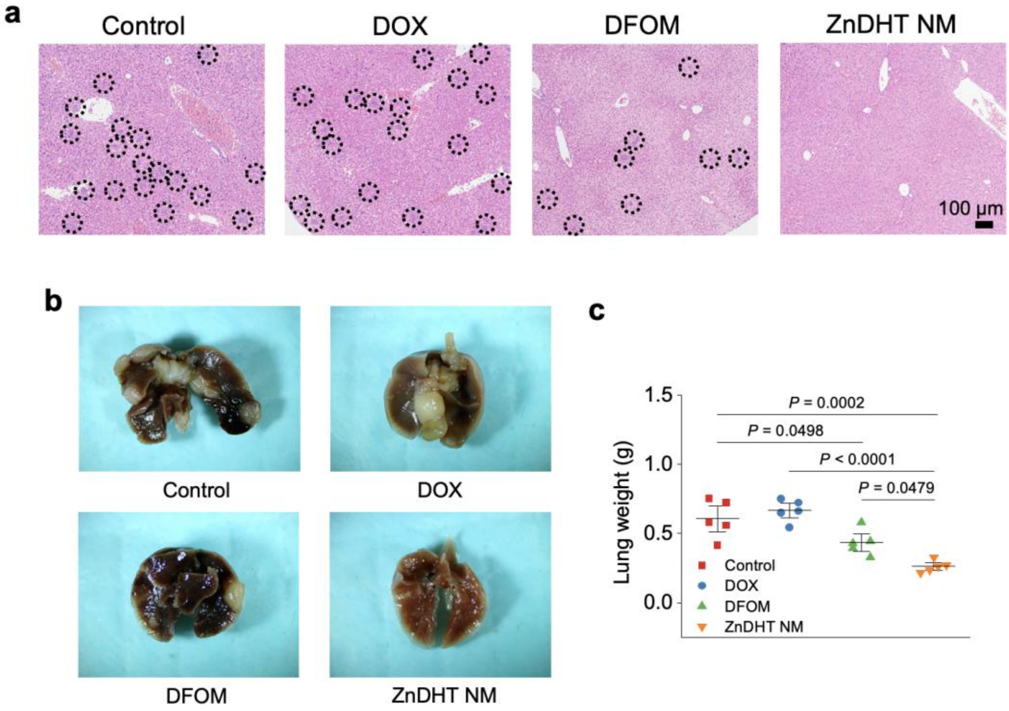

Figure S61. Representative images of H&E staining of the liver tissues obtained from the surgically treated 4T1 tumor-bearing mice on day 22 post different interventional treatments (**a**). Black circles indicate the positions of metastatic tumors in the livers. Photographs (**b**) and weights (**c**) of the lungs obtained from the surgically treated 4T1 tumor-bearing mice on day 22 post different interventional treatments. The lighter lung weight means the relatively less tumor lung metastasis [18]. Data are expressed as mean ± s.e.m. (*n* = 5 independent experiments). Statistical significance was determined by one-way ANOVA with Tukey's post-hoc test.

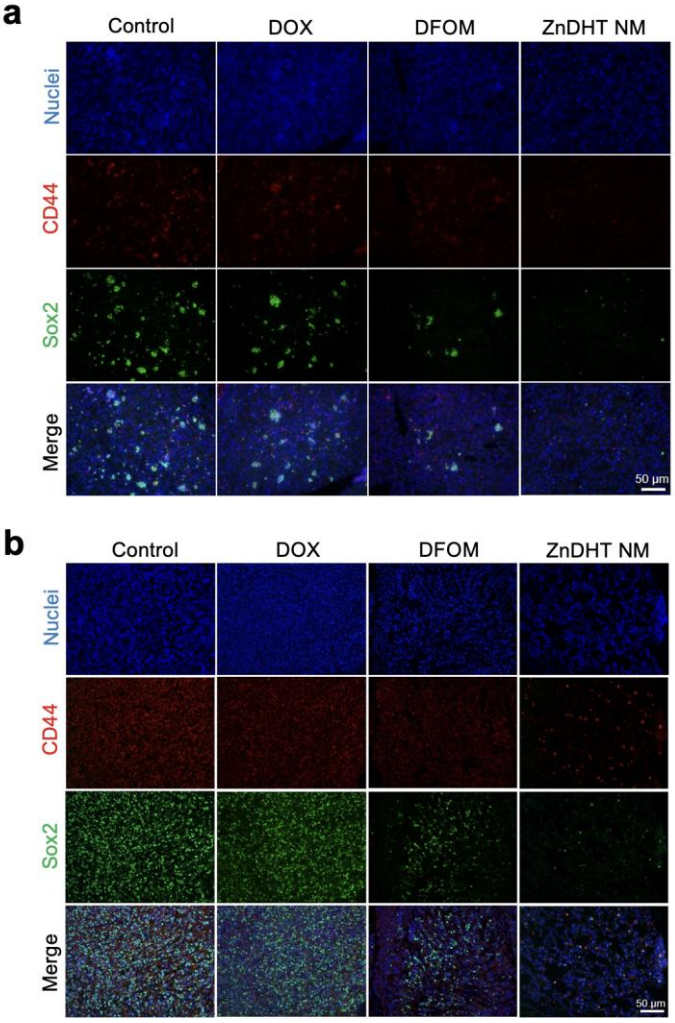

Figure S62. Representative images of CD44/Sox2 immunofluorescence co-staining of the liver (**a**) and lung (**b**) tissues obtained from the surgically treated 4T1 tumor-bearing mice on day 22 post different interventional treatments. The red/green co-labeled regions represent the metastatic tumor cells with high stemness in the livers and lungs.

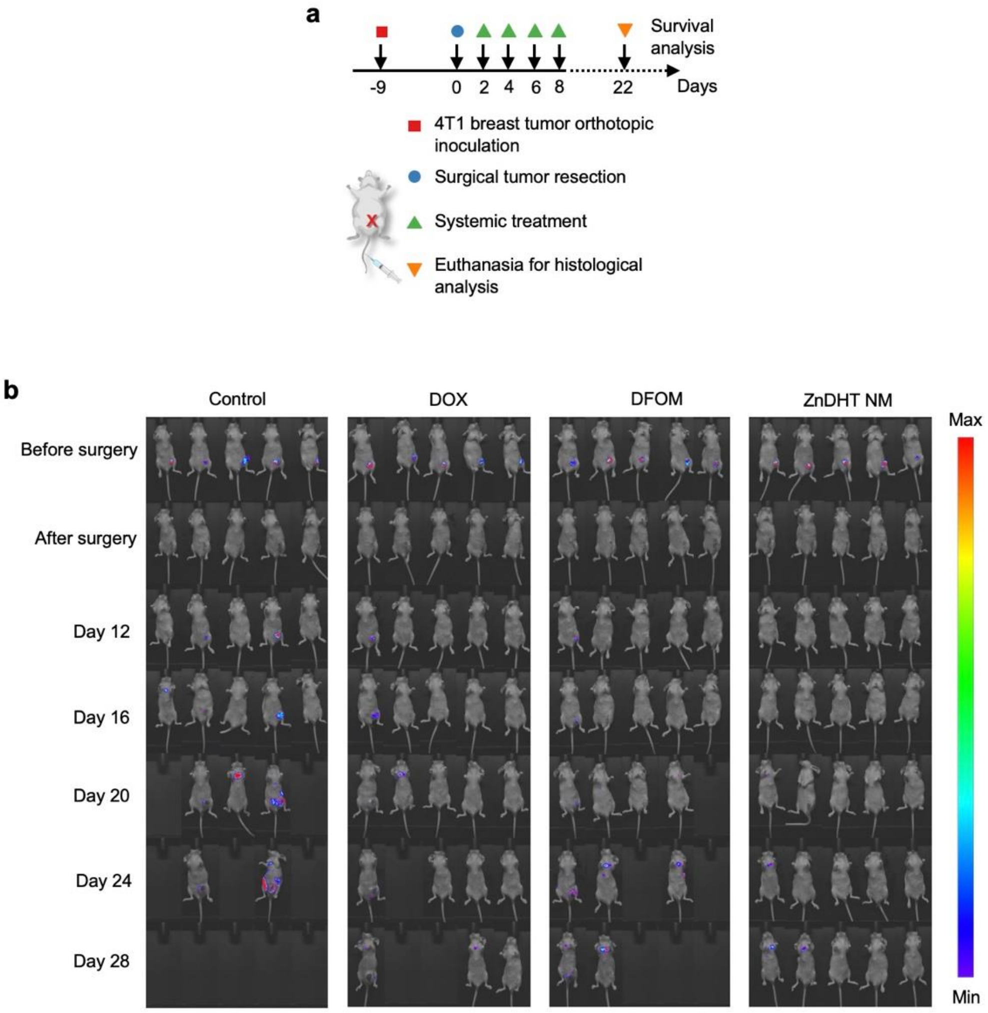

Figure S63. Illustration of experimental protocols for systemic treatments on mice after surgical orthotopic 4T1 breast tumor resection (**a**). Bioluminescence images of the surgically treated luciferase-labeled 4T1 tumor-bearing mice at different days post different systemic treatments (n = 5) (**b**). The empty spaces indicate the death of the mice.

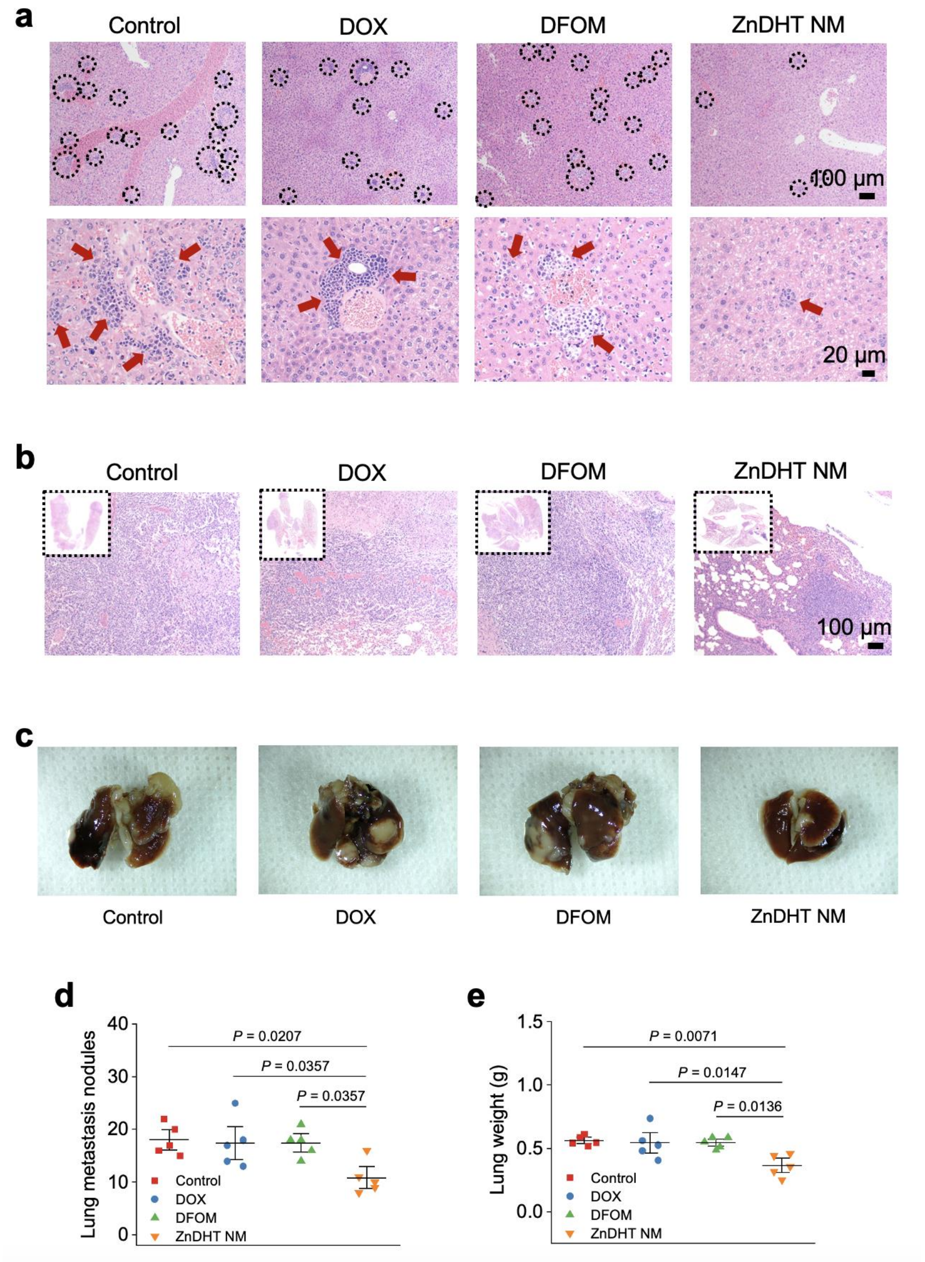

Figure S64. Representative images of H&E staining of the liver (**a**) and lung (**b**) tissues obtained from the surgically treated 4T1 tumor-bearing mice on day 22 post different systemic treatments. Black circles represent the metastatic tumors in the livers, and red arrows indicate the positions of amplified metastatic tumors (**a**). The insert images show the entire lung tissues (**b**). Photographs (**c**), lung metastasis nodule counts (**d**), and weights (**e**) of the lungs obtained from the surgically treated 4T1 tumor-bearing mice on day 22 post different systemic treatments. Data are expressed as mean ± s.e.m. (n = 5 independent experiments). Statistical significance was determined by one-way ANOVA with Tukey's post-hoc test.

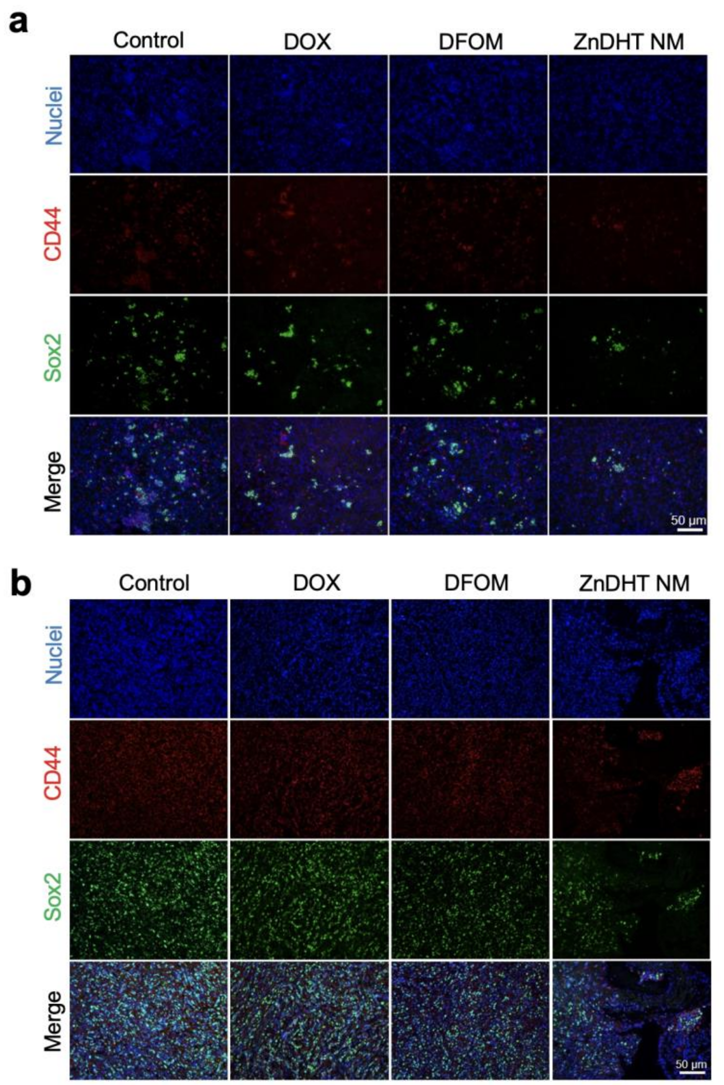

Figure S65. Representative images of CD44/Sox2 immunofluorescence co-staining of the liver (**a**) and lung (**b**) tissues obtained from the surgically treated 4T1 tumor-bearing mice on day 22 post different systemic treatments. The red/green co-labeled regions represent the metastatic tumor cells with high stemness in the livers and lungs.

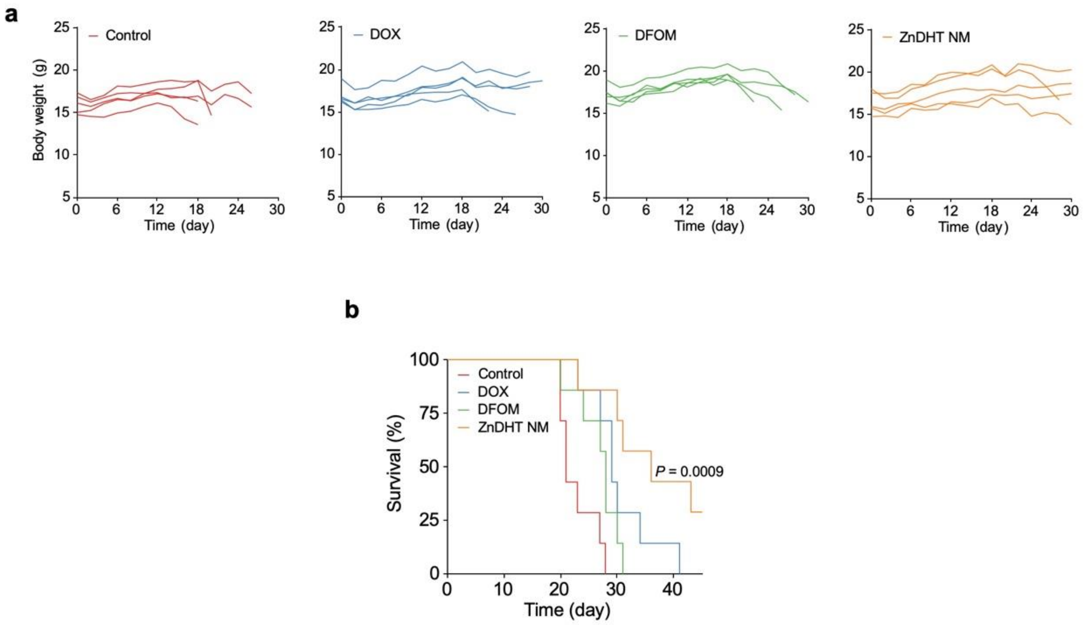

Figure S66. Individual body weight variation of the surgically treated 4T1 tumor-bearing mice within 30 days post different systemic treatments (*n* = 5) (**a**). Survival curves of the surgically treated 4T1 tumor-bearing mice after different systemic treatments (*n* = 7) (**b**), which were recorded in another parallel experiment of **a**. Statistical significance was determined by log-rank (Mantel-Cox) test.

Supplementary Tables

Supplementary Table 1. Calculated bond dissociation energies (BDEs) between DHT and different metal ions in the three coordinated modes.

Metal ion	Bond dissociation energy (a.u.)		
	**M**-O carboxylate	**M**-O phenate	O-**M**-O bridge-type
K^{+}	0.025	0.035	0.032
Na^{+}	0.038	0.058	0.046
Ca^{2+}	0.153	0.167	0.123
Mg^{2+}	0.264	0.264	0.203
Zn^{2+}	0.262	0.306	0.276
Co^{2+}	0.413	0.419	0.402
Cu^{2+}	0.429	0.432	0.428
Mn^{2+}	0.322	0.381	0.353
Fe^{2+}	0.292	0.362	0.320
Mo^{3+}	0.726	0.759	0.751
Al^{3+}	0.933	0.857	0.873
Cr^{3+}	0.938	0.953	0.952

Ga^{3+}	1.014	0.960	1.009
**Fe^{3+}**	**1.079**	**1.080**	**1.110**

From the data, it can be observed that the coordination structure between DHT and Fe^{3+} has the highest BDE compared to other metal ions, indicating that DHT is most inclined to bind to Fe^{3+}. Specifically, due to the order of BDE being Fe-O carboxylate < Fe-O phenate < O-Fe-O bridge-type, the coordination between DHT and Fe^{3+} in the bridge-type mode is thermodynamically more favorable.

Supplementary Table 2. EXAFS fitting parameters at the Fe K-edge for various samples.

Sample	Shell	***CN***[a]	***R*** **(Å)**[b]	σ^2 **(Å^2)**[c]	ΔE_0 **(eV)**[d]	***R*** **factor**
Fe foil	Fe-Fe1	8*	2.47	0.0049	5.93	0.0046
	Fe-Fe2	6*	2.85	0.0056	5.80	
Fe_2O_3	Fe-O	6.8	1.97	0.0115	-5.66	0.0124
	Fe-Fe1	7.6	2.98	0.0084	0.77	
	Fe-Fe2	3.2	3.44	0.0036	13.46	
ZnFeDHT	Fe-O	5.9	1.96	0.0098	-4.00	0.0018
	Fe-Fe	3.2	3.05	0.0123	1.57	

[a]*CN*: coordination number. [b]*R*: distance between absorber and backscatter atoms. [c]σ^2: Debye-Waller factor to account for both thermal and structural disorders. [d]ΔE_0: inner potential correction. *R* factor indicates the goodness of the fit. An *R* factor less than 0.02 means a good fit between the data and theory. S_0^2 was fixed to 0.74.

Supplementary Table 3. Calculated bond dissociation energies (BDEs) between DHT and different trivalent metal ions in the optimized hexacoordinated mode.

**Metal ion**	**Bond dissociation energy (a.u.)**
Mo^{3+}	1.225
Al^{3+}	1.281
Cr^{3+}	1.390
Ga^{3+}	1.274
**Fe^{3+}**	**1.499**

Supplementary Table 4. Calculated atomic charges of DHT and different coordination structures between DHT and Fe^{3+}.

Coordinated mode	Charge distribution
DHT molecule	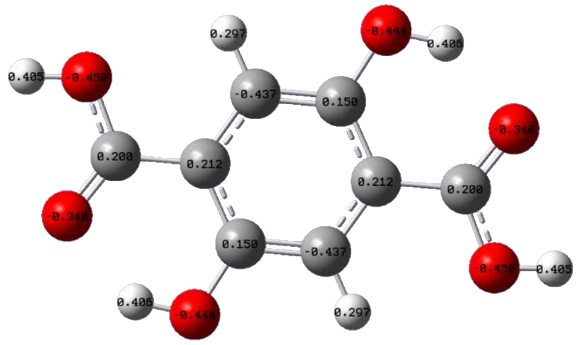
Fe-O carboxylate	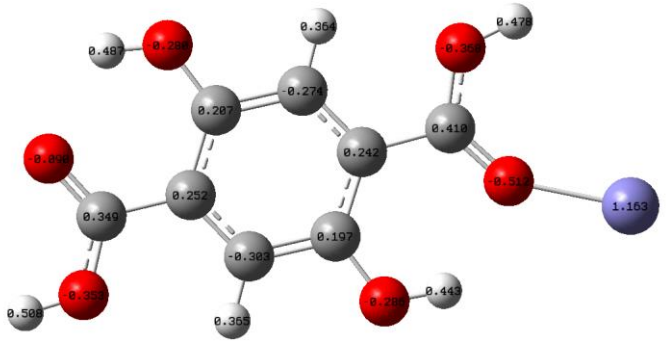

Fe-O phenate

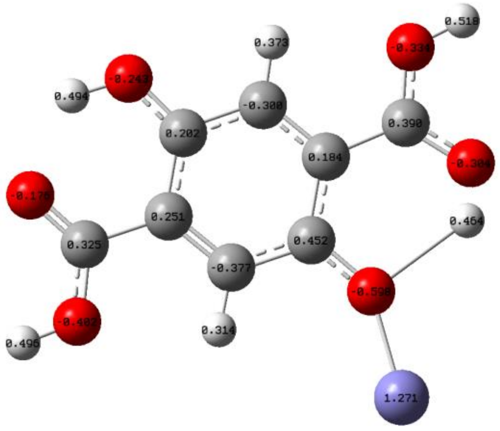

O-Fe-O bridge-type

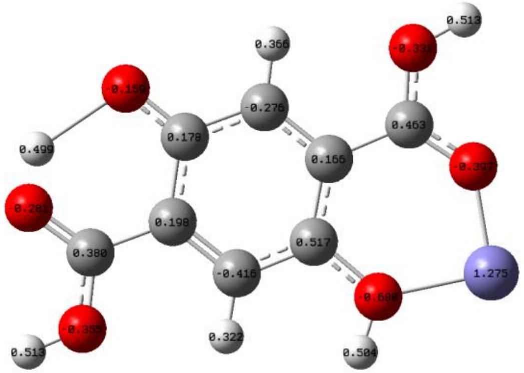

Hexacoordinated conformation	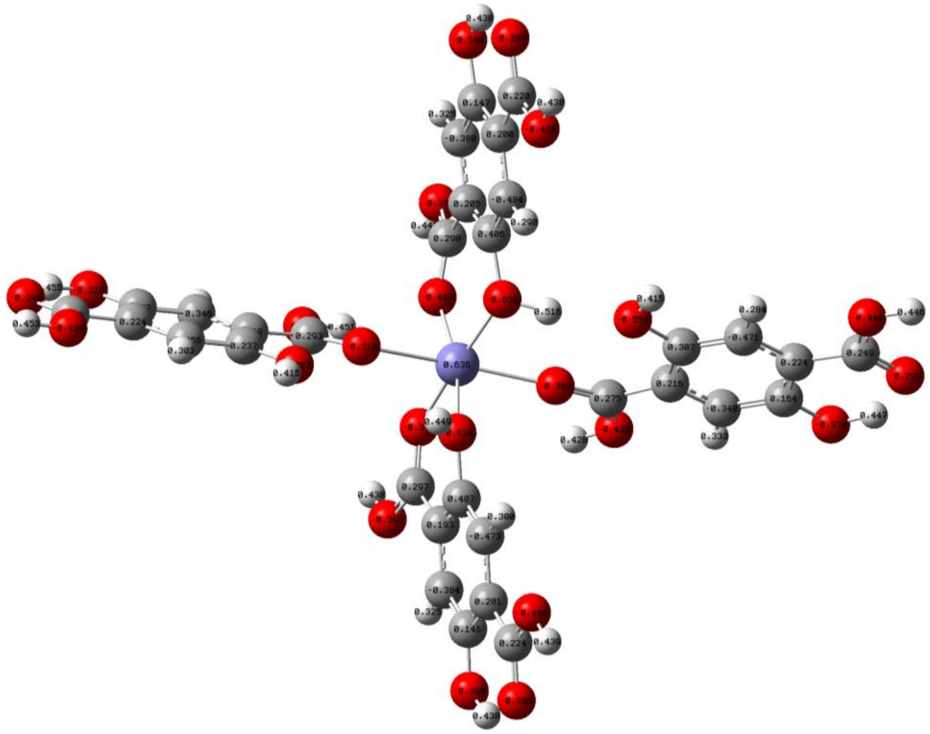

The calculated atomic charge distributions display that, in comparison to the original DHT molecule, after coordinating with Fe^{3+}, partial electrons have transferred from other atoms of DHT to the coordinated O and Fe atoms, resulting in the Fe atom being more negative. In the optimized hexacoordinated Fe-DHT conformation, the coordinated Fe atom is significantly more negative than that in the other coordinated modes, demonstrating the promoted electron transfer toward Fe atom in such structure.

Supplementary Table 5. Calculated LUMO and HOMO orbitals of different species.

Species	LUMO and corresponding energy (a.u.)	HOMO and corresponding energy (a.u.)	Energy gap (a.u.)
DHT	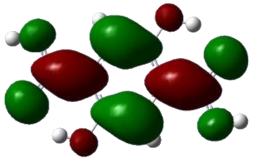 -0.111	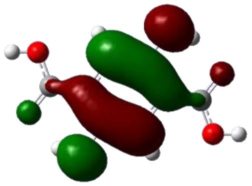 -0.235	0.124
Fe^{3+}	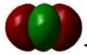 -1.561	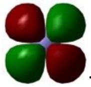 -1.717	0.156
DFOM	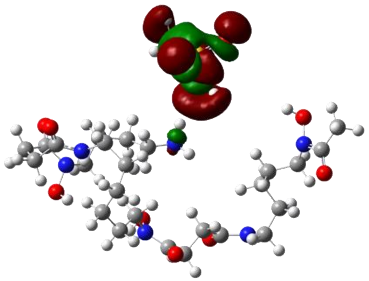 -0.113	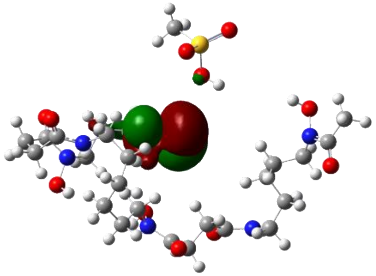 -0.218	0.105

Supplementary Table 6. Calculated LUMO and HOMO orbitals of different coordination structures between DHT and Fe^{3+}.

Coordinated mode	LUMO and corresponding energy (a.u.)	HOMO and corresponding energy (a.u.)	Energy gap (a.u.)
Fe-O carboxylate	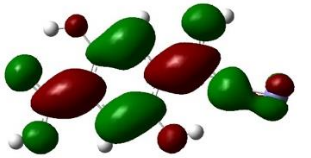 -0.542	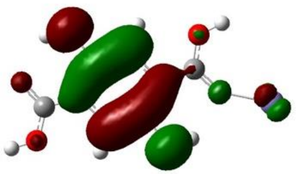 -0.675	0.132
Fe-O phenate	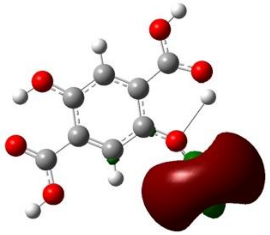 -0.577	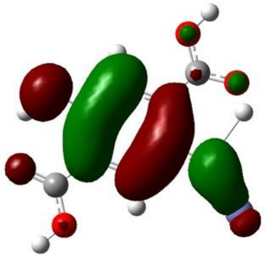 -0.694	0.117
O-Fe-O bridge-type	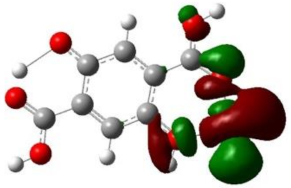 -0.596	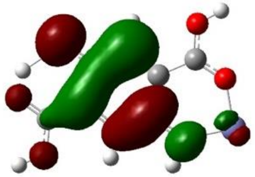 -0.694	0.098

Hexacoordinated conformation	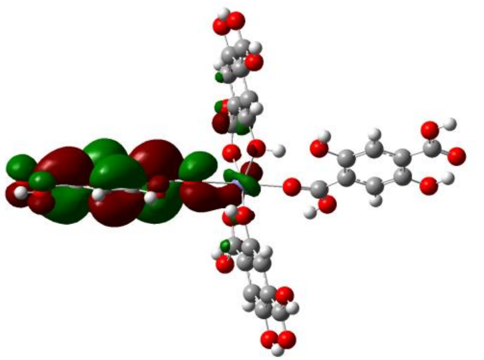 -0.384	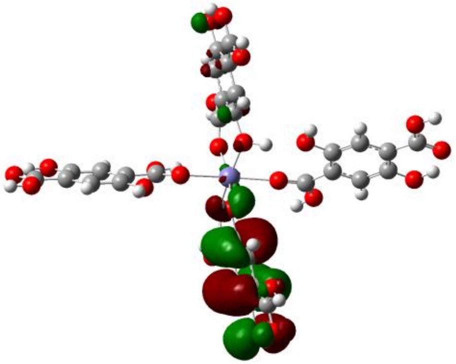 -0.498	0.115

These results show that the HOMOs of the coordination structures between DHT and Fe^{3+} (Fe-O carboxylate, Fe-O phenate, and O-Fe-O bridge-type) are localized on the benzene ring of DHT. The LUMOs are centered on the metal for the Fe-O phenate and O-Fe-O bridge-type coordination structures, while the LUMO of the Fe-O carboxylate coordination structure is less localized on the metal. Therefore, from a thermodynamic perspective, activated electron transfer from the DHT ligand to the coordinated Fe atom can be more likely to occur in the Fe-O phenate and O-Fe-O bridge-type coordination structures, contributing to a more reductive Fe species and a more oxidizing DHT ligand. Moreover, the lowest energy gap is assigned to the HOMO/LUMO of the O-Fe-O bridge-type coordination structure, indicative of its highest chemical reactivity favorable for the electron transfer. According to the HOMO/LUMO of the hexacoordinated Fe-DHT conformation and the above discussions, compared to the Fe-O carboxylate coordinated DHT, the O-Fe-O bridge-type coordinated DHT in the hexacoordinated conformation will be more easily oxidized for the Fe reduction.

Supplementary Table 7. Calculated bond dissociation energy (BDE) and coordination structure between DFOM and Cu^{2+} or Fe^{3+}.

Metal ion	Bond dissociation energy (a.u.)	Coordination structure
Cu^{2+}	0.599	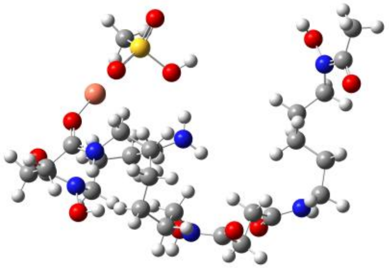
Fe^{3+}	1.642	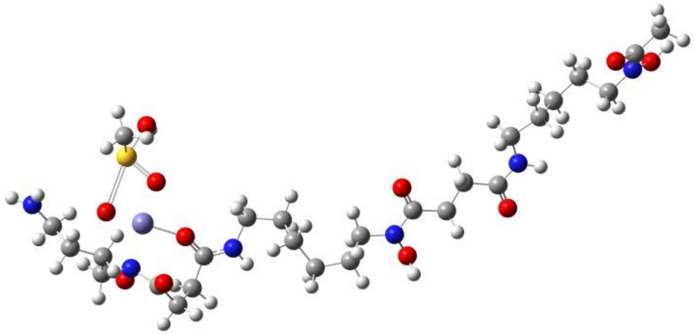

According to the DFT calculations, DFOM shows the higher BDEs toward metal ions (e.g., Cu^{2+}, Fe^{3+}) than DHT. Surprisingly, the BDE between DFOM and Fe^{3+} in a single coordinated mode is higher than that between DHT and Fe^{3+} in the optimized hexacoordinated mode, proving the more excellent Fe chelating ability of DFOM.

Supplementary Table 8. The primer sequences used for RT-qPCR experiments

Gene	Forward primer sequence (5'-3')	Reverse primer sequence (5'-3')
Nanog	TCTTCCTGGTCCCCACAGTTT	GCAAGAATAGTTCTCGGGATGAA
Sox2	GCACATGAACGGCTGGAGCAACG	TGCTGCGAGTAGGACATGCTGTAGG
Oct4	TGGCGTGGAGACTTTGCA	GAGGTTCCCTCTGAGTTGCTTTC
Ctnnb1	GCTGTCCTATTCCGAATGTCTGAG	GGCACCAATGTCCAGTCCAAG
Fibronectin-1	CTATAGGATTGGAGACACGTGG	CTGAAGCACTTTGTAGAGCATG
Smad3	ATTCCATTCCCGAGAACACTAA	TAGGTCCAAGTTATTGTGTGCT
Snail	CCGCAGGTGGCTGATGGAG	CAGTGGGTTGGCTTTAGTTCTATGG
Bcl-2	TACGAGTGGGATGCTGGAGATG	TCAGGCTGGAAGGAGAAGATGC
Bax	ATGGGCTGGACACTGGACTTC	GGTGAGCGAGGCGGTGAG
TfR	GGCTCTGGCTCTCACACTCTC	GGCATTTGCGACTCCCTGAATAG
Chac1	CCGTGCTTGGTGGCTATGAC	ATCTGTGTGGCAATGACCTCTTC
Ptgs2	GTGCCTGGTCTGATGATGTATGC	TGAGTCTGCTGGTTTGGAATAGTTG
Gapdh	TGACATCAAGAAGGTGGTGAAGCAG	CGGCATCGAAGGTGGAAGAGTG

Supplementary Table 9. List of the used antibodies and their characteristics

Protein	Assay	Primary antibody	Manufacturer	Item number	Dilution ratio
Nanog	Western blot	Rabbit monoclonal	Abcam	ab109250	1:500
Nanog	Immunohistochemistry	Rabbit monoclonal	Abcam	ab109250	1:150
Sox2	Western blot	Rabbit polyclonal	Abcam	ab97959	1:500
Sox2	Immunohistochemistry	Rabbit polyclonal	Abcam	ab97959	1:100
Sox2	Immunofluorescence	Mouse monoclonal	Proteintech	66411-1-Ig	1:200
Oct4	Western blot	Rabbit monoclonal	Abcam	ab200834	1:5000
Oct4	Immunohistochemistry	Rabbit monoclonal	Abcam	ab200834	1:250
β-catenin	Western blot	Rabbit monoclonal	CST	8480S	1:1000
Wnt-1	Western blot	Mouse monoclonal	Santa Cruz	sc-514531	1:1000
Lrp6	Western blot	Rabbit monoclonal	CST	3395T	1:1000
Ferritin	Western blot	Rabbit monoclonal	Abcam	ab75973	1:1000
Fibronectin-1	Western blot	Mouse monoclonal	Proteintech	66042-1-Ig	1:2000
Vimentin	Western blot	Rabbit polyclonal	Proteintech	10366-1-AP	1:2000
N-cadherin	Western blot	Rabbit monoclonal	Abcam	ab76011	1:1000

E-cadherin	Western blot	Rabbit polyclonal	Proteintech	20874-1-AP	1:20000
CD44	Western blot	Mouse monoclonal	Proteintech	60224-1-Ig	1:500
CD44	Immunofluorescence	Rabbit polyclonal	Boster	A00052	1:200
FoxO3	Western blot	Rabbit monoclonal	CST	12829	1:1000
CD133	Western blot	Mouse monoclonal	Proteintech	66666-1-Ig	1:500
c-Myc	Western blot	Rabbit monoclonal	Abcam	ab32072	1:1000
Bcl-2	Western blot	Rabbit polyclonal	Proteintech	26593-1-AP	1:1000
Bax	Western blot	Mouse monoclonal	Proteintech	60267-1-Ig	1:5000
Cleaved caspase-3	Western blot	Rabbit monoclonal	CST	9664	1:1000
GPX4	Western blot	Rabbit monoclonal	Abcam	ab125066	1:1000
GPX4	Immunofluorescence	Rabbit monoclonal	Abcam	ab125066	1:100
TfR	Western blot	Mouse monoclonal	Abcam	ab269513	1:5000
β-actin	Western blot	Rabbit monoclonal	ABclonal	AC026	1:50000
β-actin	Western blot	Mouse monoclonal	Proteintech	66009-1-Ig	1:30000

**Secondary antibody**	**Assay**	**Manufacturer**	**Item number**	**Dilution**
Horseradish peroxidase-conjugated goat anti-rabbit IgG	Western blot	Jackson	111-035-003	1:5000

Horseradish peroxidase-conjugated goat anti-mouse IgG	Western blot	Proteintech	SA00001-1	1:5000
Horseradish peroxidase-conjugated goat anti-rabbit IgG	Immunohistochemistry	Abcam	ab205718	1:2000
Cy3-conjugated AffiniPure goat anti-rabbit IgG (H+L)	Immunofluorescence	Boster	BA1032	1:200
DyLight 488-conjugated AffiniPure goat anti-mouse IgG (H+L)	Immunofluorescence	Boster	BA1126	1:200

References

1. Funke H, Chukalina M, Scheinost AC. A new FEFF-based wavelet for EXAFS data analysis. *J Synchrotron Radiat* 2007; **14**: 426-32.
2. Ravel B, Newville M. ATHENA,ARTEMIS,HEPHAESTUS: data analysis for X-ray absorption spectroscopy using IFEFFIT. *J Synchrotron Radiat* 2005; **12**: 537-41.
3. Robinson MD, McCarthy DJ, Smyth GK. edgeR: a Bioconductor package for differential expression analysis of digital gene expression data. *Bioinformatics* 2010; **26**: 139-40.
4. Shannon P, Markiel A, Ozier O *et al*. Cytoscape: a software environment for integrated models of biomolecular interaction networks. *Genome Res* 2003; **13**: 2498-504.
5. Ottewell PD, Woodward JK, Lefley DV *et al.* Anticancer mechanisms of doxorubicin and zoledronic acid in breast cancer tumor growth in bone. *Mol Cancer Ther* 2009; **8**: 2821-32.
6. Sui X, Huang X, Pu H *et al.* Tailoring MOF-derived porous carbon nanorods confined red phosphorous for superior potassium-ion storage. *Nano Energy* 2021; **83**: 105797.
7. Millward AR, Yaghi OM. Metal-organic frameworks with exceptionally high capacity for storage of carbon dioxide at room temperature. *J Am Chem Soc* 2005; **127**: 17998-9.
8. Cozzolino AF, Brozek CK, Palmer RD *et al.* Ligand redox non-innocence in the stoichiometric oxidation of Mn_2(2,5-dioxidoterephthalate) (Mn-MOF-74). *J Am Chem Soc* 2014; **136**: 3334-7.
9. Shiozawa H, Melnikova Z, Bastl Z *et al*. Electrochromic 2,5-dihydroxyterephthalic acid linker in metal-organic frameworks. *Adv Photonics Res* 2022; **3**: 2100219.
10. Mai TT, Hamai A, Hienzsch A *et al.* Salinomycin kills cancer stem cells by sequestering iron in lysosomes. *Nat Chem* 2017; **9**: 1025-33.
11. Torti SV, Torti FM. Iron and cancer: more ore to be mined. *Nat Rev Cancer* 2013; **13**: 342-55.
12. El Hout M, Dos Santos L, Hamai A *et al.* A promising new approach to cancer

therapy: targeting iron metabolism in cancer stem cells. *Semin Cancer Biol* 2018; **53**: 125-38.

13. Rennick JJ, Johnston APR, Parton RG. Key principles and methods for studying the endocytosis of biological and nanoparticle therapeutics. *Nat Nanotechnol* 2021; **16**: 266-76.

14. Lamouille S, Xu J, Derynck R. Molecular mechanisms of epithelial-mesenchymal transition. *Nat Rev Mol Cell Biol* 2014; **15**: 178-96.

15. Gazaryan IG, Krasinskaya IP, Kristal BS *et al.* Zinc irreversibly damages major enzymes of energy production and antioxidant defense prior to mitochondrial permeability transition. *J Biol Chem* 2007; **282**: 24373-80.

16. Chen X, Comish PB, Tang D *et al.* Characteristics and biomarkers of ferroptosis. *Front Cell Dev Biol* 2021; **9**: 637162.

17. Dai Y, Yang Z, Cheng S *et al.* Toxic reactive oxygen species enhanced synergistic combination therapy by self-assembled metal-phenolic network nanoparticles. *Adv Mater* 2018; **30**: 1704877.

18. Huang C, Hu F, Song D *et al.* EZH2-triggered methylation of SMAD3 promotes its activation and tumor metastasis. *J Clin Invest* 2022; **132**: e152394.
